# Safety and serum distribution of anti-SARS-CoV-2 monoclonal antibody MAD0004J08 after intramuscular injection

**DOI:** 10.1038/s41467-022-29909-x

**Published:** 2022-04-27

**Authors:** Simone Lanini, Stefano Milleri, Emanuele Andreano, Sarah Nosari, Ida Paciello, Giulia Piccini, Alessandra Gentili, Adhuna Phogat, Inesa Hyseni, Margherita Leonardi, Alessandro Torelli, Emanuele Montomoli, Andrea Paolini, Andrea Frosini, Andrea Antinori, Emanuele Nicastri, Enrico Girardi, Maria Maddalena Plazzi, Giuseppe Ippolito, Francesco Vaia, Giovanni Della Cioppa, Rino Rappuoli

**Affiliations:** 1 Istituto Nazionale per le Malattie Infettive Lazzaro Spallanzani – IRCCS, Rome, Italy; 2 Centro Ricerche Cliniche di Verona, University and Hospital Trust of Verona, Verona, Italy; 3 Monoclonal Antibody Discovery (MAD) Lab, Fondazione Toscana Life Sciences, Siena, Italy; 4 AchilleS Vaccine, Siena, Italy; 5 VisMederi S.r.l, Siena, Italy; 6 CROss Research, Mendrisio, Switzerland; 7 Fondazione Toscana Life Sciences, Siena, Italy; 8 VisMederi Research S.r.l, Siena, Italy; 9 Department of Molecular and Developmental Medicine, University of Siena, Siena, Italy; 10 Toscana Life Sciences Sviluppo, Siena, Italy; 11 Clinical R&D Consultants, Rome, Italy; 12 Department of Biotechnology, Chemistry and Pharmacy, University of Siena, Siena, Italy

**Keywords:** Drug development, Antibodies, Phase I trials, SARS-CoV-2

## Abstract

The emerging threat represented by SARS-CoV-2 variants, demands the development of therapies for better clinical management of COVID-19. MAD0004J08 is a potent Fc-engineered monoclonal antibody (mAb) able to neutralize in vitro all current SARS-CoV-2 variants of concern (VoCs) including the omicron variant even if with significantly reduced potency. Here we evaluated data obtained from the first 30 days of a phase 1 clinical study (EudraCT N.: 2020-005469-15 and ClinicalTrials.gov Identifier: NCT04932850). The primary endpoint evaluated the percentage of severe adverse events. Secondary endpoints evaluated pharmacokinetic and serum neutralization titers. A single dose administration of MAD0004J08 via intramuscular (*i.m*.) route is safe and well tolerated, resulting in rapid serum distribution and sera neutralizing titers higher than COVID-19 convalescent and vaccinated subjects. A single dose administration of MAD0004J08 is also sufficient to effectively neutralize major SARS-CoV-2 variants of concern (alpha, beta, gamma and delta). MAD0004J08 can be a major advancement in the prophylaxis and clinical management of COVID-19.

## Abstract (web summary)

Monoclonal antibodies (mAbs) are a potential therapeutic option for treatment of COVID-19. Here, the authors report safety and pharmacokinetics of an intramuscularly injected mAb (MAD0004J08) during the first 30 days of a phase 1 clinical study.

## Introduction

The COVID-19 pandemic highlighted the potential of human monoclonal antibodies (mAbs) to tackle pandemics as they demonstrated to be safe and effective therapeutic tools that can be brought from discovery to proof-of-concept trials in only 5–6 months^1^. Since the start of the pandemic, a dozen mAbs capable of neutralizing the SARS-CoV-2 virus have been identified and are under clinical development. Regulatory agencies have granted Emergency Use Authorization (EUA) to Eli Lilly’s mAb bamlanivimab (LY-CoV555) and mAb combination bamlanivimab + etesevimab, to Regeneron’s mAb combination casiribimab + imdevimab (REGN-COV2), to Celltrion regdanvimab, and GSK/VIR sotrovimab^2–5^. With the emergence of antibody-resistant SARS-CoV-2 variants of concern (VoCs) at the end of 2020, some mAbs lost their clinical efficacy and in April 2021 the FDA revoked EUA for bamlanivimab^6^.

The Toscana Life Sciences Foundation has recently reported the isolation and characterization of the neutralizing mAb, MAD0004J08 from a convalescent COVID-19 patient^7^. MAD0004J08 appears to be one of the best candidates for clinical development as it displays the most desirable characteristics for the development of mAb-based prophylaxis and therapy: (1) unprecedented high neutralizing potency, implying low dose requirement, possibility to be administered intramuscularly (i.m.), and significant cost reduction of cost of goods; (2) breadth of neutralizing activity, in vitro vs. the original Wuhan virus and SARS-CoV-2 VoC; B.1.1.7 (alpha), B.1.351 (beta), P.1 (gamma) and B.1.617.2 (delta) with a potency below 10 ng/mL^7,8^, implying broad clinical usefulness in countries with diverse SARS-CoV-2 epidemiology; (3) engineered immunoglobulin fragment crystallizable (Fc) region (M428L/N434S^9,10^ and L234A/L235A/ P329G^11^) to increase its serum half-life while silencing the Fc activity to abrogate binding to FcγRs and eliminate possible risks of antibody-dependent enhancement (ADE) of disease^7^. ADE mediated by the interaction of antibody and FcγRs was previously described for the closely related SARS-CoV virus following its outbreak in 2002 and therefore considered a high risk also for SARS-CoV-2 infection^12,13^. Due to these properties, MAD0004J08 is expected to accelerate clearance of the virus, not induce proinflammatory cytokine response, and thus prevent clinical deterioration of COVID-19 disease in patients.

This paper reports the results of the first 30 days of an ongoing phase 1, placebo-controlled, double-blind, randomized, single-dose, dose-escalation study to evaluate the safety, pharmacokinetics, and virus neutralization titers of the anti-SARS-CoV-2 monoclonal antibody MAD0004J08 in 30 healthy adults. The data generated thus far suggest MAD0004J08 has a strong potential as a global therapeutic tool against COVID-19.

## Results

**Fig. 1:**
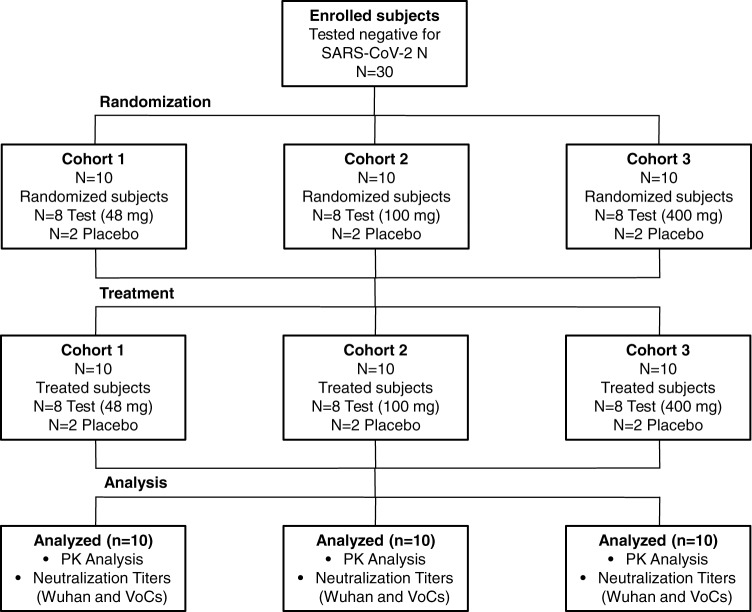
CONSORT diagram phase 1 trial. The graph shows the enrollment of subjects and their allocation to treatment and analysis.

The trial was initiated by screening 50 healthy volunteers between March and May 2021. There was a total of 15 screen failures, 13 due to inclusion/exclusion criteria not met and two to withdrawal. Five screened volunteers were kept as reserves. Thirty subjects who tested negative for SARS-CoV-2 nucleocapsid (N) protein were enrolled and randomized. The serology ant-N test was introduced to exclude subjects presenting endogenous anti-SARS-CoV-2 antibodies induced by the previous infection. Baseline demographic characteristics of the participants in the safety population at enrolment were similar among the treatment groups in terms of sex, mean age, and race (Table 1). Three single ascending doses (48, 100, and 400 mg) and placebo were administered by *i.m*. injection to three study cohorts (cohort 1, cohort 2, and cohort 3 that received 48, 100, and 400 mg, respectively). Eight treated and two placebo subjects were enrolled in each group as described in Fig. 1.

**Table 1:** MAD0004J08 phase I clinical trial summary.

	Cohort 1 (*n* = 10)	Cohort 2 (*n* = 10)	Cohort 3 (*n* = 10)
Age, years	32.2	26.4	38.2
Min/Max	19–51	21–33	27–54
≥18–<30	4 (40%)	7 (70%)	3 (30%)
≥30–<40	3 (30%)	3 (30%)	2 (20%)
≥40	3 (30%)	0 (0%)	5 (50%)
Sex			
Male	5 (50%)	6 (60%)	6 (60%)
Female	5 (50%)	4 (40%)	4 (40%)
Ethnicity			
White	10 (100%)	10 (100%)	10 (100%)
Nucleocapsid (N) serology test			
Tested positive	0 (0%)	0 (0%)	0 (0%)
Tested negative	10 (100%)	10 (100%)	10 (100%)
Administered dosage			
48 mg	8 (80%)	0 (0%)	0 (0%)
100 mg	0 (0%)	8 (80%)	0 (0%)
400 mg	0 (0%)	0 (0%)	8 (80%)
Placebo	2 (20%)	2 (20%)	2 (20%)
Systemic adverse events			
Headache	4 (40%)	3 (30%)	3 (30%)
Fatigue	4 (40%)	1 (10%)	1 (10%)
New or worsen muscle pain	0 (0%)	0 (0%)	1 (10%)
New or worsen joint pain	0 (0%)	0 (0%)	0 (0%)
Nausea (mild)	2 (20%)	0 (0%)	0 (0%)
Asthenia	0 (0%)	1 (10%)	0 (0%)
Vomiting	0 (0%)	0 (0%)	0 (0%)
Diarrhea	0 (0%)	0 (0%)	0 (0%)
Chills	0 (0%)	0 (0%)	0 (0%)
Fever	0 (0%)	0 (0%)	0 (0%)
Local adverse events			
Pain at injection site	1 (10%)	0 (0%)	0 (0%)
Redness	1 (10%)	0 (0%)	0 (0%)
Swelling	0 (0%)	0 (0%)	0 (0%)
Severity of reported adverse events			
Mild	11 (91%)	5 (100%)	3 (60%)
Moderate	1 (9%)	0 (0%)	2 (40%)
Severe	0 (0%)	0 (0%)	0 (0%)
Pharmacokinetics ng/mL (95% CI)^a^			
Day 1 (0 h)	6.9	6.6	11.4
Day 1 (1 h)	22.6	36.3	43.1
Day 1 (2 h)	92.3	257.4	97.5
Day 1 (3 h)	203.1	434.7	835.3
Day 1 (4 h)	396.7	786.6	1740.8
Day 1 (6 h)	571.8	1259.1	3320.5
Day 1 (8 h)	691.8	1650	4922.1
Day 1 (12 h)	1017.3	2471.9	6695.5
Day 1 (24 h)	1543.4	3185.5	9900.2
Day 2	3119.7	6241.4	21,581.4
Day 8	5124.9	11,034.7	38,017.2
Day 15	5988.0	11,998.7	44,279.2
Day 22	6640.9	11,691.5	47,022.6
Day 30	6552.9	11,993.9	44,579.3
Day 60	6058.8	9856.5	31,624.6
GMT neutralization (95% CI)^a^			
Original Wuhan (48 h)	334.2	334.2	1173.8
Original Wuhan (day 8)	493.5	1076.3	2061.4
Original Wuhan (day 30)	397.4	562.0	1395.8
B.1.1.7 (alpha) (day 8)	306.4	794.8	4122.8
B.1.351 (beta) (day 8)	134.5	269.1	1291.4
P.1 (gamma) (day 8)	113.1	198.7	697.9
B.1.617.2 (delta) (day 8)	472.6	905.1	5120.0

Data are mean, *n*, *n* (%), GM ng/mL (95% CI), or GMT (95% CI). Adverse events severity is reported as mild, moderate, and severe.

^a^Data relative to placebo groups were not reported in the table as subjects tested negative for all the pharmacokinetics and serum neutralization analyses performed in this study.

Overall, the study population consisted of white healthy male and female participants with a mean age of 32.2 years (range 19–54 years); 56.6% were male and 43.4% were female (Table 1).

The study has now been unblinded and treated/placebo subjects allocated to their respective groups. Subjects from the placebo group tested negative for all tests performed in this study and therefore were not included in tables and figures.

No severe or serious treatment-emergent adverse event (TEAE) was reported through 7 days post dosing. Local and systemic solicited adverse events through day 7 occurred in a few subjects (Table 1), were all mild to moderate, lasted no more than 2 and 6 days for systemic and local TEAE respectively, and showed no sign of dose-related increase of frequency or severity. Overall, systemic solicited adverse events (*n* = 20; 90.1%) were more frequent than local solicited adverse events (*n* = 2; 9.1%) (Table 1).

**Fig. 2:**
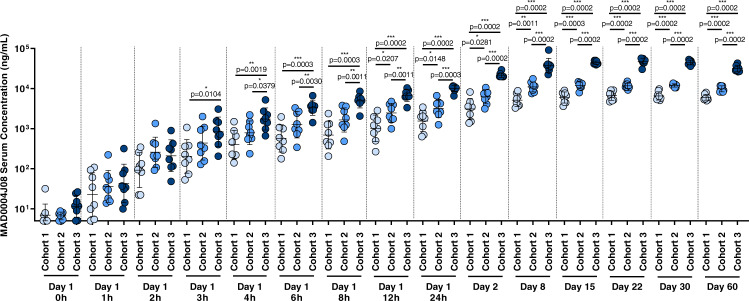
MAD0004J08 pharmacokinetics. SARS-CoV-2 spike (S) protein IgG mean titers in (ng/mL) pre and post MAD0004J08 injection in three cohorts. Dots indicate the individual antibody titers for Cohort 1 (48mg-light blue; *n* = 8), Cohort 2 (100mg-blue; *n* = 8) and Cohort 3 (400mg-dark blue; *n* = 8). Only *p* values for statistically significant differences are shown in the figure. A nonparametric Mann–Whitney *t* test was used to evaluate statistical significance between groups. Two-tailed *p* value significances are shown as *p ≤ 0.05, **p ≤ 0.01, and ***p ≤ 0.001. Data are presented as geometric mean ± standard deviation. Subjects from the placebo group (2/group) were excluded from the analyses.

The baseline (pre-dose) geometric mean (GM) concentrations at day 1 (0 h) of anti-spike antibodies measured by ELISA were at background levels. Subjects in the placebo group had GM levels below the lower limit of quantification (LLOQ) at all timepoints. As for dosed subjects, detectable levels of antibodies were seen as quickly as two hours post-administration and increased constantly in a dose-dependent manner. On day 8, mean sera binding titers almost peaked showing a GM of 5124.9, 11,034.7, and 38,017.2 ng/mL for cohorts 1, 2, and 3, respectively. Sera binding titers continued to increase up to 30 days with a GM of 6552.9, 11,993.9, and 44,579.3 ng/mL for cohorts 1, 2, and 3, respectively, and maintained high titers up to 60 days with a GM of 6058.8, 9856.5, and 31,624.6 ng/mL for cohorts 1, 2, and 3, respectively (Fig. 2, Table 1, and Supplementary Table 1). Maximum serum concentration (Cmax) and Time of Maximum concentration observed (Tmax) were evaluated and reported as GM of all treated subjects (*n* = 8) per group. GM-Cmax were 7098.4, 13,472.8, and 52,907.9 ng/mL for cohort 1, 2, and 3, respectively, showing a dose-dependent response. GM-Tmax was 27.2, 17.2, and 18.1 days for cohorts 1, 2, and 3, respectively. While Cohort 2 and 3 show a very similar GM-Tmax, cohort 1 showed a 1.5-fold slower distribution.

The study assessed the neutralizing activity of sera from MAD0004J08 dosed subjects against SARS-CoV-2 original Wuhan virus. The results are represented as geometric mean neutralization titers (GMT) leading to 100% inhibitory dilution (ID_100_). The GMT in MAD0004J08 administered subjects at baseline was below the lower limit of quantitation (LLOQ—<10). Placebo subjects had ID_100_ levels below the LLOQ at all study points. The GMT levels in cohort 1 (48 mg) on day 2 were 334.2, increased to 493.5 on day 8, and was 397.4 on day 30. The GMT in cohort 2 (100 mg) was 334.2 on day 2, improved further to 1076.3 on day 8, and was 562.0 on day 30. The GMT levels for cohort 3 (400 mg) were 1173.8, 2061.4, and 1395.8 on days 2, 8, and 30 respectively (Table 1 and Supplementary Table 2).

**Fig. 3:**
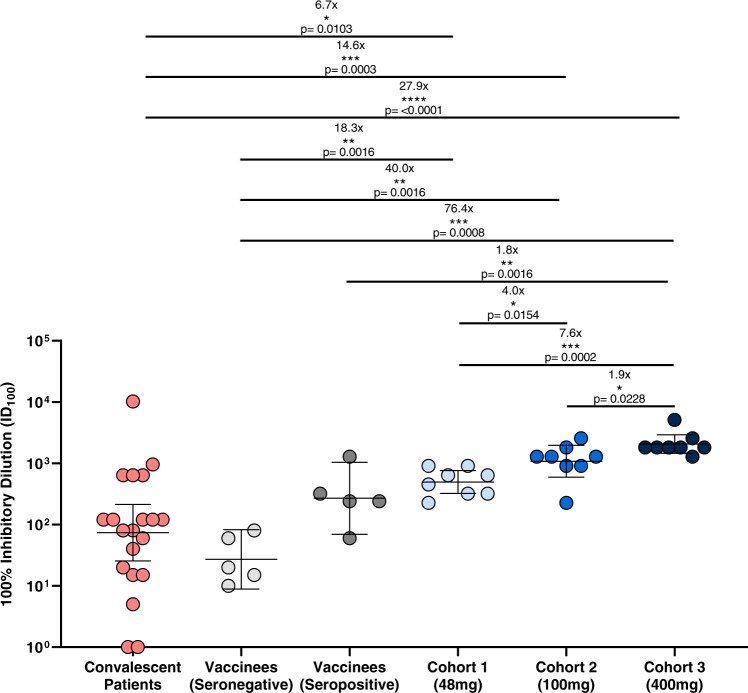
Serum neutralization activity against SARS-CoV-2 Wuhan virus. The graph shows the neutralization reported as 100% inhibitory dilution (ID_100_) of sera collected from COVID-19 convalescent patients (*n* = 20; red), average samples collection 10 days post-hospitalization, vaccinees not exposed (seronegative; *n* = 5; light gray), average samples collection 48 days post-vaccination, or previously exposed (seropositive; *n* = 5; dark gray), average samples collection 21 days post-vaccination, to SARS-CoV-2 and subjects that received MAD0004J08 at 48 (*n* = 8; light blue), 100 (*n* = 8; blue), and 400 mg (*n* = 8; dark blue) at day 8. Only *p* values for statistically significant differences are shown in the figure. A nonparametric Mann–Whitney *t* test was used to evaluate statistical significance between groups. Two-tailed *p* value significances are shown as *p ≤ 0.05, **p ≤ 0.01, ***p ≤ 0.001, and ****p ≤ 0.0001. Data are presented as geometric mean ± standard deviation.

Additionally, we compared the neutralizing activity against the original Wuhan virus of sera from MAD0004J08 dosed subjects with COVID-19 convalescent patients (*n* = 20) and two groups of BNT162b2 mRNA vaccine (Comirnaty, Pfizer/BioNTech) recipients (*n* = 5/group) who were either seronegative or seropositive for SAR-CoV-2 before vaccination. The GMT for 20 COVID-19 convalescent patients had a wide range of 1–10,240, which can be explained by differences in clinical severity of COVID-19 disease as 15/20 (75%) were hospitalized and 10/15 (66.7%) of hospitalized patients were on oxygen therapy. Of the 15 hospitalized patients, 4/15 (26.7%) had a severe infection (with acute respiratory distress syndrome), 6/15 (40.0%) had a moderate infection (oxygen therapy), and the remaining 5/15 (33.3%) had a mild infection (paucisymptomatic). The average GMT for all 20 COVID-19 convalescent patients was 73.7, whereas the average GMT for seronegative and seropositive vaccinees was 27.0 and 269.3, respectively (Fig. 3 and Supplementary Table 3). Overall, the average neutralizing ability of sera from cohorts 1, 2, and 3 subjects on day 8 were 6.7-, 14.6-, and 27.9-fold significantly higher than COVID-19 convalescent sera and were 18.3-, 40.0-, 76.4-, and 1.83-, 4.0-, 7.6-fold higher compared to seronegative and seropositive vaccinees, respectively (Fig. 3, Table 1, and Supplementary Table 3).

**Fig. 4:**
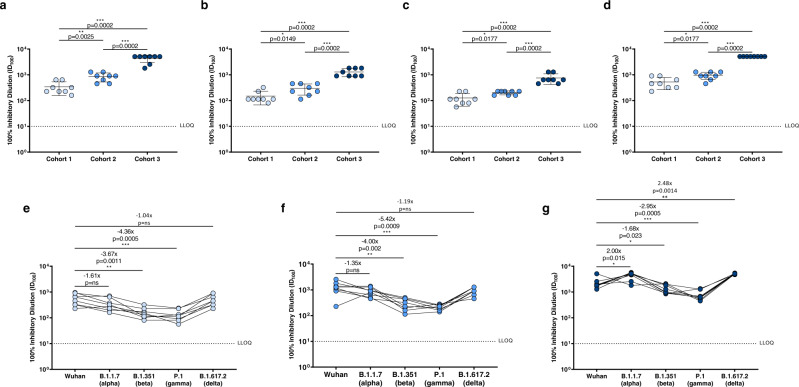
Serum neutralization activity against SARS-CoV-2 variants of concern. **a**–**d** Graphs show the neutralization activity reported as 100% inhibitory dilution (ID_100_) of sera collected from subjects who received MAD0004J08 at 48 (cohort 1; *n* = 8; light blue), 100 (cohort 2; *n* = 8; blue) and 400 (cohort 3; *n* = 8; dark blue) mg against SARS-CoV-2 B.1.1.7 (alpha), B.1.351 (beta), P.1 (gamma), and B.1.617.2 (delta) variants at day 8. Data are presented as geometric mean ± standard deviation. **e**–**g** Graphs show values of serum neutralization activity against the different SARS-CoV-2 VoCs tested in this study for Cohort 1 (*n* = 8), Cohort 2 (*n* = 8), and Cohort 3 (*n* = 8). Fold changes relative to Wuhan virus are reported for each VoCs. A nonparametric Mann–Whitney *t* test was used to evaluate statistical significances between groups and tested viruses. Two-tailed *p* value significances are shown as *p ≤ 0.05, **p ≤ 0.01, and ***p ≤ 0.001. Data are presented as geometric mean ± standard deviation. Data are presented as geometric mean.

Finally, we evaluated the sera neutralization activity on day 8 of all subjects that received MAD0004J08 against the SARS-CoV-2 variants of concern B.1.1.7 (alpha), B.1.351 (beta), P.1 (gamma), and B.1.617.2 (delta) (Fig. 4). Average GMT and 95% confidence interval (95% CI) are reported in Table 1 and Supplementary Table 2. Overall, a single i.m. injection of MAD0004J08 resulted in high neutralization titers against all tested SARS-CoV-2 variants in a dose-dependent fashion with average GMT levels for cohort 1 of 306.4, 134.5, 113.1, and 472.6, for cohort 2 of 794.8, 269.1, 198.7 and 905.1, and for cohort 3 of 4122.8, 1291.4, 697.9, and 5120.0 against the B.1.1.7, B.1.351, P.1 and B.1.617.2 respectively (Fig. 4a–d, Table 1, and Supplementary Table 2). Average GMT levels against the B.1.1.7 (alpha) and B.1.617.2 (delta) variants were comparable to the SARS-CoV-2 Wuhan virus for cohorts 1 and 2, while the GMT was 2- and 2.48-fold higher for cohort 3 against B.1.1.7 (alpha), and B.1.617.2 (delta), respectively (Fig. 4e–g). Average neutralization titers compared to the original Wuhan virus dropped by 3.67-, 4.00-, and 1.68-fold against B.1.351 (beta) and by 4.36-, 5.42-, and 2.95-fold against P.1 (gamma) for cohort 1, 2, and 3 respectively (Fig. 4e–g, Table 1, and Supplementary Table 2). However, despite reductions in neutralization titers were observed against the variants, GMT levels were either comparable, in case of cohort 1 (48 mg), or significantly higher, for cohorts 2 and 3 (100 and 400 mg), than those observed in convalescent patients and vaccinees against the Wuhan virus (Figs. 3 and 4).

## Discussion

The results of this study show that, following a single i.m. administration, MAD0004J08 at 48, 100, and 400 mg is safe, well tolerated with minimal reactogenic adverse events, and rapidly distributes systemically. In addition, a single dose of MAD0004J08 resulted in extremely high serum neutralization titers which were 5–76-fold higher compared to COVID-19 convalescent donors and BNT162b2 mRNA vaccinees. With the emergence and worldwide spread of SARS-CoV-2 VOCs, it is imperative to develop mAbs able to neutralize circulating variants of concern (VoC), therefore maintaining their therapeutic efficacy. Some mAbs already in clinical application for the treatment of COVID-19, showed to fall short against emerging variants, and for one of them, due to lack of clinical efficacy, regulatory agencies revoked emergency use authorization^6^. In addition, with the emergence of the SARS-CoV-2 omicron variant and its sublineages, all antibodies except one lost their neutralization activity and consequent clinical effectiveness against this virus, and MAD0004J08 was also significantly impacted, but not completely evaded, by this variant^14^. In this study, we showed that sera from MAD0004J08 treated subjects retained high neutralization titers against major SARS-CoV-2 VoCs including the B.1.1.7 (alpha), B.1.351 (beta), P.1 (gamma), and B.1.617.2 (delta) variants highlighting the potential of this antibody to be used globally for the treatment of COVID-19. High antibody neutralization titers were previously shown to be predictive of immune protection from SARS-CoV-2 infection and long-term persistence of neutralizing antibodies was shown to estimate the duration of protection predicting disease severity and survival^15–18^. Although sera from 400 mg dose showed the highest neutralization titers against SARS-CoV-2 and variants of concern, the 100 mg dose, which was also found to have significantly higher neutralizing titers compared to convalescent patients and vaccinees, could be sufficient and strategically more suitable to develop. In fact, a single 100 mg dose of MAD0004J08 could increase the number of available doses to give to COVID-19 patients.

To complement the ability of MAD0004J08 to neutralize major SARS-CoV-2 VoC, the Fc modifications introduced to extend its serum half-life and to limit the risk of ADE in hospitalized COIVD-19 patients could further increase the clinical suitability of this antibody. Concerning the serum half-life extension, our data showed that sera neutralization titers were maintained at high levels up to the 60 days post-administration and follow-up is planned for up to 6 months. As for the silencing of the Fc-portion to minimize risks of ADE our strategy, compared to the use of unmodified or enhanced Fc-functions in antibodies already in the clinic, could result to be successful in hospitalized COVID-19 patients with and without high titers of serum antibodies against SARS-CoV-2. In fact, at the end of 2020, the Eli Lilly bamlanivimab (LY-CoV555) received emergency use authorization but the application was limited as the administration of this antibody may be associated with worse clinical outcomes when administered to hospitalized patients requiring high flow oxygen or mechanical ventilation with COVID-19^5^. In addition, a recent primary clinical trial under peer-review has shown that intravenous administration of REGEN-COV mAb cocktail, at a total dose of 8 g in hospitalized COVID-19 patients reduced 28-day mortality among patients who were seronegative at baseline, highlighting the important role of mAbs therapy even in hospitalized COVID-19 patients^19^. However, COVID-19 seropositive hospitalized patients did not benefit from REGEN-COV administration, suggesting that presence of high antibody titers induced by infection plus the infusion of the enormous quantity of mAbs for therapy may not be beneficial for these patients. A recent paper describing the longitudinal neutralizing antibodies in patients progressing to severe disease indicates ADE as one of the mechanisms of increased disease severity^20^. These previous reports indicate that removing Fc-functions could result to be a successful strategy for the treatment of hospitalized seronegative as well as seropositive COVID-19 patients.

The limitation of our study is that our first in human study was conducted in a healthy population of age 18–55 years without much diversity, whereas the risk for severity of COVID-19 disease is more in populations with comorbid conditions, older age groups, and in non-white populations. Our ongoing Phase 2–3 study will assess MAD0004J08 in mild/moderate diseased and stratified subjects groups to assess dose selection and efficacy.

To conclude, MAD0004J08 administration is safe, confers broad coverage against major SARS-CoV-2 variants of concern, and gives the low dosage needed and i.m. route of administration can be a globally available and affordable countermeasure to the COVID-19 pandemic^21^.

## Methods

### Cohort of healthy adults treated with MAD0004J08

Our phase 1, first in human (FIH) trial is underway at two sites in Italy (Istituto Nazionale Malattie Infettive Lazzaro Spallanzani, Rome, and Centro Ricerche Cliniche di Verona s.r.l. (CRC), Verona). The final protocol and informed consent were approved by the institutional review boards of each of the participating investigational sites. The study was approved by the “Comitato Etico Unico dell’Istituto nazionale per le malattie infettive (INMI) Lazzaro Spallanzani”, Rome (IT), ethic committee. This study is designed and conducted in accordance with the Declaration of Helsinki, the current revision of Good Clinical Practice (GCP), ICH topic E6 (R2), and the applicable local law requirements. The trial was registered with EudraCT N.: 2020-005469-15 on the 16th of November 2020 and ClinicalTrials.gov Identifier: NCT04932850. This is a dose-escalation study, open-label across doses and randomized, double blind within each dose level. A total of 30 healthy men and nonpregnant women, 18–55 years of age, meeting all inclusion/exclusion criteria were enrolled in three sequential cohorts of 10 subjects each. Within each cohort, subjects were randomized with 4:1 ratio to a single i.m. dose of MAD0004J08 (48 mg in Cohort 1, 100 mg in Cohort 2, and 400 mg in Cohort 3) or placebo using an interactive web response system (IWRS). Within each cohort, subjects were grouped into two groups of five: the five subjects of the first group, referred to as “sentinels” were randomized one at a time at 48-hour intervals, no safety concern was identified by the investigator; the five subjects of the second group were randomized and enrolled with no time restriction. An Independent Data Safety Monitoring Board (DSMB) recommended progress from Cohort 1 to Cohort 2 and from Cohort 2 to Cohort 3 based pre-defined criteria^22^. Each subject is to undergo 12 visits (V) over a 6-month period: V1-V2 for screening procedures, V1–V3 (Days 1–3) as inpatient in the study center, and V4-V12 as outpatient for follow-up. On day 1 (V3), a single 5 mL injection was administered in the right gluteus for cohorts 1 and 2 and two 5 mL injections, were administered one in the right and one left gluteus for cohort 3. Each subject was provided with two diaries to self-report and record solicited adverse events (AEs) from day 1 to day 7, and unsolicited AEs and concomitant medication throughout the study. The study is now unblinded and subjects were properly allocated to their respective treatment groups.

### Eligibility criteria

A detailed list of inclusion/exclusion criteria can be found in the trial protocol. Key inclusion criteria were as follows: age 18-55, signed informed consent, willingness to use appropriate contraception, body mass index 18.5–30 kg/m^2^; systolic blood pressure 90–139 mmHg, diastolic blood pressure 69–90 mmHg; heart rate 50–100 bpm; electrocardiogram (ECG) without clinically significant abnormalities; negative SARS-CoV-2 serology test (negative anti-S and anti-N) or negative SARS-CoV-2 qRT-PCR in the 72 h prior with the result before the treatment. Serology tests, performed to exclude the presence of endogenous anti-SARS-CoV-2 antibodies induced by the previous infection, were performed through an automated chemiluminescent immunoassay previously described by the Istituto Nazionale Malattie Infettive Lazzaro Spallanzani, Rome, and through a commercially available kit (Anti-SARS-CoV-2 NCP ELISA (IgG), cat. number EI 2606-9601/9620-2 G) following the manufacturer’s instructions by the Centro Ricerche Cliniche di Verona s.r.l. (CRC), Verona^23^. Key exclusion criteria were as follows: prior intake of investigational or licensed vaccine for the prevention of SARS-CoV-2; history of infection with SARS or MERS; positive or missing pregnancy test at screening or day 1 or lactating women; history of allergic reactions likely to be exacerbated by any component of the investigational product; previous intake of a mAb within 6 months; history of malignancy in the past 5–7 years; Immunodeficiency due to illness, any course of glucocorticoid therapy exceeding 2 weeks; acute illnesses—history of renal, hepatic, gastrointestinal, cardiovascular, respiratory, dermatologic, hematological, endocrine, psychiatric or neurological diseases that may interfere with the aim of the study or increase subjects risks in investigator’s opinion. A statement was included in the study protocol that if during the study, a subject is included in a vaccination list according to the national guidelines, the best option for the subject will be pursued. However, such a situation did not arise during the study.

### Investigational medicinal product (IMP)

MAD0004J08, human monoclonal antibody 100 mg/2.5 mL solution for injection was manufactured by Menarini Biotech, and was characterized and filled, at Istituto Biochimico Italiano (IBI) Lorenzini. The placebo was prepared as a 2.5 mL of a 0.9% w/v sterile solution of sodium chloride (NaCl) in water for injections (WFI) by IBI. The investigational products were stored at 2–8 °C in a dry locked place.

### End points

In this report, results from the following study end points are presented. The full set of endpoints (see protocol) in the complete sample will be presented upon completion of the study. Primary end point: Proportion of subjects with severe and/or serious treatment-emergent adverse events (TEAEs), including clinically relevant laboratory abnormalities, vital signs, and adverse reactions at the injection site) in the 7 days post-treatment. A TEAE is defined as any AE with onset after administration of the study drug (*N* = 30).

Secondary end points: Proportion of subjects with solicited local AEs (pain, redness, and swelling at the injection site) and systemic AEs (headache, fatigue, muscle pain, joint pain, vomiting, diarrhea, chills, fever) from day 1 to day 7 (*N* = 30); MAD0004J08 sera concentrations on day 1 (0, 1, 2, 3, 4, 6, 8, 12, and 24 h), days 2, 8, 15, 22, 30 and 60 (N = 24; placebo subjects were excluded from the analyses); MAD0004J08 sera neutralizing ability at baseline, and on days 2, 8, and 30 (*N* = 24; placebo subjects were excluded from the analyses).

### ELISA for PK analyses

The method for detection of MAD0004J08 in human serum is a quantitative sandwich ELISA. Briefly, a 96-wells plate is coated with SARS-CoV-2 Spike protein; after blocking of the plate with PBS + 1% BSA, samples containing MAD0004J08 are pipetted into the wells, followed by a wash with PBS + 0.05% Tween-20 to remove all unbound matrix components; alkaline phosphatase labeled anti-Human IgG (γ chain specific) diluted 1:2000 is added to bind to the immobilized MAD0004J08; the complex is detected by pNPP substrate; after a wash to remove unbound reagents, the enzyme is revealed by its action on the pNPP substrate; after stopping the reaction with a strong base, the intensity of the color (read at 405 nm) is directly proportional to the amount of MAD0004J08 present in the sample. Data were collected by using the BioTek Gen5 Data Analysis Software v3.0.

### SARS-CoV-2 viruses

The SARS-CoV-2 viruses used to perform the CPE-MN neutralization assay were the original Wuhan SARS-CoV-2 virus (SARS-CoV-2/INMI1-Isolate/2020/Italy: MT066156), SARS-CoV-2 B.1.1.7 (INMI GISAID accession number: EPI_ISL_736997), SARS-CoV-2 B.1.351 (EVAg Cod: 014V-04058), P.1 (EVAg CoD: 014V-04089), and SARS-CoV-2 B.1.617.2 (GISAID ID: EPI_ISL_2029113).

### SARS-CoV-2 neutralization assay

SARS-CoV-2 neutralization assay was performed as previously described^7^. Briefly, plasma samples were tested at a starting dilution of 1:10 and then diluted in steps of 1:2 for twelve points. All samples were mixed with a SARS-CoV-2 wild type, SARS-CoV-2 B.1.17 (alpha), B.1.351 (beta), P.1 (gamma), and B.1.617.2 (delta) viral solution containing 100 TCID_50_ of the virus. After 1 h incubation at 37 °C, 5% CO_2_, virus–mAb mixture was added to the wells of a 96-well plate containing a sub-confluent Vero E6 cell monolayer. Plates were incubated for 3–4 days at 37 °C in a humidified environment with 5% CO_2_, then examined for CPE by means of an inverted optical microscope. Technical duplicates were performed for each experiment. Neutralizing activities are represented as geometric mean neutralization titers (GMT) leading to 100% viral neutralization/inhibition (ID_100_). In the original protocol, only GMT against the wild-type (WT) Wuhan strain was envisaged. As post hoc analyses, in light of the evolving epidemiology GMTs vs. alpha, beta, gamma, and delta variants of concern were also assessed. In addition, as post hoc analyses, GMTs following administration of MAD0004J08 were compared to matching GMTs obtained from pools of convalescent and vaccinated sera.

### Plasma specimens from COVID-19 convalescent and vaccinated subjects

Plasma specimens from COVID-19 convalescent subjects and SARS-COV-2 vaccinated subjects used as comparators in the present paper were previously collected from a separate study conducted in collaboration with the National Institute for Infectious Diseases, IRCCS – Lazzaro Spallanzani Rome (IT) and Azienda Ospedaliera Universitaria Senese, Siena (IT). The study was approved by local ethics committees and conducted according to good clinical practice Plasma neutralization titers of COVID-19 convalescent subjects and vaccinated donors were previously reported^24,25^.

### Statistical analysis

To assess the pharmacokinetics of MAD0004J08 in the three different cohorts, a non-parametric Mann–Whitney *T* test was performed among groups at all different time points. For the analyses of sera or plasma neutralization antibody titers against SARS-CoV-2 and VoCs, the 100% inhibitory dilution (ID_100_) was calculated as the geometric mean of two technical duplicates, and the statistical significance of the differences among groups was determined by non-parametric Mann–Whitney *T* test using the GraphPad Prism software (version 8.0.2). Two-tailed *p* value significances are shown as *p ≤ 0.05, **p ≤ 0.01, ***p ≤ 0.001, and ****p ≤ 0.0001.

### Reporting summary

Further information on research design is available in the Nature Research Reporting Summary linked to this article.

## Supplementary information

Supplementary Information

Peer Review File

Reporting Summary

## Data Availability

All data supporting the findings in this study are available within the article, Supplementary Information, or can be obtained from the corresponding author upon request. SARS-CoV-2 sequences are accessible within the global initiative on sharing all influenza data (GISAID) database. Source data are provided with this paper.

## Source data

Source Data
