# Supplementary material for: Safety and serum distribution of anti-SARS-CoV-2 monoclonal antibody MAD0004J08 after intramuscular injection
Source: Nat Commun. 2022 Apr 27;13:2263. doi: 10.1038/s41467-022-29909-x (PMC9046195; doi:10.1038/s41467-022-29909-x)
Supplement: Supplementary file 1 — Supplementary Information [file 41467_2022_29909_MOESM1_ESM.pdf]

## SUPPLEMENTARY TABLES

**Supplementary Table 1. MAD0004J08 pharmacokinetics**

| | Cohort 1 (n=8) | Cohort 2 (n=8) | Cohort 3 (n=8) |
|---|---|---|---|
| **Pharmacokinetics ng/mL (95% CI)†** | | | |
| Day 1 (0h) | **6.9** (4.1 – 11.8) | **6.6** (5.5 – 7.9) | **11.4** (6.8 – 19.3) |
| Day 1 (1h) | **22.6** (7.9 – 64.4) | **36.3** (16.8 – 78.4) | **43.1** (17.2 – 108.0) |
| Day 1 (2h) | **92.3** (39.9 – 213.4) | **257.4** (124.5 – 532.4) | **97.5** (97.5 – 452.4) |
| Day 1 (3h) | **203.1** (90.5 – 455.9) | **434.7** (192.6 – 981.0) | **835.3** (412.2 – 1692.5) |
| Day 1 (4h) | **396.7** (199.2 – 790.1) | **786.6** (443.9 – 1393.9) | **1740.8** (1028.5 – 2946.6) |
| Day 1 (6h) | **571.8** (297.1 – 1100.8) | **1259.1** (664.7 – 2385.0) | **3320.5** (2318.2 – 4756.1) |
| Day 1 (8h) | **691.8** (354.2 – 1351.2) | **1650** (922.4 – 2951.5) | **4922.1** (3508.2 – 6905.9) |
| Day 1 (12h) | **1017.3** (535.9 – 1931.3) | **2471.9** (1563.8 – 3907.2) | **6695.5** (5035.2 – 8903.2) |
| Day 1 (24h) | **1543.4** (930.6 – 2559.8) | **3185.5** (2063.9 – 4916.4) | **9900.2** (8511.6 – 11515.4) |
| Day 2 | **3119.7** (1894.3 – 5137.8) | **6241.4** (4452.2 – 8749.7) | **21581.4** (19232.0 – 24217.8) |
| Day 8 | **5124.9** (3799.3 – 6949.7) | **11034.7** (8677.3 – 14032.5) | **38017.2** (27079.8 – 53372) |
| Day 15 | **5988.0** (4572.6 – 7841.5) | **11998.7** (10161.6 – 14167.9) | **44279.2** (40730.0 – 48137.6) |
| Day 22 | **6640.9** (5314.2 – 8298.9) | **11691.5** (10318.8 – 13246.7) | **47022.6** (39768.1 – 55600.5) |
| Day 30 | **6552.9** (5375.7 – 7987.9) | **11993.9** (11108.9 – 12949.4) | **44579.3** (39794.0 – 49940.1) |
| Day 60 | **6058.8** (5269.7 – 6966.0) | **9856.5** (8549.9 – 11362.6) | **31624.6** (27394.1 – 36508.4) |
| **Cmax (ng/mL)** | **7098.4** (5852.4 – 8609.8) | **13472.8** (11864.5 – 15299.2) | **52907.9** (42987.5 – 65117.7) |
| **Tmax (days)** | **27.2** (19.1 – 38.7) | **17.2** (11.5 – 25.7) | **18.1** (12.1 – 27.0) |

Data are GM ng/mL (95% CI) for PK and Cmax. Data are days (95% CI). †Data relative to placebo groups were not reported in the table as subjects tested negative for all the analyses performed in this study.

**Supplementary Table 2. MAD0004J08 serum neutralisation titres**

| | Cohort 1 (n=8) | Cohort 2 (n=8) | Cohort 3 (n=8) |
|---|---|---|---|
| **GMT neutralization titers (95% CI)†** | | | |
| Original Wuhan (48h) | **334.2** (207.7 – 537.8) | **334.2** (250.8 – 445.3) | **1173.8** (809.6 – 1701.6) |
| Original Wuhan (day 8) | **493.5** (320.7 – 759.5) | **1076.3** (590.8 – 1961) | **2061.4** (1461.0 – 2908.0) |
| Original Wuhan (day 30) | **397.4** (281.7 – 560.6) | **562.0** (453.0 – 697.2) | **1395.8** (1034.2 – 1884.0) |
| B.1.1.7 (alpha) (day 8) | **306.4** (200.9 – 467.5) | **794.8** (563.4 – 1121.0) | **4122.8** 2922.0 – 5816.0) |
| B.1.351 (beta) (day 8) | **134.5** (92.1 – 196.6) | **269.1** (173.6 – 417.0) | **1291.4** (919.8 – 1633.0) |
| P.1 (gamma) (day 8) | **113.1** (75.1 – 170.4) | **198.7** (171.0 – 230.8) | **697.9** (498.0 – 978.1) |
| B.1.617.2 (delta) (day 8) | **472.6** (309.8 – 721.0) | **905.1** (664.0 – 1234.0) | **5120.0** (5120.0 – 5120.0) |

Data are GMT (95% CI). †Data relative to placebo groups were not reported in the table as subjects tested negative for all the analyses performed in this study.

**Supplementary Table 3. Convalescent patients and COVID-19 BNT162b2 mRNA vaccinees serum neutralization titres**

| | Convalescent Patients (n=20) | Seronegative Vaccinees (n=5) | Seropositive Vaccinees (n=5) |
|---|---|---|---|
| **GMT neutralization titers (95% CI)†** | | | |
| Original Wuhan | **73.7** (25.5 – 212.7) | **27.0** (8.8 – 82.6) | **269.3** (69.7 – 1040.5) |

Data are GMT (95% CI).

**Supplementary Information file**

**Supplementary Note 1.** Clinical study protocol.

# CLINICAL STUDY PROTOCOL

Study CRO-20-144 - Sponsor code A0001A

EudraCT N.: 2020-005469-15

## COVID-19: A Phase I dose-escalation study to evaluate the safety and pharmacokinetics of anti-SARS-CoV-2 monoclonal antibody MAD0004J08 in healthy adults

*First-in-human, single-dose, dose-escalation, double-blind, placebo-controlled, randomised, safety and pharmacokinetics study*

| | |
|---|---|
| Test Product: | MAD0004J08 100 mg, 2.5 mL solution for injection, Toscana Life Sciences Sviluppo s.r.l. (TLS-S), Siena, Italy |
| Reference product: | Matching placebo, 2.5 mL solution for injection, TLS-S, Siena, Italy |
| Sponsor: | Toscana Life Sciences Sviluppo s.r.l. (TLS-S)<br>Via Fiorentina 1, I-53100 Siena, Italy<br>Phone: +39.0577.231211<br>Fax: +39.0577.43444<br>Email: info@toscanalifesciences.org |
| Principal Investigator clinical centre N. 1 and National study coordinator: | Simone Lanini, MD<br>Istituto Nazionale Malattie Infettive Lazzaro Spallanzani, via Portuense 292, Rome, Italy<br>Phone: +39.06.5517.0923<br>Email: simone.lanini@inmi.it |
| Development phase: | Phase I |
| Version and date: | Final version 5.0, 09JUL21 |

*This study will be conducted in accordance with the current version of Good Clinical Practice (GCP), ICH topic E6 (R2)*

*Property of the sponsor*
*May not be used, divulged, published or otherwise disclosed without the consent of the sponsor*

This document comprises 91 pages

# VERSIONS' HISTORY

| Version | Date | Description of Changes |
|---|---|---|
| 1.0 | 20DEC20 | Original document |
| 2.0 | 25JAN21 | The Clinical Study Protocol was modified according to the recommendations of the Istituto Superiore di Sanita' (ISS), Italy. In particular:<br>➢ An additional cohort, in which subjects will be treated with a dose of 48 mg MAD0004J08, was added before the other two planned cohorts. The study dosing scheme, the rationale for dose selection, the study rationale, the discussion of study design, the randomization number and kit number section, and all other affected protocol sections were modified accordingly.<br>➢ Each cohort is composed of two groups. The subjects enrolled in the first group of each cohort (groups 1, 3 and 5 for Cohorts 1, 2 and 3, respectively) will be sentinel subjects and will be treated one at the time at 48 h intervals in order to evaluate possible treatment-related adverse events. The affected protocol sections were modified accordingly.<br>➢ According to ISS advice, safety evaluation by the Data Safety Monitoring Board will be performed on the 48 h safety data of Cohort 1 and Cohort 2, before proceeding with Cohort 2 and Cohort 3, respectively.<br>➢ Details on the the analytical method for the analysis of MAD0004J0B were added.<br><br>In addition,<br>➢ The following sentence was added: *In case, during the study, a subject is included in a vaccination list according to the present national guidelines, the possibility of blinding opening, the assigned treatment and the possible vaccination will be discussed. In any case the best option for the subject will be pursued*<br>➢ Albumin was included in the Blood Chemistry tests.<br>➢ A few typos were corrected. |
| 3.0 | 22FEB21 | The Clinical Study Protocol, version 2.0, dated 25JAN21, was modified according to a recommendation of the Istituto Superiore di Sanita' (ISS), Italy, as follows:<br>➢ Anti Sars CoV2 serology tests have been added at all time-points foreseen for safety laboratory analysis from Visit 3 to Visit 12 or Early Termination Visit. The rationale is to exclude from the pharmacokinetic analysis any possible asymptomatic infection subjects.<br><br>In addition it has been added that off-site monitoring activities (e.g. phone calls, video visits, emails or other remote tools in order to discuss the trial with the investigator and review the data) could be implemented after a suitable risk evaluation, if routine monitoring site visits are not possible during public health crisis for COVID-19 infection.<br>Diastolic blood pressure normal ranges for inclusion in the study were modified. Also, a few typos were corrected. |

| | | |
|---|---|---|
| 4.0 | 28MAY21 | The Clinical Study Protocol version 3.0, dated 22FEB21, was modified to clarify that from Visit 3 to Visit 12/ETV, only anti-N_SARS-CoV-2 (and not also anti-S) serology test will be performed as scheduled.<br>In addition the following changes were made:<br>➢ In the previous CSP versions it was erroneously stated that solicited AEs (reported in diary 1 by the subjects) were to be considered treatment-related by definition. In the present version the statement was deleted. In fact, final judgment on treatment-relationship is performed by the Investigator as clearly specified in sections 10.4.1 and 11.5 of the clinical study protocol (all versions).<br>➢ Screening number and subject identification number were modified according to the numbers entered in the eCRF and source documents.<br>➢ An additional interim analysis on serum samples for pharmacokinetics evaluation up to day 30 will be performed under blind conditions.<br>➢ For the PK interim analyses no PK parameters will be calculated, only concentrations will be listed.<br>➢ An interim analysis on serum neutralising power data for all subjects up to day 8 and up to day 30 will be performed under blind conditions<br>➢ An interim analysis on serum samples for ELISA Diesse kit characterization up to day 8 will be performed under blind conditions<br>➢ The previously planned interim analysis on ADA data up day 8 will not be performed<br>➢ Chiara Lottici is the new study Clinical Project Leader<br>➢ A few typos were corrected |
| 5.0 | 09JUL21 | In this amended protocol the following changes were introduced:<br>➢ One saliva sample will be collected from each patient at one time point for additional analyses. The analyses will be performed at Vismederi S.p.A., Italy<br>➢ A few typos were corrected |

# PROTOCOL APPROVAL

**SPONSOR**
Toscana Life Sciences Sviluppo s.r.l, Siena, Italy

**Sponsor representatives**

Andrea Paolini, Direttore Generale

| | |
|---|---|
| Date | Signature |

Sarah Nosari, Senior Program Officer

| | |
|---|---|
| Date | Signature |

**INVESTIGATORS**

**Principal investigator - Clinical Centre N. 1**

*I have read this protocol and agree to conduct this study in accordance with all the stipulations of the protocol and in accordance with the Declaration of Helsinki, the current revision of Good Clinical Practice (GCP), ICH topic E6 (R2), and the applicable local law requirements, including supervising any individual or party to whom I will delegate trial-related duties and functions at the trial site.*

Simone Lanini, MD
Istituto Nazionale Malattie Infettive Lazzaro Spallanzani, Rome, Italy

| Date | Signature |
|---|---|

**Principal investigator - Clinical Centre N. 2**

*I have read this protocol and agree to conduct this study in accordance with all the stipulations of the protocol and in accordance with the Declaration of Helsinki, the current revision of Good Clinical Practice (GCP), ICH topic E6 (R2), and the applicable local law requirements, including supervising any individual or party to whom I will delegate trial-related duties and functions at the trial site.*

Stefano Milleri, MD
Centro Ricerche Cliniche di Verona s.r.l. (CRC), Verona, Italy

| Date | Signature |
|---|---|

**CRO**
CROSS Research S.A., Switzerland

**Coordination**
Chiara Lottici, Clinical Project Leader

| Date | Signature |
|---|---|

**Clinical Projects Unit and Medical Writing Team Representative**
Chiara Leuratti, Clinical Projects Unit Head

| Date | Signature |
|---|---|

**Pharmacokinetic Expert**
Andrea Di Stefano, Senior Medical Writer

| Date | Signature |
|---|---|

**Biometry Unit Representative**
Alessandra Gentili, Biometry Manager, Unit Head

| Date | Signature |
|---|---|

**Quality Assurance Unit Representative**
Mario Corrado, Quality Assurance Manager, Unit Head

| Date | Signature |
|---|---|

## STUDY SYNOPSIS

**Title:** COVID-19: A Phase I dose-escalation study to evaluate the safety and pharmacokinetics of anti-SARS-CoV-2 monoclonal antibody MAD0004J08 in healthy adults

**Protocol number:** CRO-20-144 / Sponsor code A0001A / EudraCT N. 2020-005469-15

**Clinical phase:** Phase I

**Study design:** First-in-human, single-dose, dose-escalation, double-blind, placebo-controlled, randomised, safety and pharmacokinetics study

**Planned nr. of centres / countries:** Two/Italy

**Investigators and clinical centres:**
Centre N. 1: Simone Lanini, MD; Istituto Nazionale Malattie Infettive Lazzaro Spallanzani, via Portuense 292, Rome, Italy (coordinating site)
Centre N. 2: Stefano Milleri, MD; Centro Ricerche Cliniche di Verona s.r.l. (CRC), c/o Policlinico G. B. Rossi, p.le Scuro 10, Verona, Italy

**Investigational products:**
TEST: MAD0004J08 100 mg, 2.5 mL solution for injection, Toscana Life Sciences Sviluppo (TLS-S), Siena, Italy
PLACEBO: Matching placebo, 2.5 mL solution for injection, TLS-S, Siena, Italy

**Dose regimen:**
Three single ascending doses (48 mg, 100 mg and 400 mg, abbreviated as D1, D2 and D3) and placebo (P) will be administered by intramuscular (i.m.) injection to three study cohorts (10 subjects/cohort) according to the following scheme:

| | **Cohort 1 - 48 mg** | | **Cohort 2 - 100 mg** | | **Cohort 3 - 400 mg** | |
|---|---|---|---|---|---|---|
| | **Group 1** | **Group 2** | **Group 3** | **Group 4** | **Group 5** | **Group 6** |
| P | 1* | 1 | 1* | 1 | 1* | 1 |
| D1 (48 mg) | 4* | 4 | - | - | - | - |
| D2 (100 mg) | - | - | 4* | 4 | - | - |
| D3 (400 mg) | - | - | - | - | 4* | 4 |
| Tot/group | 5 | 5 | 5 | 5 | 5 | 5 |
| Tot/cohort | 10 | | 10 | | 10 | |

*Sentinel subjects will be treated one at the time at 48 h distance (after evaluation of any possible treatment-related adverse event)
Active treatment (D1/D2/D3) and placebo (P) will be assigned within each cohort and group according to the study randomisation list. Study products will be administered as single doses, by i.m. injection, in the morning of day 1.
Go/No-go decision on escalation from Cohort 1 to Cohort 2 and from Cohort 2 to Cohort 3 will be taken after evaluation of Cohort 1/Cohort 2 safety data up to 48 h post-dose by an independent Data Safety Monitoring Board (DSMB).

For the single dose administrations, the following procedures will be followed:
Cohort 1:
- D1 (48 mg) solution for injection will be prepared by mixing 1.2 mL from one vial of Test product with 2.5 mL from one vial of Placebo and 1.3 mL from another one vial of Placebo (total 3.8 mL Placebo solution), for a total volume of 5 mL.
- P solution for injection will be prepared by mixing 1.2 mL from one vial of Placebo with 2.5 mL from another vial of Placebo and 1.3 mL from a third vial of Placebo, for a total volume of 5 mL (to maintain the blinding).

D1 or P solution (5 mL) will be administered by i.m. injection into the right gluteus (one injection/treatment only).
Cohort 2:
- D2 (100 mg) solution for injection will be prepared by mixing 1 vial of Test product and 1 vial of Placebo for a total volume of 5 mL.
- P solution for injection will be prepared by mixing 2 vials of Placebo for a total volume of 5 mL.

D2 or P solution (5 mL) will be administered by i.m. injection into the right gluteus (one injection/treatment only).

# STUDY SYNOPSIS (cont.)

**Dose regimen, continued:**
Cohort 3:
- D3 (400 mg) will be prepared as 2 solutions for injection, both obtained by mixing 2 vials of Test product [volume for each injection 5 mL (i.e. 200 mg/injection) for a total injected volume of 10 mL].
- P will also be prepared as 2 separate solutions for injection, both obtained by mixing 2 vials of Placebo (volume for each injection 5 mL, for a total injected volume of 10 mL).

For both D3 and P, two i.m. injections (5 mL each) / treatment will be performed for a total volume injected of 10 mL for each subject. For each subject, 5 mL will be injected into the right gluteus and the other 5 mL will be injected into the left gluteus.

**Objectives:** The primary objective of the study is to evaluate the safety of three single dose levels of MAD0004J08 in healthy subjects.
Secondary objectives are to evaluate the pharmacokinetics of MAD0004J08, potency in terms of serum neutralisation power and immunogenicity in terms of generation of anti-drug antibodies (ADAs) after single dose of 48 mg, 100 mg and 400 mg.

**End-points:**
**Primary end-points/variables:** Proportion of subjects with severe / serious treatment-emergent adverse events (TEAEs) (including clinically relevant laboratory abnormalities, vital signs and adverse reactions at the injection site) in the 7 days post-treatment.
**Secondary end-points/variables:**
- Proportion of subjects with any unsolicited and solicited TEAE (including clinically relevant laboratory abnormalities, vital signs and adverse reactions at the injection site) up to each assessment time and throughout the study
- MAD0004J08 serum concentrations and pharmacokinetic parameters $C_{max}$, $t_{max}$, $AUC_{0-t}$, $AUC_{0-\infty}$, $t_{1/2}$, $Cl_t/F$, $V_z/F$ after single dose of 48 mg, 100 mg and 400 mg
- Number and percentage of ADA positive subjects and mean maximum ADA concentration after single dose of 48 mg, 100 mg and 400 mg MAD0004J08
- Serum neutralising power at baseline, 48 h post-dose, on day 8, at 1 month (day 30±3), 4 months (day 120±4) and 6 months (180±7)

**Exploratory end-points/variables:**
- Characterisation of DIESSE ELISA kit on serum samples at baseline, 48 h post-dose, on day 8, at 1 month (day 30±3), 4 months (day 120±4) and 6 months (day 180±7)
- IgG analysis and neutralising power in saliva samples at 6 months (day 180±7)

**Safety and tolerability assessments:** Treatment-emergent adverse events (TEAEs) (unsolicited and solicited); vital signs (blood pressure, heart rate, body temperature), safety laboratory parameters, 12-lead electrocardiograms, injection site reactions and physical examinations including body weight. Assessment times are listed in the "Clinical Procedures and Assessments" section below and summarised in the table entitled "Study Schedule".

**Blood sampling for PK analysis and bioanalytics**: Blood samples for pharmacokinetics analysis will be collected at pre-dose (0h) and at 1h, 2h, 3h, 4h, 6h, 8h, 12h, 24h, 48h post-dose, on day 8, day 15 (±2), day 22 (±2), at 1 month (day 30±3), 2 months (day 60±4), 4 months (day 120±4) and 6 months (day 180±7).
Serum MAD0004J08 concentrations will be determined at Ardena Bioanalysis B.V. (The Netherlands) using a validated ELISA method.

**Blood sampling for ADA analysis**: Blood samples for ADA analysis will be collected at pre-dose (0h) and on day 8, day 15 (±2), at 1 month (day 30±3), 4 months (day 120±4) and 6 months (day 180±7). Serum ADA concentrations will be measured at Ardena Bioanalysis B.V. (The Netherlands) using a validated ELISA method.

**Blood sampling for serum neutralising power test**: Blood samples for serum neutralising power test will be collected at pre-dose (0h), 48 h post-dose, on day 8, at 1 month (day 30±3), 4 months (day 120±4) and 6 months (day 180±7). The test will be performed at Vismederi s.r.l., Italy, or at Laboratorio di Virologia, Istituto Nazionale per le Malattie Infettive Lazzaro Spallanzani, IRCCS, Italy.

**Blood sampling for DIESSE ELISA kit characterisation**: Blood samples for DIESSE ELISA kit characterisation will be collected at pre-dose (0h), 48 h post-dose, on day 8, at 1 month (day 30±3), 4 months (day 120±4) and 6 months (day 180±7). The test will be performed at Toscana Life Sciences, Italy.

**Saliva sampling for additional analyses:**
Saliva samples for IgG analysis and neutralising power test will be collected at 6 months (day 180±7). The analyses will be performed at Vismederi s.r.l., Italy.

## STUDY SYNOPSIS (cont.)

**Sample size:** A total of 30 healthy men and women (10 in each cohort) will be enrolled in the study.

**Inclusion criteria:**

1. *Informed consent*: Signed written informed consent before inclusion in the study
2. *Full comprehension*: Ability to comprehend the full nature and purpose of the study, including possible risks and side effects; ability to co-operate with the Investigator and to comply with the requirements of the entire study
3. *Sex and age*: Healthy men and women, 18 - 55 years old, inclusive
4. *Negative SARS-CoV-2* serology test at screening (negative anti-S and anti-N)
5. *Negative SARS-CoV-2* qRT-PCR in the 72 h before treatment (test on day -3 or -2 or -1 with result before treatment)
6. *Body Mass Index*: 18.5-30 kg/m$^2$, inclusive, at screening
7. *Vital signs*: Systolic blood pressure 90-139 mmHg, diastolic blood pressure 60-90 mmHg, heart rate 50-100 bpm, measured after 5 min at rest in the supine position
8. *ECG*: Electrocardiogram without clinically significant abnormalities at screening
9. *Contraception and fertility*: Women of child-bearing potential must be using at least one of the following reliable methods of contraception and confirm to use adequate contraception during the study:
   a. Hormonal oral or implantable or transdermal, or injectable contraceptives for at least 2 months before the screening visit;
   b. A non-hormonal intrauterine device or female condom with spermicide or contraceptive sponge with spermicide or diaphragm with spermicide or cervical cap with spermicide for at least 2 months before the screening visit
   c. A male sexual partner who agrees to use a male condom with spermicide
   d. A sterile sexual partner
   e. A same sex partner

   Female participants of non-child-bearing potential or in post-menopausal status for at least 1 year will be admitted. For all women, urine pregnancy test result must be negative at screening and day 1

**Exclusion criteria:**

1. *Physical findings*: Clinically significant abnormal physical findings which could interfere with the objectives of the study
2. *Allergy*: Ascertained or presumptive hypersensitivity to the active principle and/or ingredients of the investigational products; history of anaphylaxis to drugs or allergic reactions likely to be exacerbated by any component of the investigational products in the Investigator's opinion
3. *Concomitant medications*: Medications, including over the counter (OTC) medications and herbal remedies, for 2 weeks before screening and immunoglobulin or blood products for 6 months before screening (except contraceptives or a single use of paracetamol, aspirin, or combination OTC products containing paracetamol with an antihistamine, or OTC non-steroidal anti-inflammatory drugs (NSAIDs) at a dose equal or lower than that recommended on the package; vitamins and nutritional supplements, if regularly taken before the study, are also allowed)
4. *Monoclonal Antibodies (mAb)*: Previous intake of a mAb within 6 months, or 5 antibody half-life, whichever is longer, before study start
5. *Transient acute illness:* Acute (time-limited) illness, including fever above 37.5°C on the day before or on the day of the planned treatment; subjects excluded for transient acute illness may be dosed if illness resolves within the screening period or may be rescreened once
6. *Diseases*: Significant history of renal, hepatic, gastrointestinal, cardiovascular, respiratory, skin, haematological, endocrine, psychiatric or neurological diseases that may interfere with the aim of the study or increase subjects risks; history of malignancy in the last 5 years
7. *SARS CoV-2 or COVID-19*:
   *a.* Participants with any confirmed current or previous COVID-19 infection at screening, or at day -1 or day 1
   *b.* Participant with clinical signs or symptoms consistent with COVID-19, e.g. fever, dry cough, dyspnoea, sore throat, fatigue or confirmed infection by appropriate laboratory test within the last 4 weeks before/at screening or at day -1 or day 1
   *c.* Any prior intake of investigational or licensed vaccine indicated for the prevention of SARS CoV-2 or COVID-19 or expected intake during follow-up period
   *d.* Has been reported as a case (confirmed or probable) of COVID-19 from the regional health system

## STUDY SYNOPSIS (cont.)

**Exclusion criteria, continued:**

8. *Immunodeficiency* due to illness, including HIV infection (positivity to anti-HIV-Ab), or due to drugs, including any course of glucocorticoid therapy exceeding 2 weeks of prednisone or equivalent within 6 months before screening.
9. *Infections:* History of active infection with hepatitis B or C or positive test result for anti-HCV-Ab or HBsAg or anti-HBc-Ab at screening; history of infection with SARS or MERS
10. *Laboratory analyses*: Abnormal laboratory values that in the opinion of the Investigator are clinically significant
11. *Investigative drug studies*: Participation in the evaluation of any investigational product for 6 months before this study
12. *Blood donation*: blood donations for 3 months before the study, during the study and in the 3 months after the end of the study
13. *Drug test*: positive drug test at screening or day -1
14. *Drug, alcohol*: history of drug or alcohol abuse within 6 months before screening
15. *Pregnancy (women only)*: positive or missing pregnancy test at screening or day 1; pregnant or lactating women
16. *Other:* Any condition that might compromise study subject's safety or interfere with the study evaluations or interpretation of subject's safety or study results

***IMPORTANT NOTE 1:*** *Subjects screened and kept as reserve or not enrolled for any transient reason can be rescreened once for the study, according to the Investigator's opinion*

***IMPORTANT NOTE 2:*** *In case, during the study, a subject is included in a vaccination list according to the present national guidelines, the possibility of blinding opening, the assigned treatment and the possible vaccination will be discussed. In any case the best option for the subject will be pursued*

**Clinical procedures and assessments:**

The following clinical procedures and assessments will be performed for both Cohorts 1 and 2:

| Visit / Day | Procedures/Assessments | Notes |
|---|---|---|
| **Visit 1 - Screening Day -21 / Day -2** | ➢ Explanation to the subject of study aims, procedures and possible risks<br>➢ Informed consent signature<br>➢ Screening number assignment (as S001, S002, etc.)<br>➢ Demographic data and life style recording<br>➢ Medical history<br>➢ Previous/concomitant medications<br>➢ Physical examination (including body weight, height)<br>➢ Vital signs (blood pressure, heart rate, body temperature)<br>➢ 12-lead ECG<br>➢ SARS-CoV-2 serology test<br>➢ Laboratory analyses: haematology, blood chemistry, urinalysis, serum virology, coagulation, ferritin<br>➢ Drug of abuse test<br>➢ Urinary pregnancy test (women)<br>➢ SARS-CoV-2 qRT-PCR in the 72 h before day 1 (could be performed on day -3 or day-2 or day -1 / see below)<br>➢ Adverse event monitoring<br>➢ Inclusion/exclusion criteria evaluation<br>➢ Eligibility evaluation | Ambulatory visit<br><br>*Note:*<br>*From day 1 (visit 3) to day 8 (Visit 6; diary 1) /month 1 (Visit 9; diary 2), the subjects will daily record in 2 diaries any change in physical or medical conditions and the intake of any concomitant treatment.*<br><br>*The investigator will check the diary at each visit (up to Visit 6 and Visit 9, respectively, for the 2 diaries).* |
| **Visit 2 - Day -1** | ➢ SARS-CoV-2 qRT-PCR in the 72 h before treatment. On day -1 either test performance, or result check if already performed on day -3 or day -2<br>➢ Drug of abuse test<br>➢ Inclusion/exclusion criteria evaluation<br>➢ Check of AEs and concomitant medications<br>➢ Vital signs (blood pressure, heart rate, body temperature) | Arrival at the clinical centre in the evening of day -1.<br>Confinement until the morning of day 2 (see below)<br>Standardised dinner |

## STUDY SYNOPSIS (cont.)

| **Clinical procedures and assessments, continued:** | | |
|---|---|---|
| **Visit / Day** | **Procedures/Assessments** | **Notes** |
| **Visit 2 - Day -1 (cont.)** | ➢ Physical examination<br>➢ Eligibility evaluation | |
| **Visit 3 - Day 1** | ➢ Urine pregnancy test (women)<br>➢ Inclusion/exclusion criteria evaluation, eligibility evaluation, and randomisation<br>➢ 12-lead ECG<br>➢ Dispensation of 2 diaries to the subject (diary 1 to report solicited adverse events from day 1 to day 8, diary 2 to report all unsolicited adverse events and concomitant medication from day 1 to month 1)<br>➢ Laboratory analyses: haematology, coagulation, blood chemistry, urinalysis, SARS-CoV-2 serology test - baseline<br>➢ Blood sample collection for pharmacokinetic analysis at pre-dose (0) and 1h, 2h, 3h, 4h, 6h, 8h, 12h post-dose<br>➢ Blood sample collection for ADA analysis at pre-dose (0)<br>➢ Blood sample collection for serum neutralising power test at pre-dose (0)<br>➢ Blood sample collection for DIESSE Elisa kit characterisation at pre-dose (0)<br>➢ Vital signs (blood pressure, heart rate, body temperature) measurement at pre-dose (0), 2h post-dose and 12 h post-dose<br>➢ Investigational product administration (active or placebo according to study Cohort, Group and randomisation)<br>➢ Adverse events monitoring<br>➢ Injection site reactions check<br>➢ Concomitant medications check | Confinement<br>Standardised meals at the clinical centre |
| **Visit 4 - Day 2** | ➢ Physical examination<br>➢ Vital signs (blood pressure, heart rate, body temperature) measurement at 24 h post-dose<br>➢ Blood sample collection for pharmacokinetic analysis at 24h post-dose<br>➢ Adverse events monitoring<br>➢ Injection site reactions check<br>➢ Diary 1 and diary 2 check<br>➢ Concomitant medications check<br>➢ Laboratory analyses: haematology, blood chemistry, coagulation, urinalysis, SARS-CoV-2 serology test at 24 h post-dose<br>➢ 12-lead ECG at 24 h post-dose | Confinement<br><br>Discharge in the morning of Day 2, after the 24 h post-dose blood sampling. Upon leaving, the subjects will be instructed to contact immediately the Investigator in case of occurrence of any adverse events |
| **Visit 5 - Day 3** | ➢ Vital signs (blood pressure, heart rate, body temperature) measurement at 48 h post-dose<br>➢ Blood sample collection for pharmacokinetic analysis at 48h post-dose<br>➢ Blood sample collection for serum neutralising power test at 48 h post-dose<br>➢ Blood sample collection for DIESSE Elisa kit characterisation at 48 h post-dose<br>➢ Adverse events monitoring<br>➢ Injection site reactions check<br>➢ Diary 1 and diary 2 check<br>➢ Concomitant medications check | Ambulatory visit |

## STUDY SYNOPSIS (cont.)

| Visit / Day | Procedures/Assessments | Notes |
|---|---|---|
| **Visit 5 - Day 3 (cont.)** | ➢ Laboratory analyses: haematology, blood chemistry, coagulation, urinalysis, SARS-CoV-2 serology test - at 48 h post-dose<br>➢ Physical examination | Ambulatory visit |
| **Visits 6 / Week 1 / Day 8**<br><br>**and**<br><br>**Visit 7 / Week 2 / Day 15±2** | ➢ Physical examination<br>➢ Vital signs (blood pressure, heart rate, body temperature) measurement<br>➢ Blood sample collection for pharmacokinetic analysis<br>➢ Blood sample collection for ADA analysis<br>➢ Blood sample collection for serum neutralising power test on day 8 only<br>➢ Blood sample collection for DIESSE Elisa kit characterisation on day 8 only<br>➢ Adverse events monitoring<br>➢ Concomitant medications check<br>➢ Laboratory analyses: haematology, blood chemistry, coagulation, urinalysis, SARS-CoV-2 serology test<br>➢ Diary 1 check - day 8 only<br>➢ Diary 2 check - day 8 and day 15<br>➢ Subjects' diary 1 return - day 8 only | Ambulatory visit |
| **Visit 8**<br>**Week 3**<br>**Day 22±2** | ➢ Physical examination<br>➢ Vital signs (blood pressure, heart rate, body temperature)<br>➢ Blood sample collection for pharmacokinetic analysis<br>➢ Adverse events monitoring<br>➢ Diary 2 check<br>➢ Concomitant medications check<br>➢ Laboratory analyses: haematology, blood chemistry, coagulation, urinalysis, SARS-CoV-2 serology test | Ambulatory visit |
| **Visit 9 - 1 month**<br>**Day 30±3** | ➢ Physical examination<br>➢ Vital signs (blood pressure, heart rate, body temperature) measurement<br>➢ Blood sample collection for pharmacokinetic analysis<br>➢ Blood sample collection for ADA analysis<br>➢ Blood sample collection for serum neutralising power test<br>➢ Blood sample collection for DIESSE Elisa kit characterisation<br>➢ Adverse events monitoring<br>➢ Diary 2 check<br>➢ Concomitant medications check<br>➢ Laboratory analyses: haematology, blood chemistry, coagulation, urinalysis, SARS-CoV-2 serology test<br>➢ Urine pregnancy test (women)<br>➢ Subjects' diary 2 return | Ambulatory visit |
| **Visit 10 - 2 months Day 60±4** | ➢ Physical examination<br>➢ Vital signs (blood pressure, heart rate, body temperature) measurement<br>➢ Blood sample collection for pharmacokinetic analysis<br>➢ Adverse events monitoring<br>➢ Concomitant medications check<br>➢ Laboratory analyses: haematology, blood chemistry, coagulation, urinalysis, SARS-CoV-2 serology test<br>➢ Urine pregnancy test (women) | Ambulatory visit |

## STUDY SYNOPSIS (cont.)

| **Clinical procedures and assessments, continued:** | | |
|---|---|---|
| **Visit / Day** | **Procedures/Assessments** | **Notes** |
| **Visit 11 - 4 months**<br>**Day 120±4 days** | ➢ Physical examination<br>➢ Vital signs (blood pressure, heart rate, body temperature) measurement<br>➢ Blood sample collection for pharmacokinetic analysis<br>➢ Blood sample collection for ADA<br>➢ Blood sample collection for serum neutralising power test<br>➢ Blood sample collection for DIESSE Elisa kit characterisation<br>➢ Adverse events monitoring<br>➢ Concomitant medications check<br>➢ Laboratory analyses: haematology, blood chemistry, coagulation, urinalysis, SARS-CoV-2 serology test<br>➢ Urine pregnancy test (women) | Ambulatory visit |
| **Visit 12 - 6 months**<br>**Final visit**<br>**Day 180±7 days** | ➢ Physical examination (including body weight)<br>➢ Vital signs (blood pressure, heart rate, body temperature) measurement<br>➢ Blood sample collection for pharmacokinetic analysis<br>➢ Blood sample collection for ADA<br>➢ Blood sample collection for serum neutralising power test<br>➢ Blood sample collection for DIESSE Elisa kit characterisation<br>➢ Saliva sample collection for IgG analysis and neutralising power test<br>➢ Adverse events monitoring<br>➢ Concomitant medications check<br>➢ Laboratory analyses: haematology, blood chemistry, urinalysis, coagulation, SARS-CoV-2 serology test<br>➢ 12-lead ECG | Ambulatory visit<br><br>In case of premature discontinuation, the same assessments foreseen as for visit 12 will be performed as far as possible at the time of discontinuation (Early termination visit) |

**Data analysis:**
The data documented in this trial and the measured clinical parameters will be presented using classic descriptive statistics for quantitative variables and frequencies for qualitative variables.
A Statistical Analysis Plan will be prepared by CROSS Research S.A. Biometry Unit, approved by the Sponsor and finalised before database lock.
Safety, serum neutralising power, immunogenicity and demography data will be analysed by CROSS Research Biometry Unit using SAS® version 9.3 (TS1M1) or higher (the actual version will be stated in the final report).
Adverse events and serious adverse events (including clinically significant laboratory parameters, vital signs, ECG results and adverse reactions at the injection sites) will be listed by treatment. Number and percentage of subjects with adverse events and serious adverse events will be summarised.
Serum MAD0004J08 concentrations and pharmacokinetic parameters will be analysed using Phoenix WinNonlin® validated version 6.3 or higher (Pharsight Corporation) and SAS® version 9.3 for Windows® or higher. Pharmacokinetics data will be listed and summarised by descriptive statistics. Individual and mean concentration curves will also be generated.
ADA concentrations will be summarised by descriptive statistics. Number and percentage of ADA-positive subjects will be listed and summarised by assessment time and overall. Serum neutralising power results will be listed by subject and assessment time-point and summarised by descriptive statistics.

**Timing:** Planned clinical phase: February - August 2021

# STUDY SCHEDULE

| ACTIVITIES - Visit | V1 | V2 | V3 | V4 | V5 | V6 | V7 | V8 | V9 | V10 | V11 | V12 Final visit[11] |
|---|---|---|---|---|---|---|---|---|---|---|---|---|
| | Screening | | Intervention | | | Week 1 | Week 2 | Week 3 | Month 1 | Month 2 | Month 4 | Month 6 |
| Day | Day -21/-2 | Day -1 | Day 1 | Day 2 | Day 3 | Day 8 | Day 15±2 | Day 22±2 | Day 30±3 | Day 60±4 | Day 120±4 | Day 180±7 |
| **Informed consent** | x | | | | | | | | | | | |
| **Demography and lifestyle** | x | | | | | | | | | | | |
| **Medical history** | x | | | | | | | | | | | |
| **Physical examination** | x | x | | x | x | x | x | x | x | x | x | x |
| **Body weight** | x | | | | | | | | | | | x |
| **Height** | x | | | | | | | | | | | |
| **Previous and concomitant medications[10]** | x | x | x | x | x | x | x | x | x | x | x | x |
| **Safety Laboratory analysis[13]** | x | | x[1] | x | x | x | x | x | x | x | x | x |
| **Virology and ferritin** | x | | | | | | | | | | | |
| **SARS-CoV-2 serology test** | x | | | | | | | | | | | |
| **SARS-CoV-2 qRT-PCR test** | x[12] | x[12] | | | | | | | | | | |
| **Abuse drug test** | x | x | | | | | | | | | | |
| **Blood pressure, heart rate, body temperature** | x | x | x[2] | x[3] | x[4] | x | x | x | x | x | x | x |
| **12-lead ECG** | x | | x[1] | x[3] | | | | | | | | x |
| **Pregnancy test[5]** | x | | x | | | | | | x | x | x | |
| **Inclusion/exclusion criteria** | x | x | x | | | | | | | | | |
| **Subject eligibility** | x | x | x | | | | | | | | | |
| **Enrolment and randomisation** | | | x | | | | | | | | | |
| **Confinement** | | x[6] | x[6] | | | | | | | | | |
| **Discharge** | | | | x[7] | | | | | | | | |
| **Investigational product administration** | | | x | | | | | | | | | |
| **Blood sampling for PK analysis[8]** | | | x | x | x | x | x | x | x | x | x | x |
| **Blood sampling for ADA analysis[9]** | | | x | | | x | x | | x | | x | x |

| ACTIVITIES - Visit | V1 | V2 | V3 | V4 | V5 | V6 | V7 | V8 | V9 | V10 | V11 | V12 Final visit[11] |
|---|---|---|---|---|---|---|---|---|---|---|---|---|
| | Screening | | Intervention | | | Week 1 | Week 2 | Week 3 | Month 1 | Month 2 | Month 4 | Month 6 |
| Day | Day -21/-2 | Day -1 | Day 1 | Day 2 | Day 3 | Day 8 | Day 15±2 | Day 22±2 | Day 30±3 | Day 60±4 | Day 120±4 | Day 180±7 |
| **Blood sampling for serum neutralising power analysis[14]** | | | x | | x | x | | | x | | x | x |
| **Blood sampling for DIESSE kit characterisation[14]** | | | x | | x | x | | | x | | x | x |
| **Saliva sample collection** | | | | | | | | | | | | x |
| **Adverse event monitoring[10]** | x | x | x | x | x | x | x | x | x | x | x | x |
| **Injection site reactions check** | | | x | x | x | | | | | | | |
| **Subjects' diaries[15]** | | | x[16] | x[17] | x[17] | x[17,18] | x[17] | x[17] | x[17,18] | | | |
| **Interim safety evaluation** | | | | | x | | | | | | | |

1. *At baseline*
2. *At pre-dose (0), 2 h and 12 h post-dose*
3. *At 24 h post-dose*
4. *At 48 h post-dose*
5. *Urine pregnancy test*
6. *Subjects will be resident at the clinical centre from day -1 (Visit 2) to the morning of day 2 (Visit 4).*
7. *After planned safety assessments and pharmacokinetic sampling*
8. *Blood sampling for pharmacokinetic analysis at pre-dose (0h) and 1h, 2h, 3h, 4h, 6h, 8h, 12h, 24h, 48h post-dose, on day 8, day 15±2, day 22±2, at 1 month (day 30±3), 2 months (day 60±4), 4 months (day 120±4)and 6 months (day 180±7)*
9. *Blood samples for ADA analysis at pre-dose (0h) and on day 8, day 15±2, at 1 month (day 30±3), 4 months (day 120±4) and 6 months (day 180±7)*
10. *Monitoring starting at the screening visit, immediately after informed consent, up to the final visit*
11. *In case of premature discontinuation, as far as possible an early termination visit will be performed, including procedures and assessment foreseen for the study final visit*
12. *Within 72h before day 1 treatment (On day- 3, day -2 or day -1). If performed before day -1, check on day -1. In any case check on day 1*
13. *Haematology, blood chemistry, coagulation, urine analysis. Also virology/SARS-CoV2 serology test (anti-N) from Visit 3 to Visit 12/ETV*

14. *Blood samples for serum neutralising power test and for DIESSE Elisa kit characterisation at pre-dose (0h) and 48 h post-dose, on day 8, at 1 month (day 30±3), 4 months (day 120±4) and 6 months (180±7) post-dose*
15. *Subjects will report solicited local and systemic AE on diary 1 and unsolicited and concomitant medication on diary 2*
16. *Diary 1 and diary 2 dispensed to subjects*
17. *Diary check by investigator or deputy and recording of any adverse event and concomitant medication in the eCRF*
18. *Diary return to clinical centre (return of diary 1 at Visit 6, return of diary 2 at Visit 9)*

# TABLE OF CONTENTS

Page

**CLINICAL STUDY PROTOCOL** **1**
**VERSIONS' HISTORY** **2**
**PROTOCOL APPROVAL** **4**
**STUDY SYNOPSIS** **8**
**STUDY SCHEDULE** **15**
**TABLE OF CONTENTS** **18**
**TABLES** **21**
**FIGURES** **21**
**1 Introduction** **24**
1.1 Background 24
1.2 Anti-SARS-CoV-2 monoclonal antibody MAD0004J08 24
1.2.1 In vitro analysis for MAD0004J08 characterisation 25
1.2.2 Autoreactivity evaluation of MAD0004J08 to human epithelial cells 25
1.2.3 MAD0004J08 Fc engineering to eliminate risk of ADE and extend antibody half-life 26
1.3 In vivo study to assess MAD0004J08 prophylactic activity in a golden Syrian hamster SARS-CoV-2 infection model 26
*1.4 Dose calculation* 27
1.5 Rationale 28
1.6 Risks and benefits 29
**2 STUDY OBJECTIVES** **31**
2.1 Primary end-point 31
2.2 Secondary end-points 31
2.3 Exploratory end-point 31
**3 CLINICAL SUPPLIES** **32**
3.1 Treatment 32
3.1.1 Description of products 32
3.1.1.1 Test product 32
3.1.1.2 Reference product 32
3.1.2 Dose regimen 32
3.1.3 Route and method of administration 33
3.1.4 Investigational product distribution 34
3.2 Packaging and labelling 34
3.3 Storage conditions 35
3.4 Drug accountability 35
**4 INVESTIGATIONAL PLAN** **36**
4.1 Overall study design 36
4.2 Discussion of design 36
**5 STUDY POPULATION** **39**
5.1 Target population 39
5.2 Inclusion criteria 39
5.3 Exclusion criteria 40
5.3.1 Re-screening 41
5.3.2 Not allowed treatments 41
**6 STUDY SCHEDULE** **42**
6.1 Study visits and procedures 42
6.2 Restrictions, diet and lifestyle 46
**7 DESCRIPTION OF SPECIFIC PROCEDURES** **47**
7.1 Physical examination 47
7.1.1 Body weight, height and BMI 47
7.1.2 Vital signs 47
7.1.3 ECGs 47
7.2 Clinical laboratory assays 48

7.3 Treatment-emergent adverse events 50
7.3.1 Subject's diaries for reporting solicited and unsolicited adverse events 50
7.3.2 Recording and severity grading of solicited AEs 50
7.3.3 Unsolicited AEs 52
7.4 Safety data evaluation at 48 h post-dose 52
7.5 Sampling for pharmacokinetic, ADA, serum neutralising power, Diesse kit and saliva samples analyses 53
7.5.1 Venous blood sampling 53
7.5.1.1 Sample preparation for PK and ADA analysis 54
7.5.1.2 Sample preparation for serum neutralising power test 54
7.5.1.3 Sample preparation for DIESSE kits characterisation 54
7.5.2 Saliva sampling 54
7.5.3 Analytics - PK and ADA 54
7.5.4 Analytics - neutralisation test 55
7.5.5 Analytics - DIESSE Elisa kit characterisation 55
7.5.6 Analytics - Saliva samples analysis 55
7.5.7 Labelling, storage and transport of samples 55
7.5.7.1 Samples labelling 55
7.5.7.2 Samples storage and transport 56
7.6 Total number of blood samples and blood withdrawn 57
**8 ASSIGNMENT OF STUDY TREATMENT 58**
8.1 Randomisation 58
8.2 Treatment allocation 58
8.3 Blinding 58
8.3.1 Emergency code and unblinding procedures 58
**9 EVALUATION PARAMETERS 59**
9.1 Study variables 59
9.1.1 Primary variables 59
9.1.2 Secondary variables 59
9.1.3 Exploratory variable 59
9.2 Pharmacokinetic and ADA assessments 59
9.2.1 Pharmacokinetic parameters 59
9.3 Safety assessments 60
**10 STATISTICAL METHODS 61**
10.1 Analysis Sets 61
10.1.1 Definitions 61
10.1.2 Reasons for exclusion from the PK or ADA set or Serum Neutralising power set or saliva analysis 62
10.2 Sample size and power considerations 62
10.3 Demographic, baseline and background characteristics 62
10.4 Analysis of safety parameters 62
10.4.1 Safety and tolerability evaluation 63
10.4.2 Primary safety analysis 64
10.4.3 Secondary safety analyses 64
10.5 Analysis of pharmacokinetic parameters 64
10.6 Analysis of ADA 64
10.7 Interim analysis of PK data 65
10.8 Serum neutralising power test results 65
10.9 DIESSE Elisa kit characterisation 65
10.10 Saliva samples' analysis results 65
**11 DEFINITION AND HANDLING OF AEs AND SAEs 66**
11.1 Applicable SOPs 66
11.2 Definitions 66
11.3 AEs monitoring window 67
11.4 AEs recording 68
11.5 Guidance for assessing relationship of AE with investigational product 70
11.6 SAEs reporting 71
11.7 SUSARs management 71

| | | |
|---|---|---|
| 11.8 | Other events qualified for expedited reporting | 72 |
| 11.9 | Pregnancy | 72 |
| 11.10 | SAEs: contacts | 72 |
| **12** | **DATA MANAGEMENT PROCEDURES** | **74** |
| 12.1 | Data collection – eCRFs | 74 |
| 12.2 | Unique subject identifier | 74 |
| 12.3 | Database management | 74 |
| 12.3.1 | SDTM and ADaM | 75 |
| 12.3.2 | Coding dictionaries | 75 |
| **13** | **STUDY MONITORING, QUALITY CONTROL AND QUALITY ASSURANCE** | **76** |
| 13.1 | Monitoring | 76 |
| 13.2 | Quality Control and Quality Assurance | 76 |
| 13.3 | Applicable SOPs | 77 |
| 13.4 | Data access | 77 |
| 13.5 | Audits and inspections | 77 |
| **14** | **ETHICAL CONSIDERATIONS** | **78** |
| 14.1 | Ethics and Good Clinical Practice (GCP) | 78 |
| 14.2 | Informed consent | 78 |
| 14.3 | Insurance policy | 79 |
| 14.4 | Withdrawal of subjects | 79 |
| 14.4.1 | Discontinuation procedures | 79 |
| 14.5 | Primary reason for subject's discontinuation | 79 |
| 14.6 | Study termination | 80 |
| 14.7 | Holding rules | 80 |
| **15** | **ADMINISTRATIVE PROCEDURES** | **82** |
| 15.1 | Material supplied to the clinical centre | 82 |
| 15.2 | Protocol amendments | 82 |
| 15.3 | Study documentation and record keeping | 82 |
| 15.4 | Study subjects' recruitment | 83 |
| 15.5 | Confidentiality and data protection | 83 |
| 15.6 | Publication policy | 84 |
| **16** | **STUDY RESPONSIBLE PERSONS** | **85** |
| 16.1 | Sponsor | 85 |
| 16.2 | Data Safety Monitoring Board | 85 |
| 16.3 | Institutes performing the study | 86 |
| 16.3.1 | Clinical centre N. 1 - Coordinating site | 86 |
| 16.3.2 | Clinical centre N 2 | 86 |
| 16.4 | Pharmacokinetics and ADA assays | 86 |
| 16.5 | Serum neutralising power test | 87 |
| 16.6 | DIESSE kit characterisation | 87 |
| 16.7 | Saliva IgG and neutralising power test | 87 |
| 16.8 | Co-ordination, data analysis & reporting | 87 |
| 16.9 | Monitoring | 88 |
| 16.9.1 | Clinical site N. 1 | 88 |
| 16.9.2 | Clinical site N. 2 | 88 |
| 16.10 | Pharmacovigilance | 88 |
| 16.11 | eCRF and IWRS | 89 |
| **17** | **REFERENCES** | **90** |

## TABLES

Page

| | | |
|---|---|---|
| Table 3.1.2.1 | Dose regimen scheme | 32 |
| Table 3.1.3.1 | Administered volumes scheme | 34 |
| Table 6.1.1 | Clinical Procedures and assessments - summary | 43 |
| Table 7.2.1 | Total volume of blood for safety laboratory tests and serology | 49 |
| Table 7.3.1 | Solicited local AE grading scale | 51 |
| Table 7.3.2 | Solicited systemic AE grading scale | 51 |
| Table 7.5.1.1 | Tolerance ranges for the scheduled sampling times | 53 |

## FIGURES

| | | |
|---|---|---|
| Figure 4.2.1 | Enrolment and safety evaluation scheme | 37 |

# LIST OF ABBREVIATIONS

| | |
|---|---|
| γ-GT | γ-Glutamyl transpeptidase |
| ADA | Anti-drug Antibodies |
| ADE | Antibody-dependent enhancement |
| ADNK | Antibody-dependent NK cell activation |
| ADNP | Antibody-dependent neutrophil phagocytosis |
| ADR | Adverse Drug Reaction |
| AE | Adverse Event |
| ALT | Alanine aminotransferase |
| aPTT | activated Partial Thromboplastin Time |
| AST | Aspartate aminotransferase |
| $AUC_{0-t}$ | Area under the concentration-time curve from time zero to time t |
| $AUC_{0-\infty}$ | Area under the concentration vs. time curve up to infinity |
| BLQL | Below Lower Quantification Limit |
| BMI | Body Mass Index |
| BP | Blood Pressure |
| CA | Competent Authority |
| CDISC | Clinical Data Interchange Standards Consortium |
| $CL_t/F$ | Total body clearance |
| $C_{max}$ | Peak drug concentration |
| CRA | Clinical Research Associate |
| eCRF | electronic Case Report Form |
| CRO | Contract Research Organisation |
| CSP | Clinical Study Protocol |
| CSR | Clinical Study Report |
| CS | Clinically Significant |
| CV | Coefficient of Variation |
| D1 | Dose 1: 100 mg |
| D2 | Dose 2: 400 mg |
| DBP | Diastolic Blood Pressure |
| DSMB | Data Safety Monitoring Board |
| EC | Ethics Committee |
| ECG | Electrocardiogram |
| ETV | Early Termination Visit |
| FSFV | First Subject First Visit |
| GCP | Good Clinical Practice |
| GLP | Good Laboratory Practice |
| HBs Ag | Hepatitis B virus surface antigen |
| HCV Ab | Hepatitis C virus antibodies |
| HIV | Human Immunodeficiency Virus |
| HR | Heart Rate |
| IB | Investigator's Brochure |
| IC50 | Half maximal inhibitory concentration |
| IC100 | Maximal inhibitory concentration |
| ICH | International Conference on Harmonisation |
| IgG | Immunoglobulin G |
| INR | International Normalised ratio |
| IRB/IEC | Institutional Review Board/Independent Ethics Committee |
| i.m. | Intramuscular |
| IMP | Investigational Medicinal Product |
| IUD | Intra-Uterine Device |
| IV | Intravenous |
| LSLV | Last Subject Last Visit |
| mAb | Monoclonal Antibody |
| MCH | Mean Cell Haemoglobin |
| MCHC | Mean Cell Haemoglobin Concentration |

| | |
|---|---|
| MCV | Mean Cell Volume |
| MedDRA | Medical Dictionary for Regulatory Activities |
| MRT | Mean Residence Time |
| MW | Molecular Weight |
| N | Normal |
| NA | Not Applicable |
| nAb | Neutralising Antibody |
| NC | Not calculated |
| NCS | Not clinically significant |
| NSAIDs | Non-Steroidal Anti-Inflammatory Drugs |
| OTC | Over The Counter |
| P | Placebo |
| PCR | Polymerase Chain Reaction |
| PK | Pharmacokinetics |
| PT | Preferred Term |
| PTAE | Pre-Treatment Adverse Event |
| R | Reference |
| SAE | Serious Adverse Event |
| SBP | Systolic Blood Pressure |
| SD | Standard Deviation |
| SOC | System Organ Class |
| SOP | Standard Operating Procedure |
| SDTM | Study Data Tabulation Model |
| SUSAR | Suspected Unexpected Serious Adverse Reaction |
| T | Test |
| TEAE | Treatment-Emergent Adverse Event |
| $t_{1/2}$ | Half-life |
| $t_{max}$ | Time to achieve $C_{max}$ |
| USDA | United States Department of Agriculture |
| $V_z/F$ | Distribution volume |
| WBC | White Blood Cells |
| WHODDE | World Health Organisation Drug Dictionary Enhanced |
| WT | Wild type |

# 1 INTRODUCTION

## 1.1 Background

COVID-19 disease can be unpredictable in its severity. The clinical presentation of SARS-CoV-2 infection ranges from asymptomatic to life threatening with multi-organ failure (1).
All subjects infected with SARS-CoV-2, including those with no or mild symptoms, are quarantined until their capability to transmit infection can be ruled out, which may require several weeks (2, 3).

An intervention capable of significantly reducing the time to eradication of infection would be of benefit for affected individuals and society. The benefit would be clearly enhanced if the same intervention could improve clinical symptoms of COVID-19 and prevent and/or shorten hospitalisation.

Human monoclonal antibodies (mAbs) have shown potential in the fight against infectious diseases (4). Examples are mAbs against respiratory syncytial virus (RSV), which have recently shown therapeutic effect in infants with one intramuscular (i.m.) injection of 50 mg (5), human immunodeficiency virus (HIV), where some mAbs have demonstrated broad neutralisation to several clinical strains as well as high virologic suppression (6, 7), and Ebola virus, where mAb therapy was the only effective tool to tackle the dramatic disease caused by this virus (8).

Since the start of the COVID-19 pandemic, several human mAbs capable of neutralising the SARS-CoV-2 virus *in vitro* and to prevent/treat the infection *in vivo* animal models have been identified and some are currently under clinical development (9, 10). Interim clinical results of a Phase I/II study testing Eli Lilly’s intravenous (IV) mAb LY-CoV555 was recently published, suggesting that a single dose from 700 mg to 7000 mg given as an IV bolus may reduce viral load and hospitalisation rate in patients with mild to moderate COVID-19 (11). Interim results from a Phase I/II trial testing Regeneron’s mAb REGN-COV2 disclosed in a press release from the company also suggest reduced viral levels and improved symptoms in non-hospitalised COVID-19 patients (12). It must be emphasised that for REGN-COV2, publication of these clinical data in a peer reviewed journal is pending.

Given the *in vitro* potency and half maximal inhibitory concentration ($IC_{50}$) against SARS-CoV-2 of REGN-COV2 (0.4 – 2 µg/mL) and LY-cov555 (0.02 – 0.05 µg/mL), these antibodies were given to patients at concentrations as high as 8 and 7 grams respectively. These doses made IV administration the only possible route of intervention.

## 1.2 Anti-SARS-CoV-2 monoclonal antibody MAD0004J08

A novel anti-SARS-CoV-2 monoclonal antibody, namely MAD0004J08, has been recently developed by TLS-S, Italy, as preventive and therapeutic tool for SARS-CoV-2 (13, 14).

This monoclonal antibody has shown very high neutralisation potency *in vitro* and completely neutralises the SARS-CoV-2 virus (IC100) at a concentration as low as 0.003 µg/mL. The high potency of MAD0004J08 makes it suitable for i.m. administration.

Furthermore, the constant portion (Fc) of MAD0004J08 was engineered to extend the half-life and to abrogate the Fc binding to cellular receptors, which has been associated to antibody dependent enhancement (ADE) disease (13, 14).

Non clinical studies for MAD0004J08 *in vitro* characterisation, autoreactivity evaluation, and Fc engineering to abrogate Fc binding to cellular receptors and also to extend half-life are summarised hereafter. In addition, results of an *in vivo* study to assess MAD0004J08 prophylactic activity in an animal model are presented.

### *1.2.1 In vitro analysis for MAD0004J08 characterisation*

Several *in vitro* assays were performed for the antibody MAD004J08 to profile its binding characteristics as well as evaluate its functional activity against the live SARS-CoV-2 virus (13).

Initially an enzyme-linked immunosorbent assay (ELISA) was performed against the trimeric pre-fusion stabilised SARS-CoV-2 spike protein (S-protein) and the two different subunits of the spike monomer named S1 and S2 domains.

MAD0004J08 is able to tightly bind to the trimeric S-protein with a half maximal effective concentration ($EC_{50}$) of 5.8 and an affinity (KD) of 0.03E-11 M.

Binding was also detected when the antibody was tested against the S1 domain with an $EC_{50}$ of 4.8 ng/mL. On the contrary, no signal was detected for binding to the S2-domain.

Following the binding profiling of the antibody, MAD0004J08 was assessed for its neutralisation of binding activity (NoB), which aims to evaluate the abrogation of S-protein/receptor binding, and neutralisation potency against both the WT (SARS-CoV-2/INMI1-Isolate/2020/Italy: MT066156) and the widespread SARS-CoV-2 D614G mutated strain (SARS-CoV-2/human/ITA/INMI4/2020, clade GR, D614G (S): MT527178) (15). MAD0004J08 showed to be able to neutralise the S-protein/receptor binding with an $EC_{50}$ of 78.6 ng/mL and to be extremely neutralising against both viral strains with an $IC_{100}$ of 3.9 and 7.8 ng/mL for the WT and D614G strain respectively.

### *1.2.2 Autoreactivity evaluation of MAD0004J08 to human epithelial cells*

Recent findings showed that COVID-19 patients can develop autoantibodies. In particular, it was reported that around 10% of patients with life-threating COVID-19 pneumonia develop autoantibodies against type I interferons tipping the balance of the infection in favour of the virus (16). Another important aspect is the development of prothrombotic autoantibodies in serum of hospitalised COVID-19 patients. These patients develop an antiphospholipid syndrome where autoantibodies target phospholipids and phospholipid-binding proteins resulting in a potentially life-threatening thrombophilia. Over 50% of the serum samples from COVID-19 patients tested positive for antiphospholipid autoantibodies, highlighting the importance of autoantibodies in SARS-CoV-2 infected patients (17).

To eliminate any possible risk of autoreactivity to human antigens, MAD0004J08 wild type (WT) and mutated (MUT) versions were tested through an indirect immunofluorescent assay against human epithelial type 2 cells which expose clinically relevant proteins to detect autoantibody activities (13). While the positive control showed a low but detectable signal at

1:100 dilution, MAD0004J08 did not show any signal in both its WT and MUT version at a concentration of 100 µg/mL.

### 1.2.3 MAD0004J08 Fc engineering to eliminate risk of ADE and extend antibody half-life

Antibody-dependent enhancement (ADE) disease, has been previously shown to be a potential clinical risk following coronaviruses infection (18). Therefore, to make MAD004J08 suitable for clinical purposes, five different point mutations in the antibody constant region were introduced to eliminate the risk of ADE. The first two point mutations (M428L/N434S) were introduced to enhance antibody half-life (19). The remaining three point mutations (L234A/L235A/P329G) were introduced to reduce antibody dependent functions such as binding to FCγRs and cell-based activities (20).
To confirm the lack of FCγR binding as well as the extended half-life, a beads based Luminex assay was performed (13). Briefly the beads were coated with SARS-CoV-2 S-protein receptor binding domain (RBD). MAD0004J08 was tested at eight point dilutions and the binding detected with FCγR2A and FcRn (Neonatal Fc receptor) at pH6.2 and 7.4. The FCγR2A was selected as it is predominantly expressed on the surface of phagocytic cells (such as monocytes, macrophages and neutrophils) and are associated with phagocytosis of immune complexes and antibody opsonised targets (21). On the other hand, FcRn, which is highly expressed on endothelial cells and circulating monocytes, was selected as it is responsible for the recycling and serum half-life of immunoglobulin G (IgG) in the circulation (22). This latter receptor was shown to possess a tighter binding at lower pH (eg. pH 6.2) compared to physiological pHs (e.g. pH 7.4) (23). The results demonstrate that the binding to the FCγR2A was completely abrogated for the mutated version of MAD0004J08 (MAD000J08-MUT) compared to the respective wild type (WT) version (MAD000J08-WT) and control antibody (CR3022). Furthermore, FC-engineered antibodies showed an increased binding activity to the FcRn at both pH 6.2 and 7.4 compared to their wild type counterpart. Finally, to evaluate the lack of FC-mediated cellular activities by our 3 candidate neutralising Antibodies (nAbs), the antibody-dependent neutrophil phagocytosis (ADNP) and antibody-dependent NK cell activation (ADNK) were evaluated (24-26). For the ADNP assay primary human neutrophils were used to detect the antibody binding to SARS-CoV-2 S-protein RBD coated beads, while ADNK activity was evaluated by using primary human NK cells and detecting the release of the proinflammatory cytokine IFN-γ. Complete abrogation of both ADNP and ADNK was observed for all three FC-engineered candidate nAbs compared to their WT versions and control antibody (CR3022) confirming the lack of FC-mediated cellular activities. Further details are given in the IB addendum (37).

## 1.3 In vivo study to assess MAD0004J08 prophylactic activity in a golden Syrian hamster SARS-CoV-2 infection model

The golden Syrian hamster model has been widely used to assess mAb prophylactic and therapeutic activities against SARS-CoV-2 infection. This model has shown to manifest severe forms of SARS-CoV-2 infection mimicking more closely the clinical disease observed in humans (27-30). We designed a prophylactic study in golden Syrian hamster to evaluate the efficacy of MAD0004J08 in preventing SARS-CoV-2 infection (13). For this study, 30 hamsters were divided into 5 arms (6 animals each). The mAb was administered at 3 different concentrations (4 – 1 – 0.25 mg/kg) via intraperitoneal injection. Placebo and IgG class 1

(IgG1) isotype control groups were included in the study which received a saline solution and an anti-influenza antibody at the concentration of 4 mg/kg respectively. The MAD0004J08 4 mg/kg group and the 1 and 0.25 mg/kg groups were tested in 2 independent experiments. The IgG1 isotype control group was tested in parallel with the MAD0004J08 4 mg/kg group while the placebo is an average of the 2 experiments.

Twenty-four (24) h post-dose of the antibody or saline solution, animals were challenged with 100 µL of SARS-CoV-2 solution ($5x10^5$ PFU) via intranasal distillation. Three (3) hamsters per group were sacrificed at 3 days post-infection while the remaining animals were culled at day 8. Body weight changes were daily evaluated throughout the study. It was observed that MAD000J08 significantly reduced weight loss at all concentrations tested in this study in a dose-response fashion compared to both the placebo and the IgG1 isotype control groups. When MAD0004J08 was administered at 4 mg/kg complete protection from SARS-CoV-2 infection was observed and only a minimal weight loss was noticed one day post viral challenge. All animals quickly recovered at day 3 and hamsters reached their initial weight. From day 4 on hamsters gained weight increasing up to 5% from their initial body weight. A slightly bigger body weight loss was observed 1 day post infection in hamsters that received MAD0004J08 at 1 and 0.25 mg/kg. Anyway hamsters in these groups completely recovered their initial body weight at day 6 and 8 for the 1 and 0.25 mg/kg dosage respectively. Hamsters in the control groups did not recover their initial body weight and at day 8 still show around 5% of weight loss.

To conclude, MAD0004J08 showed to be an extremely potent prophylactic tool and was able to prevent SARS-CoV-2 infection in a golden Syrian hamster model even at concentrations as low as 0.25 mg/kg (around 50 µg/animal). Further details are given in the IB addendum (37).

## *1.4 Dose calculation*

As reported above (§ 1.1), in the past years humAbs have shown great potential in the fight against infectious diseases (4). In particular, two clinical studies from Regeneron (REGN-COV2) and Eli Lilly (LY-CoV555) showed that humAbs are effective tools to treat and reduce hospitalization of SARS-CoV-2 seropositive patients (38, 39).
When the in vitro potency and half maximal inhibitory concentration ($IC_{50}$) of REGNCOV2 (0.4 – 2 µg/mL) and LY-CoV555 (0.02 – 0.05 µg/mL) was compared with that of MAD0004J08 (0.003 µg/mL) it was observed that the latter was up to 1000 times more potent. These data suggest that we could use a lower dosage compared to the 8 and 7 grams/patient used clinically for REGN-COV2 and Ly-CoV555 respectively. The possibility to reduce the amount of antibody needed will also allow us to administrate MAD0004J08 intramuscularly, while REGN-COV2 and LY-CoV555, giving the high dosage needed, can only be administrated intravenously.

At this stage no clinical data have been produced showing the efficacy of humAb in treating SARS-CoV-2 when administered intramuscularly.
However, comparison of in vivo studies among REGN-COV2, Ly-CoV555 and MAD0004J08 further strengths the assumption that our candidate antibody can be used at a low dosage maintaining its efficacy in treating COVID-19 infection. REGN-COV2 and Ly-CoV555 are approximately one hundred and ten times respectively less potent in in vitro neutralization of SARSCoV- 2. The difference in potency is also reflected in vivo, where

REGN-COV2 cocktail and Ly- CoV555 showed to clear the virus from lung tissues when used at 5 and 15 mg/kg respectively (Table 1) [4,5]. We obtained the same results when MAD0004J08 was used at 0.25 mg/kg, therefore 20 and 60 times more potent than REGN-COV2 cocktail and Ly-CoV555 respectively. In vivo data for MAD0004J08 are described in session 2 (2.1 and 2.2) of the Addendum to IB (37).

The doses of 100 mg and 400 mg selected for this clinical study are consistent with the relative in vitro and in vivo potency of MAD0004J08. These doses are between 18 and 20 times lower than the maximum doses already used in humans.

In particular, the 100 mg dose proposed for MAD0004J08 in this study was calculated in order to achieve a neutralising titre in the serum of 1/100. Since most Covid-19 convalescent patients have a neutralising titre ranging from 1/20 to 1/320, it is assumed that a titre exceeding 1/100 will provide sufficient neutralising potency to eliminate the virus from the blood and the lungs. Considering MAD0004J08 neutralization potency of 3 ng/mL (i.e. 3 mg/kg), it was calculated that 21 mg for a person of 70 kg would be necessary to achieve a titre of 1/100. The proposed dose of 100 mg exceeds the titre of 1/100 by 5-fold, while the proposed titre of 400 mg exceeds the titre of 1/100 by 20 fold.

In addition, a similar-MABEL approach will be applied according to the Italian Health Authority (Istituto Superiore della Sanita', Italy) and a starting dose of 48 mg MAD0004J08 will be investigated before escalation to the 100 mg and 400 mg doses, if safety concerns do not arise.

In line with the recommendations of the EMA "Guideline on strategies to identify and mitigate risk for first-in-human and early clinical trials with investigational medicinal product" (31), 5 sentinel subjects (4 receiving the active treatment and 1 receiving placebo) are foreseen in group 1 (Cohort 1), group 3 (Cohort 2) ad group 3 (Cohort 3) and will be treated at 48 h intervals, in order to evaluate possible treatment-related adverse events before treating the other subjects in the group.

## 1.5 Rationale

As reported in the previous paragraphs, anti SARS-CoV-2 MAD0004J08 mAb shows very high neutralisation potency *in vitro* and completely neutralises the SARS-CoV-2 virus (IC100) at a concentration as low as 0.003 µg/mL, i.e. it is up to 1000 times more potent than other SARS-CoV-2 mAbs presently under development (13, 14).

Furthermore, Fc of MAD0004J08 was engineered to extend the half-life and to abrogate the Fc binding to cellular receptors, highly decreasing the risk of ADE disease (13, 14).

The 48 mg, 100 mg and 400 mg doses proposed will allow administering MAD0004J08 solution by i.m., as opposed to IV injection. This route of administration could be a key advantage in emergency scenarios as it will allow giving MAD0004J08 in non-hospital care settings increasing the number of people that can quickly benefit from its foreseen prophylactic and therapeutic effect.

In the present study, 3 single ascending doses of 48 mg, 100 and 400 mg MAD0004J08 will be administered by i.m. injection to healthy subjects in 3 sequential cohorts which include 2 groups each (see details in § 3.1.2) with the aim to assess treatment safety and pharmacokinetic (PK) profile.

The study design was chosen in order to minimise possible adverse reactions in each cohort by exposing 1 sentinel subject at the time in each admittance group within each cohort, with 48 h intervals between the treatment of subsequent sentinel subjects according to the current and applicable guideline for first-in-human studies (31), in order to evaluate the occurrence of possible treatment-related adverse events before proceeding with further subject exposure.
Also, go/no-go decision for dose escalation from Cohort 1 (48 mg) to Cohort 2 (100 mg) and from Cohort 2 (100 mg) to Cohort 3 (400 mg) will be taken only after evaluation by an independent Data Safety Monitoring Board (DSMB) of Cohort 1 safety data up to 48 h post-dose.

## 1.6 Risks and benefits

Human mAbs are one of the greatest scientific and medical breakthroughs of the last decades. MAbs are now effectively used to treat diseases in many therapeutic areas including malignancies, immune-mediated diseases (e.g. rheumatoid arthritis, inflammatory bowel disease), and infectious diseases (32).

Human mAbs have been used in millions of patients with a generally satisfactory safety profile (32). Clinically meaningful risks associated with mAb therapy with direct antiviral activity include allergic reactions including anaphylaxis, development of anti-drug antibodies (ADA), potential induction of ADE. Anaphylaxis is a serious allergic reaction that may rapidly onset after administration of exogenous compounds. Anaphylaxis is rare after mAb administration, and prompt intervention is typically associated with resolution. Production of ADA may be associated with loss of efficacy. As ADAs tend to develop after multiple drug administration the risk of ADA in this study is low. ADE implies that mAb may directly increase virulence and pathogenicity of the target viral agent. ADE has been associated with the ability of low level non-neutralising antibodies to bind viral antigens and dysregulate immune response through Fc-dependent pathways (14).

As mAb MAD0004J08 is highly neutralising even at very low doses and lacks the Fc portions that are a pivotal complement for ADE, the risk of ADE with MAD0004J08 is expected to be very low.

Overall, based on the above considerations and the low selected doses, the risk of clinically meaningful adverse events (AEs) following administration of MAD0004J08 is expected to be low.

No direct benefit to the study participants are foreseen, except for a possible preventive effect for COVID-19.

In the future, the benefits expected from MAD0004J08 are the shortening of the time the patient is infectious and must remain quarantined and a more benign clinical course of the disease. A shorter time to infection eradication would reduce the likelihood of spreading the

infection. A shorter duration of the quarantine would have in many patients an important positive impact on income, family life and psychological wellbeing.

A more benign course of COVID-19 would have medical, social, and economic benefits for individual patients and society.

Overall, the benefit-risk ratio of MAD0004J08 in this Phase I trial is considered favourable.

# 2 STUDY OBJECTIVES

The primary objective of the study is to evaluate the safety of three single dose levels of MAD0004J08 in healthy subjects.

Secondary objectives are to evaluate the pharmacokinetics of MAD0004J08, potency in terms of serum neutralisation power and immunogenicity in terms of generation of anti-drug antibodies (ADAs) after single dose of 48 mg, 100 mg and 400 mg.

## 2.1 Primary end-point

- Proportion of subjects with severe / serious treatment-emergent adverse events (TEAEs) (including clinically relevant laboratory abnormalities, vital signs, and adverse reactions at the injection site) in the 7 days post-treatment.

## 2.2 Secondary end-points

- Proportion of subjects with any unsolicited and solicited TEAE (including clinically relevant laboratory abnormalities, vital signs, and adverse reactions at the injection site) up to each assessment time and throughout the study
- MAD0004J08 serum concentrations and PK parameters $C_{max}$, $t_{max}$, $AUC_{0-t}$, $AUC_{0-\infty}$, $t_{1/2}$, $Cl_t/F$, $V_z/F$ after single dose of 48 mg, 100 mg and 400 mg
- Number and percentage of ADA positive subjects and mean maximum ADA concentration after single dose of 48 mg, 100 mg and 400 mg MAD0004J08
- Serum neutralising power at baseline, 48 h post-dose, on day 8, at 1 month (day 30±3), 4 months (day 120±4) and 6 months (180±7) after single dose of 48 mg, 100 mg and 400 mg MAD0004J08

## 2.3 Exploratory end-point

- Characterisation of DIESSE ELISA kit on serum samples at baseline, 48 h post-dose, on day 8, at 1 month (day 30±3), 4 months (day 120±4) and 6 months (180±7)
- IgG analysis and neutralising power test in saliva samples at 6 months (day 180±7)

# 3 CLINICAL SUPPLIES

## 3.1 Treatment

### *3.1.1 Description of products*

#### *3.1.1.1 Test product*

TEST (T)

| | |
|---|---|
| Investigational medicinal product (IMP) | MAD0004J08, human mAb 100 mg / 2.5 mL solution for injection |
| Manufacturer (active substance) | Menarini Biotech, via Tito Speri 12, 00071 Pomezia (Roma), Italy |
| Manufacturer (finished product) | Istituto Biochimico Italiano Lorenzini, via Fossignano 2, 040011 Aprilia (Latina), Italy |
| Pharmaceutical form | Solution for injection |
| Dose | D1: 48 mg - single dose<br>D2: 100 mg - single dose<br>D3: 400 mg - single dose |
| Administration route | Intramuscular |

#### *3.1.1.2 Reference product*

| | |
|---|---|
| Placebo (P) | Placebo matching to MAD0004J08 2.5 mL solution for injection |
| Manufacturer | Istituto Biochimico Italiano Lorenzini, via Fossignano 2, 040011 Aprilia (Latina), Italy |
| Pharmaceutical form | Solution for injection |
| Dose | Not applicable (see below) |
| Administration route | Intramuscular |

### *3.1.2 Dose regimen*

Three single ascending doses (48 mg, 100 mg and 400 mg, abbreviated as D1, D2 and D3) and placebo (P) will be administered by intramuscular (i.m.) injection to three study cohorts (10 subjects/cohort) according to the following scheme:

**Table 3.1.2.1 Dose regimen scheme**

| | **Cohort 1 - 48 mg** | | **Cohort 2 - 100 mg** | | **Cohort 3 - 400 mg** | |
|---|---|---|---|---|---|---|
| | **Group 1** | **Group 2** | **Group 3** | **Group 4** | **Group 5** | **Group 6** |
| P | 1* | 1 | 1* | 1 | 1* | 1 |
| D1 | 4* | 4 | - | - | - | - |
| D2 | - | - | 4* | 4 | - | - |
| D3 | - | - | - | - | 4* | 4 |
| Tot/group | 5 | 5 | 5 | 5 | 5 | 5 |
| Tot/cohort | 10 | | 10 | | 10 | |

*Sentinel subjects will be treated one at the time at 48 h distance (after evaluation of any possible treatment-related adverse event)

Active treatment (D1/D2/D3) and placebo (P) will be assigned within each cohort and group according to the study randomisation list.

Go/No-go decision on escalation from Cohort 1 to Cohort 2 and from Cohort 2 to Cohort 3 will be taken after evaluation of Cohort 1/Cohort 2 safety data up to 48 h post-dose by the Data Safety Monitoring Board (DSMB) (see § 4.2).

### *3.1.3 Route and method of administration*

The investigational products will be administered as single doses, by i.m. injection, in the morning of day 1. For the administrations the following procedures will be followed:

Cohort 1:

- D1 (48 mg) solution for injection will be prepared by mixing 1.2 mL from one vial of Test product with 2.5 mL from one vial of Placebo and 1.3 mL from another one vial of Placebo (total 3.8 mL Placebo solution), for a total volume of 5 mL.
- P solution for injection will be prepared by mixing 1.2 mL from one vial of Placebo with 2.5 mL from another vial of Placebo and 1.3 mL from a third vial of Placebo, for a total volume of 5 mL (to maintain the blinding).

D1 or P solution (5 mL) will be administered by i.m. injection into the right gluteus (one injection/treatment only).

Cohort 2:

- D2 (100 mg) solution for injection will be prepared by mixing 1 vial of Test product and 1 vial of Placebo for a total volume of 5 mL.
- P solution for injection will be prepared by mixing 2 vials of Placebo for a total volume of 5 mL.

D2 or P solution (5 mL) will be administered by i.m. injection into the right gluteus (one injection/treatment only).

Cohort 3:

- D3 (400 mg) will be prepared as 2 solutions for injection, both obtained by mixing 2 vials of Test product [volume for each injection 5 mL (i.e. 200 mg/injection) for a total injected volume of 10 mL].
- P will also be prepared as 2 separate solutions for injection, both obtained by mixing 2 vials of Placebo (volume for each injection 5 mL, for a total injected volume of 10 mL).

For both D3 and P, two i.m. injections (5 mL each) / treatment will be performed for a total volume injected of 10 mL for each subject. For each subject, 5 mL will be injected into the right gluteus and the other 5 mL will be injected into the left gluteus.

Volumes of Test product and placebo to be administered in Cohort 1, Cohort 2 and Cohort 3 are detailed in the following scheme:

**Table 3.1.3.1 Administered volumes scheme**

| Cohort 1 | | Cohort 2 | | Cohort 3 | |
|---|---|---|---|---|---|
| **D1** | **P** | **D2** | **P** | **D3** | **P** |
| 1.2 ml Test (48 mg)<br>2.5 ml Placebo<br>1.3 ml Placebo<br>5 mL | 1.2 ml Placebo<br>2.5 ml Placebo<br>1.3 ml Placebo<br>5 mL | 2.5 ml Test (100 mg)<br>2.5 ml Placebo<br>5 mL | 2.5 ml Placebo<br>2.5 ml Placebo<br>5 mL | 2.5 ml Test (100 mg)<br>2.5 ml Test (100 mg)<br>5 mL<br>2.5 ml Test (100 mg)<br>2.5 ml Test (100 mg)<br>5 mL | 2.5 ml Placebo<br>2.5 ml Placebo<br>5 mL<br>2.5 ml Placebo<br>2.5 ml Placebo<br>5 mL |
| **Test 48 mg, 5 mL** | **Placebo, 5 mL** | **Test 100 mg, 5 mL** | **Placebo, 5 mL** | **Test 400 mg, 2×5 mL** | **Placebo, 2×5 mL** |

### *3.1.4 Investigational product distribution*

All doses will be administered by the Investigators or their deputies. The investigational products will be exclusively used for the present clinical study and will only be administered to the subjects enrolled in the study.

## 3.2 Packaging and labelling

The two products will be indistinguishable. Subjects' kits will be prepared. The primary packaging will be 2R vials. Primary packaging labels will report:

- Sponsor name
- Study number
- Investigator's name
- Site No.
- Test and Placebo Batch number
- Subject’s identification number
- Dispensed on [*date - handwritten*]
- Vial number
- Route of administration

Secondary packaging will report all the information requested according to the Annex 13 to the Good Manufacturing Practice (published by the Commission in The rules governing medicinal products in the European Community, Volume 4) as follows:

a. Name, address and telephone number of the Sponsor, contract research organisation or investigator (the main contact for information on the product, clinical study and emergency unblinding)
b. Pharmaceutical dosage form, route of administration, quantity of dosage units
c. The batch and/or code number to identify the contents and packaging operation
d. A study reference code allowing identification of the study, site, investigator and sponsor if not given elsewhere
e. The study subject kit number (and randomisation number)
f. The name of the investigator (if not included in (a) or (d))
g. Directions for use (reference may be made to a leaflet or other explanatory document intended for the study subject or person administering the product)
h. "For clinical study use only" or similar wording
i. The storage conditions
j. Period of use (use-by date, expiry date or re-test date as applicable), in month/year format and in a manner that avoids any ambiguity

Labels will be in local language.

## 3.3 Storage conditions

The investigational products will be stored at 2-8°C in a dry locked place, sheltered from light.

## 3.4 Drug accountability

The investigational products will be provided directly to the clinical centres by the Sponsor/Manufacturer), in excess of the amount necessary for the study (at least 25% excess).

After receipt of the investigational products' supply, the pharmacist will confirm in writing by signing and dating standard drug accountability forms.

At the end of the study, used, unused and partially used supplies of investigational products provided by the Sponsor/Manufacturer will either be destroyed on site (upon written authorisation) or returned to the sponsor/manufacturer, after assessment of drug accountability.

# 4 INVESTIGATIONAL PLAN

## 4.1 Overall study design

This is a first-in-human, single-dose, dose-escalation, double-blind, placebo-controlled, randomised, safety and pharmacokinetics study.

## 4.2 Discussion of design

The study has been designed in agreement with the current and applicable guideline for first-in-man studies (31), and current guidelines on immunogenicity assessment (33).

As detailed in § 1.4, the low dose proposed for MAD0004J08 in this study was calculated in order to achieve a neutralising titre in the serum of 1/100. Since most COVID-19 convalescent patients have a neutralising titre ranging from 1/20 to 1/320, it is assumed that a titre exceeding 1/100 will provide sufficient neutralising potency to eliminate the virus from the blood and the lungs.

Considering MAD0004J08 neutralisation potency of 3 ng/mL (i.e. 3 mg/kg), 21 mg for a person of 70 kg would be necessary to achieve a titre of 1/100. The proposed dose of 100 mg exceeds the titre of 1/100 by 5-fold, while the proposed titre of 400 mg exceeds the titre of 1/100 by 20-fold. In addition, according to the Italian Health Authority (Istituto Superiore della Sanita', Italy) recommendations, a starting dose of 48 mg of MAD000 4J08 will be investigated before escalation to the 100 mg and 400 mg doses, if safety concerns do not arise. The starting dose of 48 mg, as opposed to 50 mg, has been selected for technical reasons, since the volume necessary for the preparation of the solution for injection (§ 3.1.3) will be more easily withdrawn from the available 2.5 mL vials (1.2 mL as opposed to 1.25 mL).

The 48 mg, 100 mg and 400 mg doses proposed will allow administering MAD0004J08 solution by intramuscular, as opposed to intravenous injection. This route of administration could be a key advantage in emergency scenarios as it will allow giving MAD0004J08 in non-hospital care settings increasing the number of people that can quickly benefit from its foreseen therapeutic effect.

In the present study, 3 single ascending doses of 48, 100 and 400 mg MAD0004J08 will be administered by i.m. injection to healthy subjects in 3 subsequent cohorts, which include 2 groups (10 subjects) each. In details:

- Cohort 1
  - group 1 (5 sentinel subjects including 1 subject receiving placebo and 4 subjects receiving 48 mg MAD0004J08)
  - group 2 (5 subjects including 1 subject receiving placebo and 4 subjects receiving 48 mg MAD0004J08)

- Cohort 2
  - group 3 (5 sentinel subjects including 1 subject receiving placebo and 4 subjects receiving 100 mg MAD0004J08)
  - group 4 (5 subjects including 1 subject receiving placebo and 4 subjects receiving 100 mg MAD0004J08)
- Cohort 3
  - group 5 (5 sentinel subjects including 1 subject receiving placebo and 4 subjects receiving 400 mg MAD0004J08)
  - group 6 (5 subjects including 1 subject receiving placebo and 4 subjects receiving 400 mg MAD0004J08)

The sentinel subjects included in groups 1, 3 and 5 will be dosed one at the time at a 48 h distance in order to evaluate possible adverse events related to the product administration.

Safety data at 48 h post-dose for the whole cohort 1 and then for the whole cohort 2 will be analysed by the DSMB. If any major safety concerns are excluded according to the specification given in § 7.4, the study will proceed with the subsequent cohorts..

The following figure reports the enrolment and safety evaluation scheme.

**Figure 4.2.1 Enrolment and safety evaluation scheme**

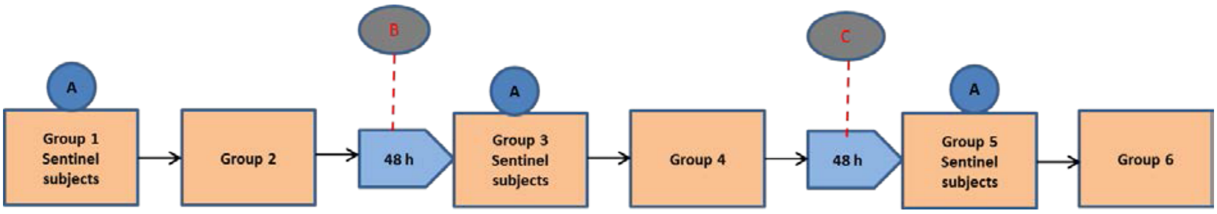


*Figure note:*
*A) Within the group (groups 1, 3 and 5), sentinel subjects will be treated one at the time with 48 h intervals to evaluate any possible treatment-related adverse events*
*B) DSMB analyses 48 h safety data for the whole cohort 1 (i.e. group 1 and group 2) and provides advice for enrolment of group 3 (cohort 2);*
*C) DSMB analyses 48 h safety data for the whole cohort 2 (group 3 and group 4) and provides advice for enrolment of group 5 (cohort 3),*

For additional details please refer to § 3.1.2.

The study design was chosen in order to minimise possible adverse reactions in each group and cohort according to the recommendations of the EMA/CHMP/SWP/28367/07 Rev. 1 "Guideline on strategies to identify and mitigate risks for first-in-human and early clinical trial with investigational medicinal products" (31).

Men and women will be enrolled in the study in order to expose a population similar to the patients' population which will be enrolled in the future studies for product development.

Each randomised subject will be allocated to a treatment arm (active or placebo) within each cohort and dosing group according to a computer generated randomisation list (see § 8.1).

The study will be double-blind with respect to active treatment vs. placebo within each cohort in order to exclude any bias in safety data evaluation.

# 5 STUDY POPULATION

## 5.1 Target population

Healthy male and female subjects

## 5.2 Inclusion criteria

To be enrolled in this study, subjects must fulfil all these criteria:

1. *Informed consent*: Signed written informed consent before inclusion in the study
2. *Full comprehension*: Ability to comprehend the full nature and purpose of the study, including possible risks and side effects; ability to co-operate with the Investigator and to comply with the requirements of the entire study
3. *Sex and age*: Healthy men and women, 18 - 55 years old, inclusive
4. *Negative SARS-CoV-2* serology test at screening (negative anti-S and anti-N)
5. *Negative SARS-CoV-2* qRT-PCR in the 72 h before treatment (test on day -3 or -2 or -1 with result before treatment)
6. *Body Mass Index*: 18.5-30 kg/m$^2$, inclusive, at screening
7. *Vital signs*: Systolic blood pressure 90-139 mmHg, diastolic blood pressure 60-90 mmHg, heart rate 50-100 bpm, measured after 5 min at rest in the supine position
8. *ECG*: Electrocardiogram without clinically significant abnormalities at screening
9. *Contraception and fertility*: Women of child-bearing potential must be using at least one of the following reliable methods of contraception and confirm to use adequate contraception during the study:
    a. Hormonal oral or implantable or transdermal, or injectable contraceptives for at least 2 months before the screening visit;
    b. A non-hormonal intrauterine device or female condom with spermicide or contraceptive sponge with spermicide or diaphragm with spermicide or cervical cap with spermicide for at least 2 months before the screening visit
    c. A male sexual partner who agrees to use a male condom with spermicide
    d. A sterile sexual partner
    e. A same sex partner

    Female participants of non-child-bearing potential or in post-menopausal status for at least 1 year will be admitted. For all women, urine pregnancy test result must be negative at screening and day 1.

## 5.3 Exclusion criteria

Subjects meeting any of these criteria will not be enrolled in the study:

1. *Physical findings*: Clinically significant abnormal physical findings which could interfere with the objectives of the study
2. *Allergy*: Ascertained or presumptive hypersensitivity to the active principle and/or ingredients of investigational products; history of anaphylaxis to drugs or allergic reactions likely to be exacerbated by any component of the investigational products in the Investigator's opinion
3. *Concomitant medications*: Medications, including over the counter (OTC) medications and herbal remedies, for 2 weeks before screening and immunoglobulin or blood products for 6 months before screening (except contraceptives or a single use of paracetamol, aspirin, or combination OTC products containing paracetamol with an antihistamine, or OTC non-steroidal anti-inflammatory drugs (NSAIDs) at a dose equal or lower than that recommended on the package; vitamins and nutritional supplements, if regularly taken before the study, are also allowed)
4. *Monoclonal Antibodies (mAb)*: Previous intake of a mAb within 6 months, or 5 antibody half-life, whichever is longer, before study start
5. *Transient acute illness:* Acute (time-limited) illness, including fever above 37.5°C on the day before or on the day of the planned treatment; subjects excluded for transient acute illness may be dosed if illness resolves within the screening period or may be rescreened once
6. *Diseases*: Significant history of renal, hepatic, gastrointestinal, cardiovascular, respiratory, skin, haematological, endocrine, psychiatric or neurological diseases that may interfere with the aim of the study or increase subjects risks; history of malignancy in the last 5 years
7. *SARS CoV-2 or COVID-19*:
   a. Participants with any confirmed current or previous COVID-19 infection at screening, or at day -1 or day 1
   b. Participant with clinical signs or symptoms consistent with COVID-19, e.g. fever, dry cough, dyspnoea, sore throat, fatigue or confirmed infection by appropriate laboratory test within the last 4 weeks before/at screening or at day -1 or day 1
   c. Any prior intake of investigational or licenced vaccine indicated for the prevention of SARS CoV-2 or COVID-19 or expected intake during follow-up period
   d. Has been reported as a case (confirmed or probable) of COVID-19 from the regional health system
8. *Immunodeficiency* due to illness, including HIV infection (positivity to anti-HIV-Ab), or due to drugs, including any course of glucocorticoid therapy exceeding 2 weeks of prednisone or equivalent within 6 months before screening

9. *Infections:* History of active infection with hepatitis B or C or positive test result for anti-HCV-Ab or HBsAg or anti-HBc-Ab at screening; history of infection with SARS or MERS
10. *Laboratory analyses*: Abnormal laboratory values that in the opinion of the Investigator are clinically significant
11. *Investigative drug studies*: Participation in the evaluation of any investigational product for 6 months before this study
12. *Blood donation*: blood donations for 3 months before the study, during the study and in the 3 months after the end of the study
13. *Drug test*: positive drug test at screening or day -1
14. *Drug, alcohol*: history of drug or alcohol abuse within 6 months before screening
15. *Pregnancy (women only)*: positive or missing pregnancy test at screening or day 1; pregnant or lactating women
16. *Other:* Any condition that might compromise study subject's safety or interfere with the study evaluations or interpretation of subject's safety or study results

***IMPORTANT NOTE 1:*** *Subjects screened and kept as reserve or not enrolled for any transient reason can be rescreened once for the study, according to the Investigator's opinion*

***IMPORTANT NOTE 2:*** *In case, during the study, a subject is included in a vaccination list according to the present national guidelines, the possibility of blinding opening, the assigned treatment and the possible vaccination will be discussed. In any case the best option for the subject will be pursued*

### *5.3.1 Re-screening*

Volunteers who meet selection criteria but were not randomized within 21 days or did not meet 1 or more selection criteria can be re-screened within 3 months from the previous screening with the approval of the Sponsor. In this case, volunteers should be considered as screening failure and the reason documented in the source document and electronic Case Report Form (eCRF). For the re-screening volunteer needs to sign a new Informed Consent Form, new Subject screening number is assigned and all screening procedures completed again.

### *5.3.2 Not allowed treatments*

No medications, including over the counter medications (OTC) and herbal remedies, are allowed for 2 weeks before screening and for the whole study duration. Immunoglobulin or blood products are not allowed for 6 months before screening and during all the study. Exceptions are contraceptives, a single use of paracetamol, aspirin, or combination OTC products containing paracetamol with an antihistamine, OTC NSAID at a dose equal or lower than that recommended on the package, which are allowed. Vitamins and other nutritional supplements, if regularly taken before the study, are also allowed.
The intake of any other medication will be reported as a protocol deviation. However, it will lead to subject's discontinuation from the study only if the investigator, together with the sponsor, considers it could affect the study assessments or outcome.

# 6 STUDY SCHEDULE

The schedule of the study is summarised at page 15.

## 6.1 Study visits and procedures

Each subject completing the study will undergo 12 visits. Study duration for each subject will be approximately 6 months.
A written informed consent will be obtained before any study assessment or procedure, including screening procedures.
The first subject first visit (FSFV) is defined as the 1st visit performed by the 1st screened subject at one clinical centre. The last subject last visit (LSLV) is defined as the last visit performed by the last subject, i.e. the last visit foreseen by the study protocol, independently of the fact that the subject is a completer or a withdrawn subject.

The following phases, visits and procedures will be performed for all three cohorts:

- **Screening phase**
  - Screening – Visit 1: between day -21 and day -2
  - Visit 2: day -1 - clinical centre confinement in the evening

- **Interventional phase**
  - Visit 3: day 1

- **Observational phase**
  - Visit 4: day 2
  - Visit 5: day 3

- **Follow-up phase**
  - Visit 6: day 8 (week 1)
  - Visit 7: day 15±2 (week 2)
  - Visit 8: day 22±2 (week 3)
  - Visit 9: 1 month (day 30±3)
  - Visit 10: 2 months (day 60±4)
  - Visit 11: 4 months (day 120±4)

- **Final phase**
  - Visit 12 – 6 months (day 180±7) – Final visit
  - Early termination visit (ETV). In case of early discontinuation, as far as possible discontinued subjects will undergo an early termination visit (ETV)

Clinical activities and procedures are detailed in the table below:

**Table 6.1.1 Clinical Procedures and assessments - summary**

| **Clinical procedures and assessments, continued:** | | |
|---|---|---|
| **Visit / Day** | **Procedures/Assessments** | **Notes** |
| **Visit 1 - Screening**<br>**Day -21 / Day -2** | ➢ Explanation to the subject of study aims, procedures and possible risks<br>➢ Informed consent signature<br>➢ Screening number assignment (as S001, S002, etc.)<br>➢ Demographic data and life style recording<br>➢ Medical history<br>➢ Previous/concomitant medications<br>➢ Physical examination (including body weight, height)<br>➢ Vital signs (blood pressure, heart rate, body temperature)<br>➢ 12-lead ECG<br>➢ SARS-CoV-2 serology test<br>➢ Laboratory analyses: haematology, blood chemistry, urinalysis, serum virology, coagulation, ferritin<br>➢ Drug of abuse test<br>➢ Urinary pregnancy test (women)<br>➢ SARS-CoV-2 qRT-PCR in the 72 h before day 1 (could be performed on day -3 or day-2 or day -1 / see below)<br>➢ Adverse event monitoring<br>➢ Inclusion/exclusion criteria evaluation<br>➢ Eligibility evaluation | Ambulatory visit<br><br>*Note:*<br>*From day 1 (visit 3) to day 8 (Visit 6; diary 1) /month 1 (Visit 9; diary 2)), the subjects will daily record in 2 diaries any change in physical or medical conditions and the intake of any concomitant treatment.*<br><br>*The investigator will check the diary at each visit (up to Visit 6 and Visit 9, respectively, for the 2 diaries).* |
| **Visit 2 - Day -1** | ➢ SARS-CoV-2 qRT-PCR in the 72 h before treatment. On day -1 either test performance, or result check if already performed on day -3 or day -2<br>➢ Drug of abuse test<br>➢ Inclusion/exclusion criteria evaluation<br>➢ Check of AEs and concomitant medications<br>➢ Vital signs (blood pressure, heart rate, body temperature)<br>➢ Physical examination<br>➢ Eligibility evaluation | Arrival at the clinical centre in the evening of day -1.<br>Confinement until the morning of day 2 (see below)<br>Standardised dinner |
| **Visit 3 - Day 1** | ➢ Urine pregnancy test (women)<br>➢ Inclusion/exclusion criteria evaluation, eligibility evaluation, and randomisation<br>➢ 12-lead ECG<br>➢ Dispensation of 2 diaries to the subject (diary 1 to report solicited adverse events from day 1 to day 8, diary 2 to report all unsolicited adverse events and concomitant medication from day 1 to month 1)<br>➢ Laboratory analyses: haematology, coagulation, blood chemistry, urinalysis, SARS-CoV-2 serology test - baseline<br>➢ Blood sample collection for pharmacokinetic analysis at pre-dose (0) and 1h, 2h, 3h, 4h, 6h, 8h, 12h post-dose<br>➢ Blood sample collection for ADA analysis at pre-dose (0)<br>➢ Blood sample collection for serum neutralising power test at pre-dose (0)<br>➢ Blood sample collection for DIESSE Elisa kit characterisation at pre-dose (0)<br>➢ Vital signs (blood pressure, heart rate, body temperature) measurement at pre-dose (0), 2h post-dose and 12 h post- | Confinement<br>Standardised meals at the clinical centre |

| | | |
|---|---|---|
| | dose<br>➢ Investigational product administration (active or placebo according to study Cohort, Group and randomisation)<br>➢ Adverse events monitoring<br>➢ Injection site reactions check<br>➢ Concomitant medications check | |
| **Visit 4 - Day 2** | ➢ Physical examination<br>➢ Vital signs (blood pressure, heart rate, body temperature) measurement at 24 h post-dose<br>➢ Blood sample collection for pharmacokinetic analysis at 24h post-dose<br>➢ Adverse events monitoring<br>➢ Injection site reactions check<br>➢ Diary 1 and diary 2 check<br>➢ Concomitant medications check<br>➢ Laboratory analyses: haematology, blood chemistry, coagulation, urinalysis, SARS-CoV-2 serology test at 24 h post-dose<br>➢ 12-lead ECG at 24 h post-dose | Confinement<br><br>Discharge in the morning of Day 2, after the 24 h post-dose blood sampling<br>Upon leaving, the subjects will be instructed to contact immediately the Investigator in case of occurrence of any adverse events |
| **Visit 5 - Day 3** | ➢ Vital signs (blood pressure, heart rate, body temperature) measurement at 48 h post-dose<br>➢ Blood sample collection for pharmacokinetic analysis at 48h post-dose<br>➢ Blood sample collection for serum neutralising power test at 48 h post-dose<br>➢ Blood sample collection for DIESSE Elisa kit characterisation at 48 h post-dose<br>➢ Adverse events monitoring<br>➢ Injection site reactions check<br>➢ Diary 1 and diary 2 check<br>➢ Concomitant medications check<br>➢ Laboratory analyses: haematology, blood chemistry, coagulation, urinalysis, SARS-CoV-2 serology test - at 48 h<br>➢ Physical examination | Ambulatory visit |
| **Visits 6 / Week 1 / Day 8**<br><br>**and**<br><br>**Visit 7 / Week 2 / Day 15±2** | ➢ Physical examination<br>➢ Vital signs (blood pressure, heart rate, body temperature) measurement<br>➢ Blood sample collection for pharmacokinetic analysis<br>➢ Blood sample collection for ADA analysis<br>➢ Blood sample collection for serum neutralising power test on day 8 only<br>➢ Blood sample collection for DIESSE Elisa kit characterisation on day 8 only<br>➢ Adverse events monitoring<br>➢ Concomitant medications check<br>➢ Laboratory analyses: haematology, blood chemistry, coagulation, urinalysis, SARS-CoV-2 serology test<br>➢ Diary 1 check - day 8 only<br>➢ Diary 2 check - both days<br>➢ Subjects' diary 1 return - day 8 only | Ambulatory visit |
| **Visit 8**<br>**Week 3**<br>**Day 22±2** | ➢ Physical examination<br>➢ Vital signs (blood pressure, heart rate, body temperature)<br>➢ Blood sample collection for pharmacokinetic analysis<br>➢ Adverse events monitoring<br>➢ Diary 2 check | Ambulatory visit |

| | | |
|---|---|---|
| | ➢ Concomitant medications check<br>➢ Laboratory analyses: haematology, blood chemistry, coagulation, urinalysis, SARS-CoV-2 serology test | |
| **Visit 9 - 1 month**<br>**Day 30±3** | ➢ Physical examination<br>➢ Vital signs (blood pressure, heart rate, body temperature) measurement<br>➢ Blood sample collection for pharmacokinetic analysis<br>➢ Blood sample collection for ADA analysis<br>➢ Blood sample collection for serum neutralising power test<br>➢ Blood sample collection for DIESSE Elisa kit characterisation<br>➢ Adverse events monitoring<br>➢ Diary 2 check<br>➢ Concomitant medications check<br>➢ Laboratory analyses: haematology, blood chemistry, coagulation, urinalysis, SARS-CoV-2 serology test<br>➢ Urine pregnancy test (women)<br>➢ Subjects' diary 2 return | Ambulatory visit |
| **Visit 10 - 2 months**<br>**Day 60±4** | ➢ Physical examination<br>➢ Vital signs (blood pressure, heart rate, body temperature) measurement<br>➢ Blood sample collection for pharmacokinetic analysis<br>➢ Adverse events monitoring<br>➢ Concomitant medications check<br>➢ Laboratory analyses: haematology, blood chemistry, coagulation, urinalysis, SARS-CoV-2 serology test<br>➢ Urine pregnancy test (women) | Ambulatory visit |
| **Visit 11 - 4 months**<br>**Day 120±4 days** | ➢ Physical examination<br>➢ Vital signs (blood pressure, heart rate, body temperature) measurement<br>➢ Blood sample collection for pharmacokinetic analysis<br>➢ Blood sample collection for ADA<br>➢ Blood sample collection for serum neutralising power test<br>➢ Blood sample collection for DIESSE Elisa kit characterisation<br>➢ Adverse events monitoring<br>➢ Concomitant medications check<br>➢ Laboratory analyses: haematology, blood chemistry, coagulation, urinalysis, SARS-CoV-2 serology test<br>➢ Urine pregnancy test (women) | Ambulatory visit |
| **Visit 12 - 6 months**<br>**Final visit**<br>**Day 180±7 days** | ➢ Physical examination (including body weight)<br>➢ Vital signs (blood pressure, heart rate, body temperature) measurement<br>➢ Blood sample collection for pharmacokinetic analysis<br>➢ Blood sample collection for ADA<br>➢ Blood sample collection for serum neutralising power test<br>➢ Blood sample collection for DIESSE Elisa kit characterisation<br>➢ Saliva sample collection for IgG analysis and neutralising power test<br>➢ Adverse events monitoring<br>➢ Concomitant medications check<br>➢ Laboratory analyses: haematology, blood chemistry, urinalysis, coagulation, SARS-CoV-2 serology test | Ambulatory visit<br><br>In case of premature discontinuation, the same assessments foreseen as for visit 12 will be performed as far as possible at the time of discontinuation (Early termination visit) |

| | | |
|---|---|---|
| | ➢ 12-lead ECG | |

## 6.2 Restrictions, diet and lifestyle

The subjects will reside at the clinical centre from the evening of day -1 until the morning of day 2. On day 1, the subjects will remain fasted until standardised lunch is served.

When resident at the clinical centre, subjects will receive standardised meals (days -1 and day 1), as appropriate. During confinement, routine ambulant daily activities will be recommended, whereas hazardous, strenuous or athletic activities will not be permitted.

On the other study days (Visits 5-12), the subjects will attend the clinical centre for ambulatory visits in fasted condition and strenuous or athletic activities in the previous 48 hours will be discouraged.

# 7 DESCRIPTION OF SPECIFIC PROCEDURES

## 7.1 Physical examination

Physical examinations will be performed at screening (Visit 1), at Visits 2, 4-11 and at final visit/ETV.

Significant findings/illnesses, reported after the start of the study and that meet the definition of an AE (see § 11), will be recorded in the subject source documents.

Date of the physical examination, overall Investigator's interpretation (as normal or abnormal and, if abnormal, clinically significant or not clinically significant) and clinically significant abnormalities (if any) will be reported in the eCRF.

### *7.1.1 Body weight, height and BMI*

Body weight will be recorded at screening and final visit/ETV.
Subjects will be weighed (kg) lightly clothed without shoes. Height will be measured at screening only and BMI will be recorded. BMI will be calculated as weight [kg]/(height [m] x height [m]).

### *7.1.2 Vital signs*

Subjects' blood pressure, heart rate and body temperature will be measured by the Investigators or their deputies after 5 min at rest at:

- Visit 1 - Screening
- Visit 2 - day -1
- Visit 3 - day 1, at pre-dose (baseline), 2 h and 12 h post-dose
- Visits 4-5: days 2-3: at 24 and 48 h post-dose
- Visits 6-11: day 8, day 15±2, day 22±2, Month 1 (day 30±3), Month 2 (day 60±4), Month 4 (day 120±4)
- Visit 12: Month 6 (day 180±7) or ETV (final assessment)

Date/time of the vital signs assessment and clinically significant abnormalities (if any) will be reported in the individual eCRFs. All clinically significant abnormalities after the screening visit will be recorded as AEs.

### *7.1.3 ECGs*

12-Leads ECGs will be performed at:

- Screening
- Visit 3: day 1 pre-dose
- Visit 4: day 2: 24 h
- Month 6 (day 180±7) or ETV (final assessment)

Date/time of the ECG recording, overall Investigator's interpretation (as normal or abnormal and, if abnormal, clinically significant or not clinically significant) and clinically significant abnormalities (if any) will be reported in the individual eCRFs. All clinically significant abnormalities after the screening visit will be recorded as AEs.

## 7.2 Clinical laboratory assays

Samples of blood and urine will be collected at screening.

The following laboratory analyses will be performed <u>at the screening visit</u>:

**HAEMATOLOGY**
Leukocytes and leukocyte differential count (percentage values and absolute values), erythrocytes, haemoglobin (g/dL), haematocrit, MCV, MCH, MCHC, platelets, PDW.

**COAGULATION**
aPTT, INR

**BLOOD CHEMISTRY**
**Electrolytes:** sodium, potassium, calcium, chloride
**Enzymes:** alkaline phosphatase, γ-GT, AST, ALT
**Substrates/metabolites:** total bilirubin, creatinine, fasting glucose, urea, uric acid, total cholesterol, triglycerides, ferritin (only screening, see below)
**Proteins:** total proteins, albumin
C-reactive protein

**Urine pregnancy test (women)**

**URINE ANALYSIS**
**Urine chemical analysis** (stick)**:** pH, specific weight, appearance, colour, nitrites, proteins, glucose, urobilinogen, bilirubin, ketones, haematic pigments, leukocytes
**Urine sediment** (analysis performed only if positive): leukocytes, erythrocytes, flat cells, round cells, crystals, cylinders, mucus, bacteria

**SERUM VIROLOGY**
**Hepatitis B** (HBsAg;anti-HBc-Ab total), **Hepatitis C** (HCV antibodies), **HIV 1/2** (HIV Ag/Ab combo). SARS CoV-2 IgG anti-N and anti-S and SARS-CoV-2 RT-PCR.
SARS - CoV-2 qRT-PCR must be available 72 h before treatment (done or checked on day -1).

The same analyses, with the exception of ferritin, virology and pregnancy test, will be repeated on day 1 at baseline, and then at each visit up to final visit/ETV.

A SARS-CoV-2 IgG serology test (anti-N only) will be performed at all visits from Visit 3 to Visit 12 or ETV.
A SARS-CoV-2 test will be repeated during the study if a subjects presents with symptoms or according to the Investigator's opinion.

For fertile women, a urine pregnancy test will be performed at screening, at day 1 and at 1 month, 2 months, 4 months post-dose.

Drugs of abuse use will be assessed at screening and on day -1.

Date/time of samples collection, overall Investigator's interpretation (as normal or abnormal and, if abnormal, clinically significant or not clinically significant) and clinically significant findings (if any) will be reported in the eCRF. All clinically significant abnormalities after the screening visit will be recorded as AEs.

The total volume of blood that will be drawn for safety laboratory tests and serology is between 148.5 and 160.5 mL. Details are reported in the table below:

**Table 7.2.1 Total volume of blood for safety laboratory tests and serology**

| Visit | Haematology | Biochemistry | Coagulation | HBV, HCV | HIV-Ab | Sars CoV2 | Total |
|---|---|---|---|---|---|---|---|
| Screening | 3 mL | 3.5 mL | 1 mL | 5 or 6 mL* | 6 mL | 5 or 6 mL* | **23.5 - 25.5 mL*** |
| Pre-dose (baseline) | 3 mL | 3.5 mL | 1 mL | NA | NA | 5 or 6 mL* | **12.5-13.5 mL** |
| 24 h | 3 mL | 3.5 mL | 1 mL | NA | NA | 5 or 6 mL* | **12.5-13.5 mL** |
| 48 h | 3 mL | 3.5 mL | 1 mL | NA | NA | 5 or 6 mL* | **12.5-13.5 mL** |
| Day 8 | 3 mL | 3.5 mL | 1 mL | NA | NA | 5 or 6 mL* | **12.5-13.5 mL** |
| Day 15 | 3 mL | 3.5 mL | 1 mL | NA | NA | 5 or 6 mL* | **12.5-13.5 mL** |
| Day 22 | 3 mL | 3.5 mL | 1 mL | NA | NA | 5 or 6 mL* | **12.5-13.5 mL** |
| 1 month | 3 mL | 3.5 mL | 1 mL | NA | NA | 5 or 6 mL* | **12.5-13.5 mL** |
| 2 months | 3 mL | 3.5 mL | 1 mL | NA | NA | 5 or 6 mL* | **12.5-13.5 mL** |
| 4 months | 3 mL | 3.5 mL | 1 mL | NA | NA | 5 or 6 mL* | **12.5-13.5 mL** |
| 6 months | 3 mL | 3.5 mL | 1 mL | NA | NA | 5 or 6 mL* | **12.5-13.5 mL** |
| **Total** | **33 mL** | **38.5 mL** | **11 mL** | **5-6 mL*** | **6 mL** | **55-66mL*** | **148.5-160.5 mL*** |

**** Total volume of blood will be 148.5 mL at clinical centre N. 1 and 160.5 mL at clinical centre N. 2***

## 7.3 Treatment-emergent adverse events

AEs will be assessed throughout the study from the signature of the informed consent up to the end of the study (6 months; day 180±7).

For the definition and management of adverse events (AEs), treatment-emergent adverse events (TEAEs) and serious adverse events please refer to § 11.

Severe adverse events and serious adverse events will be the primary study endpoint to evaluate safety of the study treatments. Clinically significant abnormal laboratory values and vital signs as well as adverse reactions at the injection site will be reported as AEs and considered in the evaluation.

### *7.3.1 Subject's diaries for reporting solicited and unsolicited adverse events*

This section focuses on participant diaries. For definitions of AEs, solicited and unsolicited AEs and serious AEs (SAEs), please refer to § 11.

At Visit 3, each participant will be given two diaries and asked to record information through day 7 (diary 1) and Visit 9 (diary 2) as follows:

Diary 1 (for solicited AEs): From Day 1 (Visit 3) to Day 7: record and score once per day a predefined set of 3 local and 8 systemic solicited AEs as described in § 7.3.2 below.

Diary 2 (for unsolicited AEs): From Day 1 to the end of Month 1 (Visit 9): record as free text any other AE (unsolicited AEs) and use of any concomitant medication.

The Investigator (or designee) will review the diaries at each visit through Visit 6 (diary 1) and Visit 9 (diary 2). Participants will return to the Investigator (or designee) diary 1 at Visit 6 and diary 2 at Visit 9.

### *7.3.2 Recording and severity grading of solicited AEs*

The grading scales used to assess local and systemic solicited AEs are derived from the FDA Center for Biologics Evaluation and Research (CBER) guidelines on toxicity grading scales for healthy adult volunteers enrolled in preventive vaccine clinical trials (36).

Solicited AEs will be recorded and graded by the participant once per day (best in the evening before bed time, approximately at the same time each day) according to the grading scales reported in Table 7.3.1 and Table 7.3.2 for local AE and systemic AE, respectively.

If the same solicited AEs occurs more than once on the same day, the worst grading score for that day will be recorded.

Subjects in Cohort 3 who receive 2 injections should record the worst score for each of the 3 local symptoms/signs.

**Table 7.3.1 Solicited local AE grading scale**

| AE (site of injection) | Grade | | Description |
|---|---|---|---|
| Pain at injection site | 0 | none | No pain |
| | 1 | mild | Does not interfere with activity |
| | 2 | moderate | Interferes with activity |
| | 3 | severe | Prevents activity |
| Redness | 0 | none | No redness |
| | 1 | mild | > 2.0 cm to 5.0 cm |
| | 2 | moderate | > 5 cm to 10.0 cm |
| | 3 | severe | >10 cm |
| Swelling | 0 | none | No swelling |
| | 1 | mild | 2.5 – 5 cm and does not interfere with activity |
| | 2 | moderate | 5.1 - 10 cm or interferes with activity |
| | 3 | severe | >10 cm or prevents daily activity |

**Table 7.3.2 Solicited systemic AE grading scale**

| AE | Grade | | Description |
|---|---|---|---|
| Vomiting | 0 | none | No vomiting |
| | 1 | mild | 1-2 times in 24 hours |
| | 2 | moderate | > 2 times in 24 hours |
| | 3 | severe | Requires IV hydration |
| Diarrhoea | 0 | none | No diarrhoea |
| | 1 | mild | 2-3 loose stools in 24 h |
| | 2 | moderate | 4-5 loose stools in 24 h |
| | 3 | severe | 6 or more loose stools in 24 h |
| Headache | 0 | none | No headache |
| | 1 | mild | Does not interfere with activity |
| | 2 | moderate | Some interference with activity |
| | 3 | severe | Prevents daily routine activity |
| Fatigue/tiredness | 0 | none | No fatigue/tiredness |
| | 1 | mild | Does not interfere with activity |
| | 2 | moderate | Some interference with activity |
| | 3 | severe | Prevents daily routine activity |
| Chills | 0 | none | No chills |
| | 1 | mild | Does not interfere with activity |
| | 2 | moderate | Some interference with activity |
| | 3 | severe | Prevents daily routine activity |
| New or worsened muscle pain | 0 | none | No new or worsened muscle pain |
| | 1 | mild | Does not interfere with activity |
| | 2 | moderate | Some interference with activity |
| | 3 | severe | Prevents daily routine activity |

| | | | |
|---|---|---|---|
| New or worsened joint pain | 0 | none | No new or worsened joint pain |
| | 1 | mild | Does not interfere with activity |
| | 2 | moderate | Some interference with activity |
| | 3 | severe | Prevents daily routine activity |
| Fever | 0 | none | No fever |
| | 1 | mild | ≥38.0-38.4°C |
| | 2 | moderate | ≥38.4-38.9°C |
| | 3 | severe | ≥38.9-40.0°C* |

If a participant records one or more solicited AEs as severe (grade 3), he/she will contact the study staff and may be asked to attend the centre.

If a solicited AE is classified as serious (see definition, § 11.2), management of the event will follow SAE management and reporting procedures detailed in § 13. If an event requires emergency room visit or hospitalisation for any reason, it will be reported as a SAE. Necrosis and exfoliative dermatitis cases will always be reported as SAE.

The end date of a solicited local event will be the first of 2 consecutive days scored as 0. If the AE is still ongoing on Day 8 (Visit 6), the event will be followed up until its resolution.

Diary 1 will be checked and solicited AEs reported in the diary will be entered in the eCRF.

#### *7.3.3 Unsolicited AEs*

Any unsolicited AEs reported by the subjects in diary 2 and any other AE experienced by the subjects or observed by the Investigator will be recorded, graded, classified and reported as detailed in § 11.

### 7.4 Safety data evaluation at 48 h post-dose

Go/No-go decision on escalation from Cohort 1 to Cohort 2 and from Cohort 2 to Cohort 3 will be taken after evaluation of Cohort 1/Cohort 2 safety data at 48 h post-dose by the DSMB.

Data evaluated will include TEAEs (solicited and unsolicited), with particular attention to severe and serious AEs judged related to the investigational product, clinically significant laboratory analysis results, vital signs, adverse events at the injection site and physical examination outcome.

After evaluation, the DSMB will make recommendations and the Sponsor will take the final decision based on DSMB recommendations.

## 7.5 Sampling for pharmacokinetic, ADA, serum neutralising power, Diesse kit and saliva samples analyses

### *7.5.1 Venous blood sampling*

Venous blood samples (10 mL) will be collected from a forearm vein at the following times:

- pre-dose (0h) and at 1h, 2h, 3h, 4h, 6h, 8h, 12h, 24h, 48h post-dose, on day 8, day 15 (±2), day 22 (±2), at 1 month (day 30±3), 2 months (day 60±4), 4 months (day 120±4) and 6 months (day 180±7).

Sample collected at all time-points will be used for PK analysis (17 samples / subject), whereas ADA analysis will be performed on the samples collected at the following time-points only:

- pre-dose (0h) and on day 8, day 15 (±2), at 1 month (day 30±3), 4 months (day 120±4) and 6 months (day 180±7) (6 samples/subject)

Additional blood samples (7 mL) will be collected separately for serum neutralising power test at:

- pre-dose (0h) and 48 h post-dose, on day 8, at 1 month (day 30±3), 4 months (day 120±4) and 6 months (180±7) (6 samples/subject)

In addition, blood samples (2 mL) will be collected separately for DIESSE Elisa kit characterisation at:

- pre-dose (0h) and 48 h post-dose, on day 8, at 1 month (day 30±3), 4 months (day 120±4) and 6 months (180±7) (6 samples/subject)

Actual sampling times for each subject will be recorded in the individual eCRFs. The actual sampling times should not exceed the recommended tolerance ranges presented in the following table. Any deviation outside the recommended ranges will be verified through a query in the eCRF and, if confirmed, will be reported as protocol deviation, although it will not automatically lead to the exclusion of the concerned subjects from the analysis.

**Table 7.5.1.1 Tolerance ranges for the scheduled sampling times**

| Sampling time | Tolerance range |
|---|---|
| Pre-dose (0) | Within 30 minutes before IMP administration |
| 1h | ± 3 min |
| 2, 3, 4 h | ± 5 min |
| 6, 8, 12, 24 h | ± 10 min |
| 48 h | ± 30 min |
| Day 8 | ± 1 day |
| Day 15 | ± 2 days |
| Day 22 | ± 2 days |
| Month 1 | ± 3 days |
| Months 2, 4 | ± 4 days |
| Month 6 | ± 7 days |

#### *7.5.1.1 Sample preparation for PK and ADA analysis*

After collection, the samples will be kept at room temperature for a time between 30 and 60 min, and then centrifuged at 2500×g for 10 min at room temperature.

After collection at the time points for PK analysis only, serum samples will be divided in 2 aliquots, S1 and S2, into pre-labelled polyethylene tubes and stored frozen at ≤-70° C until analyses.

At the collection times where serum samples are prepared for both PK and ADA analysis, i.e. at pre-dose (0h), day 8, day 15, month 1, month 4 and month 6, serum samples will be divided in 4 aliquots, i.e. S1 and S2 for PK analysis plus A1 and A2 for ADA analysis, into pre-labelled polyethylene tubes. All aliquots S1/S2/A1/A2 will be stored frozen at ≤-70° C until analyses.

#### *7.5.1.2 Sample preparation for serum neutralising power test*

After collection into collection tubes, the samples will be kept at room temperature for a time between 30 and 60 min, and then centrifuged at 2500×g for 10 min at room temperature.

Serum samples will be divided in 2 aliquots, N1 and N2, into pre-labelled vacutainers and stored frozen at ≤-20°C until analyses.

#### *7.5.1.3 Sample preparation for DIESSE kits characterisation*

After collection into collection tubes, the samples will be kept at room temperature for a time between 30 and 60 min, and then centrifuged at 2500×g for 10 min at room temperature.

Serum samples will be divided in 2 aliquots, K1 and K2, into pre-labelled vacutainers and stored frozen at ≤-20°C until analyses.

### *7.5.2 Saliva sampling*

Saliva samples (one sample/patient) will be collected at 6 months (day 180±7) post-dose, by passive drool using the Saliva Collection Aid (SCA) (Saliva Bio, Salimetrics), directly into a pre-labelled collection vial (SS sample).

Immediately after collection the vial will be closed and the sample will be frozen and kept at -20°C. If freezing is not possible, the sample will be refrigerated immediately at 4°C and maintained at this temperature for no longer than necessary (ideally less than 2 hours) before freezing at or below -20°C.

### *7.5.3 Analytics - PK and ADA*

Serum concentration of MAD0004J08 will be determined at Ardena Bioanalysis B.V., The Netherlands, using an ELISA method, validated according to the current FDA and EMA guidelines for bioanalytical methods validation.

The method for detection of MAD0004J08 in human serum is a quantitative sandwich ELISA. A 96-wells plate is coated with SARS-CoV-2 Spike protein. After blocking of the plate, samples containing MAD0004J08 are pipetted into the wells, followed by a wash to remove all unbound matrix components. Alkaline phosphatase labelled anti-Human IgG (γ chain specific) is added to bind to the immobilised MAD0004J08. The complex is detected by pNPP substrate. After a wash to remove unbound reagents, the enzyme is revealed by its action on the pNPP substrate. After stopping the reaction with a strong acid, the intensity of the colour (read at 405 nm) is directly proportional to the amount of MAD0004J08 present in the sample. The method specificity will be validated before bioanalysis. Further details will be found in the method validation plan and in the bioanalytical plan.

ADA presence will be determined in serum samples at at Ardena Bioanalysis B.V., The Netherlands.

Analyses will be performed according to the general Principles of “OECD Good Laboratory Practices for testing of chemicals" C(81) 30 (final) and GCP.

The method validation report and the analytical reports will be attached to the final report.

### 7.5.4 Analytics - neutralisation test

Serum neutralisation power in serum samples will be assessed either at VisMederi Srl, Italy or at the Laboratorio di Virologia, Istituto Nazionale per le Malattie Infettive Lazzaro Spallanzani, IRCCS, Italy. A final decision about laboratory for this analysis will be taken before first subject first visit.

### 7.5.5 Analytics - DIESSE Elisa kit characterisation

Diesse kits test on collected serum samples will be performed at Toscana Life Sciences, Italy.

### 7.5.6 Analytics - Saliva samples analysis

IgG analysis and neutralisation power test in saliva samples will be performed at VisMederi Srl, Italy.

### 7.5.7 Labelling, storage and transport of samples

#### 7.5.7.1 Samples labelling

Each sample tube will be clearly and unequivocally identified with a label resistant to the storage temperature and reporting:

| | |
|---|---|
| Study code | Study CRO-20-144 - Sponsor code A0001A |
| Subject randomisation number | |
| Tube identification | S1/S2 or A1/A2 or N1/N2 or K1/K2 or SS |
| Cohort N. | 1/2/3 |
| Study day | |

Scheduled sampling time as h or day; see § 7.5.1

#### *7.5.7.2 Samples storage and transport*

Samples storage for PK/ADA analysis

During the study S1/S2/A1/A2 samples will be stored at ≤- 70° C (brief fluctuations are possible when freezers are opened) and SS samples will be stored at ≤- 20° C.

All S1 and A1 aliquots, packed in sufficient solid $CO_2$, will be shipped by an authorised courier from the clinical centre to the analytical laboratory. Temperature will be tracked with in place data-loggers.

S1 and A1 aliquots will remain stored at Ardena Bioanalysis B.V. for a maximum time of 3 months after completion of the bioanalytical report. After this period, the Sponsor will be informed that storage will be terminated. The Sponsor then will decide whether sample storage can be prolonged or the samples can be destroyed, forwarded or returned.

The counter-samples (S2 and A2 aliquots) will remain stored at each site up to three months after the end of the clinical phase. These samples will either be:

- sent to the laboratory for reanalysis should this become necessary for analytical reasons or if any problems occur during the delivery of aliquots 1, or
- destroyed at an authorised site, or
- transferred to the sponsor upon written request.

Samples storage for serum neutralising activity test

During the study N1/N2 serum samples will be stored at ≤-20° C (brief fluctuations are possible when freezers are opened).

All N1 samples will be shipped by an authorised courier from the clinical centre to either VisMederi Srl, Italy, or at the Laboratorio di Virologia, Istituto Nazionale per le Malattie Infettive Lazzaro Spallanzani, IRCCS, Italy. Temperature will be tracked with in place data-loggers. N1 samples will remain stored at VisMederi Srl, Italy, or at Spallanzani virology laboratory, Italy, for a maximum time of 3 months after completion of the assay report. After this period, the Sponsor will be informed that storage will be terminated. The Sponsor then will decide whether sample storage can be prolonged or the samples can be destroyed, forwarded or returned.

The counter-samples (N2 aliquots) will remain stored at each site up to three months after the end of the clinical phase. These samples will either be sent to the laboratory for reanalysis should this become necessary for analytical reasons or if any problems occur during the delivery of aliquots 1, or destroyed at an authorised site, or transferred to the sponsor upon written request.

Samples storage for DIESSE Elisa kit characterisation

During the study K1/K2 serum samples will be stored at ≤-20° C (brief fluctuations are possible when freezers are opened).

All K1 samples will be shipped by an authorised courier from the clinical centre to Toscana Life Science, Italy. Temperature will be tracked with in place data-loggers. Samples will remain stored at Toscana Life Science, Italy, for a maximum time of 3 months after completion of the assay report. After this period, the Sponsor will be informed that storage will be terminated. The Sponsor then will decide whether sample storage can be prolonged or the samples can be destroyed, forwarded or returned.

The counter-samples (K2 aliquots) will remain stored at each site up to three months after the end of the clinical phase. These samples will either be sent to the laboratory for reanalysis should this become necessary for analytical reasons or if any problems occur during the delivery of aliquots 1, or destroyed at an authorised site, or transferred to the sponsor upon written request.

<u>Saliva samples storage and transport</u>
During the study SS samples will be stored at ≤-20° C (brief fluctuations are possible when freezers are opened).
All SS samples will be shipped by an authorised courier from the clinical centre to VisMederi Srl, Italy. Temperature will be tracked with in place data-loggers. SS samples, if applicable, will remain stored at VisMederi Srl, Italy, for a maximum time of 3 months after completion of the assay report. After this period, the Sponsor will be informed that storage will be terminated. The Sponsor then will decide whether sample storage can be prolonged or the samples can be destroyed, forwarded or returned.

No analyses different from those stated in this protocol and agreed by the subjects when signing the informed consent form will be performed unless a new informed consent and a new approval from the Ethical Committee is obtained. The subjects may ask to destroy their own samples at any time.

## 7.6 Total number of blood samples and blood withdrawn

During the study the following volume of blood will be collected for PK/ADA analysis and for serum neutralising power test:

- PK/ADA: 10 mL x max 17 time-points=170 mL
- Neutralising test: 7 mL x 6 time-points=42 mL
- Diesse kits characterisation: 2 mL x 6 time-points=12 mL

In total 224 mL of blood will be withdrawn from each subject throughout the study in addition to the blood withdrawn for the safety clinical laboratory assays (§ 7.2).

Total blood volume including blood for safety laboratory assays collected from each subject during the whole study will be 224 mL + 148.5-160.5 mL= 372.5 - 384.5 mL.

# 8 ASSIGNMENT OF STUDY TREATMENT

## 8.1 Randomisation

The randomisation list will be computer-generated by the provider of the eCRF according to the study design.

The kit list will be generated by the Biometry unit of CROSS Research.

The randomisation number includes the 1-digit site code (e.g. 1, 2), the 1-digit cohort code (e.g. 1, 2, 3), a unique progressive 2-digit number within each cohort and site (i.e. 01, 02, 03 …). For example, according to this structure, the randomisation numbers of site 1 will be 1101, 1102,…, 1201, 1202,…, 1301, 1302,…, while the randomisation numbers of site 2 will be 2101, 2102,…, 2201, 2202,…, 2301, 2302,… .
The kit number will be a unique progressive 3-digit number with the capital letter "K" as prefix (i.e. K101, K102, K103 … for cohort 1, K201, K202, K203 … for cohort 2 and K301, K302, K303 …. for cohort 3).

## 8.2 Treatment allocation

Subjects will be assigned to receive active or placebo product within each cohort according to the randomisation list.
An IWRS system will assign a randomisation number and a kit number to each randomised subject on the basis of their treatment arm and on the basis of the kits available at the clinical centre.

## 8.3 Blinding

The study will be double-blind within each cohort, i.e. all study subjects, site personnel, monitors, sponsor, contract research organization (CRO) study teams and DSMB will be blinded to the treatment given to each subject.

### *8.3.1 Emergency code and unblinding procedures*

Unblinding of the code for specific subjects will be fully documented in the source documents, in the eCRF and in the clinical study report.

Breaking of an individual randomisation code by the investigator during the study is allowed only when knowledge of the code is essential for the subject's health. The investigator will break the blind for a specific subjects by accessing the eCRF system and requesting the system to break the blind. The system will send an email to the investigator with a one-time password to access the blinded code. A separate audit trail will track any unblinding step.

The date and the reason for breaking the code must be recorded in the eCRF.

# 9 EVALUATION PARAMETERS

## 9.1 Study variables

### *9.1.1 Primary variables*

- Proportion of subjects with severe / serious treatment-emergent adverse events (TEAEs) (including clinically relevant laboratory abnormalities and vital signs and adverse reactions at the injection site) in the 7 days post-treatment.

### *9.1.2 Secondary variables*

- Proportion of subjects with any unsolicited and solicited TEAEs (including clinically relevant laboratory abnormalities, vital signs and adverse reactions at the injection site) up to each assessment time and throughout the study
- MAD0004J08 serum concentrations and PK parameters $C_{max}$, $t_{max}$, $AUC_{0-t}$, $AUC_{0-\infty}$, $t_{1/2}$, $Cl_t/F$ and $V_z/F$ after single dose of 48 mg, 100 mg and 400 mg
- Number and percentage of ADA positive subjects and mean maximum ADA concentrations after single dose of 48 mg, 100 and 400 mg MAD0004J08
- Serum neutralising power at baseline, 48 h post-dose, on day 8, 1 month (day 30±3), 4 months (day 120±4), after single dose of 48 mg, 100 mg and 400 mg MAD0004J08

### *9.1.3 Exploratory variable*

- Characterisation of DIESSE ELISA kit on serum samples (baseline, 48 h post-dose, on day 8, at 1 month (day 30±3), 4 months (day 120±4) and 6 months (180±7)
- IgG and neutralizing power in saliva samples at 6 months (180±7)

## 9.2 Pharmacokinetic and ADA assessments

### *9.2.1 Pharmacokinetic parameters*

The following PK parameters will be measured and/or calculated for MAD0004J08, using the validated software Phoenix WinNonlin® version 6.3 (34) or higher (actual version will be stated in the final report):

| | |
|---|---|
| $C_{max}$: | Maximum serum concentration |
| $t_{max}$: | Time to achieve $C_{max}$ |
| $\lambda_z$: | Terminal elimination rate constant, calculated, if feasible, by log-linear regression using at least 3 points |
| $t_{1/2}$: | Half-life, calculated, if feasible, as $\ln2/\lambda_z$ |
| $AUC_{0-t}$: | Area under the concentration-time curve from administration to the last |

observed concentration time t, calculated with the linear trapezoidal method

$AUC_{0-\infty}$: Area under the concentration-time curve extrapolated to infinity, calculated, if feasible, as $AUC_{0-t}$ + $C_t/\lambda_z$, where $C_t$ is the last measurable drug concentration

$Cl_t/F$ Total body clearance, calculated, if feasible, as Dose/ $AUC_{0-\infty}$

$V_z/F$ Volume of distribution associated with the terminal slope, calculated, if feasible, as Dose/($AUC_{0-\infty}$*$k_{el}$)

The sampling schedule is considered adequate if the ratio $AUC_{0-t}/AUC_{0-\infty}$ equals or exceeds a factor of 0.8 (i.e. if %$AUC_{extra}$ is <20%) for more than 80% of the individual PK profiles. This assures that $AUC_{0-t}$ covers a sufficient percentage of the theoretical total extent of exposure.

The quality of log-linear regression (and, consequently, the reliability of the extrapolated PK parameters) should be demonstrated by a determination coefficient $R^2 \geq 0.8$. Individual extrapolated parameters, when considered unreliable, will be reported as NC (not calculated).

## 9.3 Safety assessments

Safety and general tolerability of the investigational products will be based on TEAEs (unsolicited and solicited), adverse reactions at the injection site, physical examinations including body weight, vital signs, ECG and routine haematology, blood chemistry and urinalysis laboratory tests. For more details please refer to § 7.1, 7.2, 7.3, 7.4.

# 10 STATISTICAL METHODS

The data documented in this study and the parameters measured will be evaluated and compared using classic descriptive statistics, i.e. geometric mean (PK data only), arithmetic mean, SD, CV (%), minimum, median and maximum values for quantitative variables, and frequencies for qualitative variables.

A Statistical Analysis Plan will be prepared by CROSS Research S.A. Biometry Unit, approved by the Sponsor and finalised before database lock.

Not available data will be evaluated as "missing values".
Safety, immunogenicity, neutralising power and demography data will be analysed using SAS® version 9.3 (TS1M1) (34) or higher (the actual versions will be stated in the final report).
Serum concentrations and PK parameters will be calculated using Phoenix WinNonlin® version 6.3 (34) or higher and SAS® version 9.3 (TS1M1) or higher.

## 10.1 Analysis Sets

### *10.1.1 Definitions*

A subject will be defined as screened after the signature of the informed consent, regardless of the completion of all the screening procedures.

A subject will be defined as eligible if he/she meets all the inclusion/exclusion criteria. Otherwise he/she will be defined as a screen failure.

A subject will be defined as randomised in the study when he/she is assigned to a randomised treatment arm.
An eligible but not randomised subject will be defined as a reserve.

The following analysis sets will be defined:

- Randomised set: all randomised subjects. This analysis set will be used for demographic, baseline and background characteristics.
- Safety set: all subjects who receive a dose (at least one injection) of investigational product. This analysis set will be used for the safety analyses.
- PK set: all randomised subjects who fulfil the study protocol requirements in terms of investigational product administration and have evaluable PK data readouts, with no major deviations that may affect the PK results.
- ADA set: all randomised subjects who fulfil the study protocol requirements in terms of investigational product administration and have evaluable ADA data readouts, with no major deviations that may affect the results.
- Serum Neutralising power set: all randomised subjects who fulfil the study protocol requirements in terms of investigational product administration and have evaluable

Serum Neutralising power data readouts, with no major deviations that may affect the results.

- Saliva analysis set: all randomised subjects who fulfil the study protocol requirements in terms of investigational product administration and have evaluable saliva analysis data readouts, with no major deviations that may affect the results.

Each subject will be coded by the CRO Biometry Unit as valid or not valid for the Safety set, the PK set and the ADA set and Serum Neutralising power set. Subjects will be evaluated according to the treatment they actually receive.

### *10.1.2 Reasons for exclusion from the PK or ADA set or Serum Neutralising power set or saliva analysis*

Reasons for the exclusion of subjects from the PK or ADA or Serum Neutralising power set before bioanalysis are the following:

- intake of concomitant medications which could render the mAb serum concentration-time profile or ADA presence or Serum Neutralising power or saliva analysis results unreliable
- occurrence of AEs which could render the mAb serum concentration-time profile or ADA presence or Serum Neutralising power or saliva analysis results unreliable
- administration errors which could render the mAb serum concentration-time profile or ADA presence or Serum Neutralising power or saliva analysis results unreliable
- other events which could render the mAb serum concentration-time profile or ADA presence or Serum Neutralising power or saliva analysis results unreliable
- PK only: positive result at SARS CoV-2 test during the study

If one of these events occurs, it will be noted in the eCRF as the study is being conducted.

## 10.2 Sample size and power considerations

A total of 30 healthy men and women (10 in each cohort) will be enrolled in the study.

## 10.3 Demographic, baseline and background characteristics

Demographic data (such as age, gender, race, BMI) and other relevant baseline characteristics (such as intake of previous medications and presence of previous diseases) will be summarised according to qualitative or quantitative data. Qualitative data will be summarised in contingency tables. Quantitative data will be summarised using classic descriptive statistics.

## 10.4 Analysis of safety parameters

At the end of the study, after database lock and treatments unblinding, all safety data will be analysed overall and by treatment group, within each cohort.

Additionally, all safety data (all TEAEs including clinically significant laboratory abnormalities, clinically significant vital signs results and adverse reactions at the injection site) collected up to the completion of Visit 5 (48 h) assessments by the last subjects in Cohort 3 will be analysed in blind conditions. Summary tables will be generated and sent to the DSMB/Sponsor before start of the Phase II trial planned by the Sponsor.

#### *10.4.1 Safety and tolerability evaluation*

- **AEs**

AEs will be coded by System Organ Class (SOC) and Preferred Term (PT), using the most current version of the Medical Dictionary for Regulatory Activities (MedDRA).
AEs will be classified as pre-treatment AEs (PTAEs) and treatment-emergent AEs (TEAEs), according to the period of occurrence, as follows:

- PTAEs: all AEs occurring after signing the inform consent and before the first dose of IMP and not worsening after the first dose of IMP
- TEAEs: all AEs occurring or worsening after the first dose of IMP

Individual PTAEs and TEAEs will be listed in subject data listings. No summary table will be provided for PTAEs. TEAEs will be summarised by treatment and overall.

The number and percentage of subjects with any TEAE and the number of TEAEs will be tabulated by SOC and PT, as unsolicited/solicited, seriousness, relationship to treatment and severity. For TEAEs that change severity during the study (e.g. from mild to moderate or from moderate to or severe), the more severe intensity will be reported in the summary tables.

The Investigator will evaluate the reasonable possibility of a causal relationship with the IMP and any other causal relationship.

TEAEs (including clinically significant laboratory parameters abnormalities, vital signs and adverse reactions at the injection site), as detailed in § 3.1.2, up to 48 h post-dose for Cohort 1 and then for Cohort 2 subjects will be listed and provided to the DSMB for safety evaluation.

- **Physical examination**

Clinical significant abnormal findings after enrolment will be reported as AEs and listed in the clinical study report. Date of the physical examination, overall investigator's interpretation (as normal or abnormal and, if abnormal, clinically significant or not clinically significant) and clinically significant abnormalities (if any) will be listed.

- **Laboratory data**

Clinical significant laboratory parameters after enrolment will be reported as AEs and listed in the clinical study report. Laboratory parameter values will be listed and summarised by presenting a table of subjects with abnormalities, the descriptive statistics of parameter values and their changes from baseline (n, mean, SD, CV%, min, median and max) for quantitative parameters or tables of frequencies for qualitative parameters and shift tables.

- **Vital signs**

Clinical significant abnormal results will be reported as AEs and listed in the clinical study report. Vital signs values will be listed and summarised by descriptive statistics.

➢ **Body weight**

Values of body weight will be listed and summarised by descriptive statistics.

➢ **ECG**

Clinical significant abnormal ECG results will be reported as AEs and listed in the clinical study report.

### *10.4.2 Primary safety analysis*

Number and proportion of subjects with severe TEAEs and of subjects with SAEs in the 7 days post-dose will be summarised in contingency tables overall and by treatment, within each cohort. Proportions will be compared between treatments and placebo (within cohort 1, D1 vs. Placebo and within cohort 2, D2 vs. Placebo) by means of Fisher's exact test for small proportions.

### *10.4.3 Secondary safety analyses*

Number and proportion of subjects with TEAEs up to each assessment time and throughout the study will be summarised in contingency tables overall and by treatment, within each cohort.

Solicited and unsolicited AEs will be analysed separately.

## 10.5 Analysis of pharmacokinetic parameters

A descriptive PK will be presented. Concentration results will be listed by subject and summarised in tables and figures. Individual and mean curves (+SD at sampling times), indicating inter-subject variability, will be plotted. Data below the lower quantification limit (BLQL) will be considered as 0 in the calculations and presented as BLQL in listings and tables.
PK parameters will be listed and summarised by descriptive statistics. As a consequence of BLQL (i.e. 0) values, calculated geometric means (if requested) could be null. For this reason, in the presence of any null value, the geometric mean will be reported as not calculated (NC).

## 10.6 Analysis of ADA

ADA concentrations will be listed by subject and summarised by descriptive statistics. BLQL data will be presented as BLQL in listings and tables.

Individual maximum ADA concentrations will be listed and mean maximum concentration presented.

Number and percentage of ADA-positive subjects will be listed and summarised by assessment time and overall.

## 10.7 Interim analysis of PK data

Serum PK data for all subjects (cohort 1, cohort 2 and cohort 3) up to day 8 and up to day 30 (both inclusive) will be analysed under blind conditions.

PK concentration results will be listed by masked subject, i.e. a masked code will be used for each subject in order to maintain the blinding conditions. Data below the lower quantification limit (BLQL) will be presented as BLQL in the listings.

The masked code will be automatically generated, will be different from the subject identification code and will not allow subject or group identification.

## 10.8 Serum neutralising power test results

Serum neutralising power data for all subjects (cohort 1, cohort 2 and cohort 3) up to day 8 and up to day 30 (both inclusive) will be sent under blind conditions from the analytical laboratory to the CRO before study end. Results will be listed by the analytical laboratory by masked subject, i.e. a masked code will be used for each subject in order to maintain the blinding conditions. The masked code will be different from the subject identification code and will not allow subject or group identification.
Overall, at final analysis serum neutralising power results will be listed by subject and assessment time-point and summarised by descriptive statistics.

## 10.9 DIESSE Elisa kit characterisation

DIESSE kit characterisation data for all subjects (cohort 1, cohort 2 and cohort 3) up to day 8 (inclusive) will be sent under blind conditions from the analytical laboratory to the Sponsor before study end. Results will be listed by the analytical laboratory by masked subject, i.e. a masked code will be used for each subject in order to maintain the blinding conditions. The masked code will be different from the subject identification code and will not allow subject or group identification.
Overall results will be listed as applicable.

## 10.10 Saliva samples' analysis results

Saliva IgG and neutralising power data for all subjects (cohort 1, cohort 2 and cohort 3) - visit 12 (month 6) will be sent from the analytical laboratory to the CRO. Overall results will be listed and summarised as applicable.

# 11 DEFINITION AND HANDLING OF AEs AND SAEs

Adverse events (AEs) will be assessed throughout the study from the signature of the informed consent up to the end of the study (6 months; day 180±7) as follows:

- **Solicited local and systemic AEs** will be recorded and assessed for severity by the participant from day 1 to **7 days** after study drug administration (i.e. day 8) using pre-printed forms in the diary provided to the participant. For solicited AEs please refer to § 7.3.1 and 7.3.2. Assessment of seriousness will be carried out by the investigator as described in this section.

- **Unsolicited AEs** will be recorded and assessed by investigators after study drug administration based on study visits, free text diary entries (diary 2 from day 1 to Visit 9), and ad-hoc contacts by the participant.

- **All serious AEs (SAEs)** will be recorded and assessed by investigators **throughout the study** based on study visits, free text diary entries (for the 1$^{st}$ month), and ad-hoc contacts by the participant.

## 11.1 Applicable SOPs

AEs definition, classification and management will follow the SOP of CROSS Research S.A., based upon applicable local and international regulations. The full SOP or an operative summary will be made available to the clinical centres.

A brief summary of AE definition, classification and management is reported below.

## 11.2 Definitions

➢ **Adverse event (AE)**
Any untoward medical occurrence in a subject or clinical investigation subject administered a pharmaceutical product and which does not necessarily have to have a causal relationship with treatment.

➢ **Adverse Drug Reaction (ADR)**
Any noxious and unintended response to a medicinal product for which a causal relationship between a medicinal product and an AE is at least reasonably possible in the investigator's or sponsor's opinion, the relationship cannot be ruled out (see § 11.5) resulting not only from the authorised use of a medicinal product at normal doses, but also from medication errors and uses outside the terms of the marketing authorisation, including the misuse and abuse of the medicinal product.

➢ **Pre-treatment AE (PTAE)**
Any AE occurring before the first dose of a medicinal product and not worsening after the first dose. The following medical occurrences and clinical investigations are the only clinically significant events which, according to the investigator judgement, can be defined and recorded as PTAEs:

- trauma (fractures, sprains, strains, falls, domestic accidents, car accidents, etc.) occurred after the signature of the informed consent and before the first medicinal product administration
- new measurements (vital signs, laboratory parameters, etc.), performed after the signature of the informed consent and before the first medicinal product administration, which show a clinically significant worsening in comparison with a previous (baseline) measurement performed after the signature of the informed consent
- any disease diagnosed after the anamnesis recorded at visit 1 and before the first medicinal product administration
- physical and mental status changes (pre-syncope, anxiety, dizziness, fainting, etc.) occurred after the signature of the informed consent and before the first medicinal product administration

- **Treatment-emergent AE (TEAE)**

Any AE occurring or worsening after the first dose of a medicinal product

- **Serious Adverse Event (SAE)**

Any untoward medical occurrence that at any dose:

- results in death
- is life-threatening
- requires inpatient hospitalisation or prolongation of existing hospitalisation
- results in persistent or significant disability/incapacity
- is a congenital anomaly/birth defect
- is an important medical event that may jeopardize the subject’s health status or may require intervention to prevent one of the other outcomes listed in the definition above. Examples of such events are cancer, intensive treatment in an emergency room or at home for allergic bronchospasm; blood dyscrasias or convulsions that do not result in hospitalisation; or development of drug dependency or drug abuse

- **Unexpected ADR**: an ADR the nature or severity of which is not consistent with the Reference Safety Information (RSI)

- **Reference Safety Information (RSI)**: in order to assess whether an adverse reaction is expected, the Investigator’s Brochure (IB) will be used for the IMP.

- **Suspected Unexpected Serious Adverse Reaction (SUSAR)**

An ADR that is both unexpected (not consistent with the RSI) and also meets the definition of a SAE.

## 11.3 AEs monitoring window

- Start of monitoring: from immediately after the signature of the informed consent
- End of monitoring: last follow-up visit/ETV

An AE occurring after the last follow-up visit/ETV and coming to knowledge of the investigator (e.g. by spontaneous reporting by study subjects) must be recorded only if it is an ADR, according to the investigator's judgment.
See § 11 above for recording windows of different categories of AEs.

## 11.4 AEs recording

All AEs derived by spontaneous, unsolicited reports of the subjects, by observation and by routine open questioning should be collected and reported.
The following minimal information will be recorded for an AE (detailed explanation for each element is available in the SOP or in the operative summary made available to the clinical centre) in the source documents and later transcribed into the eCRF:

1. Adverse Event: progressive number of the adverse event
2. Description: verbatim description of the adverse event or
   Follow-up: progressive number of follow-up of the adverse event
3. Acknowledgment Date/Time: acknowledgment date/time of the adverse event or
   Follow-up Date/Time: follow-up date/time of the adverse event
4. Start Date/Time: start date/time of the adverse event
5. End Date/Time: end date/time of the adverse event
6. Affected Body Area: anatomical location relevant for the event
7. Whether the adverse event start before or after the first intake of the study drug or whether the adverse event has worsened or not after the first intake of the study drug
8. Last Study Drug Administration Date/Time Before Onset: if the adverse event started after the first administration of the study drug, the date/time of last administration of the study drug before the onset of the adverse event or
   Last Study Drug Administration Date/Time Before Worsening: In case of treatment emergent adverse event, the date/time of the last administration of the study drug(s) before the worsening of the adverse event.
9. Investigator's opinion about the reasonable possibility of a causal relationship with the study drug (see § 11.5).
10. Investigator's opinion about other causal relationship (e.g. non study drug, concomitant therapy, study device, etc.).
11. Severity: the severity or intensity of the event
    - 1 Mild
    - 2 Moderate
    - 3 Severe

The investigator will use the terms MILD, MODERATE, SEVERE to describe the maximum intensity of the AE. For purposes of consistency, these intensity grades are defined as follows:

| Grade | Intensity | Description |
|---|---|---|
| 1 | MILD | Does not interfere with participant's usual function. |
| 2 | MODERATE | Interferes to some extent with participant's usual function. |
| 3 | SEVERE | Interferes significantly with participant's usual function. |

For severity grading of safety laboratory parameters (haematology and biochemistry alterations) the Common Terminology Criteria for Adverse Events (CTCAE) version 5.0 may be consulted. The CTCAE criteria should be considered as a guidance and are not binding.

**12.** Pattern: Used to indicate the pattern of the event over time

- 1 Single Event
- 2 Continuous
- 3 Intermittent

**13.** Serious Adverse Event

**14.** Action Taken with Study Drug: describes changes to the study drug as a result of the event. It is specifically for actions taken with the study drug

- 1 Dose Not Changed
- 2 Dose Increased
- 3 Dose Reduced
- 4 Drug Interrupted (i.e. temporary stop)
- 5 Drug Withdrawn (i.e. definitive stop)
- 6 Not Applicable (e.g. drug administration not started yet or completed)
- 7 Unknown

**15.** Concomitant Therapy: if a concomitant therapy is given, it must be reported in the specific eCRF forms

**16.** Study Discontinuation: if the adverse event cause the subject to be discontinued from the study

**17.** Other Action Taken: other actions taken as a result of the event that are unrelated to dose adjustments of study drug

**18.** Outcome: Outcome of the event

- 1 Recovered/Resolved
- 2 Recovered/Resolved With Sequelae
- 3 Recovering/Resolving
- 4 Not Recovered/Not Resolved
- 5 Fatal
- 6 Unknown

### 11.5 Guidance for assessing relationship of AE with investigational product

According to the CRO SOP and to the EU Detailed guidance on the collection, verification and presentation of adverse event/reaction reports arising from clinical trials on medicinal products for human use ('CT-3'), the causality assessment will be determined using a two-level scale: related or not related.

An AE/SAE is considered related to study intervention if there is a reasonable possibility that the study intervention contributed to the AE. Not-related means there is no reasonable possibility that the AE is causally related to administration of the study intervention. There are other more likely causes for the AE.

In details:

| **Assessment of causality** |
|---|
| • The investigator will assess the relationship between study intervention and each occurrence of each AE/SAE.<br>• A "reasonable possibility" of a relationship conveys that there are facts, evidence, and/or arguments to suggest a causal relationship, rather than a relationship cannot be ruled out.<br>• The investigator will use clinical judgment to determine the relationship.<br>• Alternative causes, such as underlying disease(s), concomitant therapy, and other risk factors, as well as the temporal relationship of the event to study intervention administration, will be considered and investigated.<br>• The investigator will also consult the Investigator's Brochure (IB) in his/her assessment.<br>• For each AE/SAE, the investigator must document in the medical notes that he/she has reviewed the AE/SAE and has provided an assessment of causality.<br>• There may be situations in which an SAE has occurred and the investigator has minimal information to include in the initial report to the sponsor. However, it is very important that the investigator always make an assessment of causality for every event before the initial transmission of the SAE data to the sponsor.<br>• The investigator may change his/her opinion of causality in light of follow-up information and send an SAE follow-up report with the updated causality assessment.<br>• The causality assessment is one of the criteria used when determining regulatory reporting requirements.<br>• If the investigator does not know whether or not the study intervention caused the event, then the event will be handled as "related to study intervention" for reporting purposes, as defined by the sponsor. In addition, if the investigator determines that an SAE is associated with study procedures, the investigator must record this causal relationship in the source documents and CRF, and report such an assessment in the dedicated section of the SAE Report Form and in accordance with the SAE reporting requirements. |

## 11.6 SAEs reporting

**IMPORTANT:** National regulations and requirements for SAE reporting **prevail**, however, in general, the following indications should be followed.

The investigator must report any SAE within 24 h of becoming aware of the event, and send a completed SAE form to the CRO responsible for Pharmacovigilance (§ 11.10). The Pharmacovigilance CRO will report all SAEs to the Sponsor, to CROSS Research SA and to the local CROs.

The investigator shall notify the competent Ethics Committee (EC) within 7 days of any SAE with lethal outcome occurred during a study. If the investigator is initially unable to obtain all the necessary details for completing the SAE form, he/she should in any case transmit all the available information. The investigator should provide an appropriate follow-up of SAEs to all concerned parties.
Seriousness and causality must be assessed by the investigator. Expectedness is usually assessed by the sponsor.
If the investigator is unable to assess the causality it is recommended to adopt a conservative approach and treat the event as a suspected adverse reaction until follow-up information is available.
The sponsor may also make an assessment of causality, independent of that of the investigator. The most conservative approach should be taken when it comes to regulatory reporting. Under no circumstances should the sponsor downgrade the investigator's opinion or put the investigator under pressure to change his/her assessment. In case of disagreement, both the opinion of the investigator and the sponsor should be provided in the report.
The sponsor will evaluate the SAE expectedness on the basis of the RSI.

The Pharmacovigilance CRO should track the SAE occurred during a study, for example, using a spreadsheet to ensure that missing SAE reports are requested in a timely manner. It is also recommended that a record of follow-up attempts is maintained as, in the event of the information not being received, this would demonstrate due Pharmacovigilance CRO diligence.

## 11.7 SUSARs management

The clock for initial expedited reporting starts as soon as the information containing the minimum reporting criteria has been received by the sponsor (day 0).
For fatal and life-threatening SUSARs the EC and Competent Authority (CA) should be informed as soon as possible and in any case within 7 days.

If the initial report is incomplete, e.g. not all the information/assessments were available, a complete report should be sent within an additional 8 days.

SUSARs which are not fatal and not life-threatening are to be reported within 15 days.
The minimum information to be reported includes:

- Sponsor study number
- One identifiable coded subject
- One identifiable reporter

- One SUSAR
- One suspect IMP (including active substance name, code)
- A causality assessment (a reasonable possibility of a causal relationship with the study drug can be excluded only if there is information supporting this decision, otherwise it cannot be excluded).

## 11.8 Other events qualified for expedited reporting

Other safety issues also qualify for expedited reporting when they might materially alter the current benefit-risk assessment of a medicinal product or would be sufficient to consider changes in the medicinal product administration or in the overall conduct of the trial, for instance:

- single case reports of an expected serious adverse reaction with an unexpected outcome (e.g.: a fatal outcome)
- an increase in the rate of occurrence of an expected serious adverse reaction, which is judged to be clinically important.
- post-study SUSARs that occur after the subject has completed a clinical trial and are reported to the investigator by the subject.
- new events relating to the conduct of the trial or the development of the medicinal product likely to affect the safety of the subjects, such as :
  - a SAE which could be associated with the trial procedures and which could modify the conduct of the trial
  - a significant hazard to the subject population such as lack of efficacy of a medicinal product used for the treatment of a life-threatening disease
  - a major safety finding from a newly completed animal study (such as carcinogenicity) or from other clinical trials.

## 11.9 Pregnancy

Women will be instructed to notify the investigator immediately if they become pregnant during the study. Pregnant subjects will be withdrawn from the study. The subjects will also be instructed to report pregnancies discovered after the last visit, if they believe that conception occurred during their participation in the study.
The investigator should report pregnancies according to the procedures and timelines described for reporting of SAEs (see above).
The pregnant subject will be followed until the end of the pregnancy. Any complication during the pregnancy should preferably be reported as an AE. Also the outcome of the pregnancy must be reported on a specific report. Any spontaneous abortion, stillbirth, birth defect/congenital anomaly, death, or other serious infant condition must be reported and followed up as a SAE.

## 11.10 SAEs: contacts

SeQure Srl, Via Roveggia, 122 – 37136 Verona, Italy

Email: safety@sequrelifesciences.com

**Main Contact**
Alessandra Marchese, Drug Safety Manager & GVP Consultant
Email: alessandra.marchese@sequrelifesciences.com
Phone: +39.069.291.9456

# 12 DATA MANAGEMENT PROCEDURES

## 12.1 Data collection – eCRFs

The investigator must ensure that the clinical data required by the study protocol are carefully reported in the eCRFs, including data inserted by the subjects in the study diary. He/she must also check that the data reported in the eCRFs correspond to those in the subject's source documents, including subject's diary data. Data collected up to 48h (for Cohorts 1, 2 and 3) (all subjects) must be reported in the eCRF within 24h from the collection.

## 12.2 Unique subject identifier

All the subjects who sign the informed consent form for the present study will be coded with "unique subject identifiers" when data are extracted from the study database into the domains of the CDISC SDTM model.

Screening numbers will be 4 digits numbers with the structure [CCC]/S[NNN], where [CCC] (001, 002) indicates the centre and [NNN] is a progressive number within the centre (e.g. 001/S001, 001/S002… for site number 1, 002/S001, 002/S002 for site number 2…).

The unique subject identifier consists of the sponsor study code (i.e. A0001A), the 3-digit site number (i.e. 001 or 002), the 4-digit screening number ( see above) and the 4-digit subject randomisation number (see §8.1). Study code, site number, screening number and subject randomisation number are separated by slashes ("/").

The last 12 digits of the unique subject identifier (enrolled subjects), corresponding to the centre, subject screening and subject randomisation numbers separated by a slash, or the last 8 digits of the unique subject identifier (not enrolled subjects), corresponding to the subject centre and screening numbers separated by a slash, will appear as subject identifier in the individual listings and figures of the clinical study report.

## 12.3 Database management

Database and eCRF design will be set-up by the eCRF provider (§ 16.11) before the start of the study. Verification and cross-verification of the data entered by the study personnel will be automatically performed by the system, according to predefined nature/type of the data, value ranges and rules. All rules applied by the system to check specific items will be detailed in the Data Validation Plan (DVP). Queries will be automatically generated by the system for all data found to be inconsistent, incorrect or missing. All changes performed by the Investigators/authorized users will be tracked by the system.

Once all data are entered in the eCRF and all outstanding queries are solved and the Monitors complete the Source Data Verification (SDV), the Data Manager will verify the correctness of the answers and the Investigators will approve (sign) the data of the subjects' eCRF.

Data management standards and procedures will be stated in the Data Management Plan in agreement with the Sponsor.

### *12.3.1 SDTM and ADaM*

The Biometry Unit of CROSS Research will extract data from the eCRF and will provide tabulation datasets according the SDTM model of CDISC and analysis datasets according to the ADAM model of CDISC. Rules for creating tabulation datasets will be defined in the DMP together with annotated eCRF. Quality check (QC) will be performed before issuing final domains. SDTM and ADaM domains will be validated using the Pinnacle 21 Community validator. The final data file will be transferred to the sponsor in the agreed format with all the other study documentation.

### *12.3.2 Coding dictionaries*

Medical/surgical history and underlying diseases, clinically significant physical examination abnormalities and AEs will be coded using the most current version of Medical Dictionary for Regulatory Activities (MedDRA™).
Previous and concomitant medications will be coded using the most current version of WHO Drug Dictionary Enhanced (WHODDE). The actual version of the coding dictionaries will be stated in the study report.

# 13 STUDY MONITORING, QUALITY CONTROL AND QUALITY ASSURANCE

## 13.1 Monitoring

The monitoring visits will be conducted by the appointed CRAs (§ 16.9).

Monitoring activities, including monitoring purpose, selection and qualifications of monitors, extent and nature of monitoring, monitoring procedures, monitoring reports will comply with ICH-GCP chapter 5.18 requirements. Monitoring activities will be detailed in the monitoring plan.

Adequate time and availability for monitoring activities should be ensured by the investigator and key study personnel.

Data verification is required and will be done by direct comparison with source documents, always giving due consideration to data protection and medical confidentiality. In this respect the investigator will assure support to the monitor at all times. If routine monitoring site visits are not possible during COVID-19 infection public health crisis, off-site monitoring activities (e.g. phone calls, video visits, emails or other remote tools in order to discuss the trial with the investigator and review the data) could be implemented after a suitable risk evaluation. These could include remote source data verification (rSDV) only when considered necessary and in line with the current national law (or temporary national emergency measures). Details will be reported in the Monitoring Plan and in the final clinical study report.

The investigator agrees, by written consent to this protocol, to fully co-operate with compliance checks by allowing authorised individuals to have access to all the study documentation. In addition to the monitoring activities performed by the study monitor, the sponsor could perform some quality control activities to verify the compliance with the study procedures and the ICH-GCP guidelines.

## 13.2 Quality Control and Quality Assurance

The CRO has implemented and maintains a Quality System that includes quality controls and audits at different study steps with written SOPs to ensure that the study is conducted in compliance with the protocol and all effective amendments, ICH-GCP, and the applicable regulatory requirement(s) and that data have been reliably and correctly generated, recorded, processed and reported, in agreement with the ALCOAC principles (Attributable-Legible-Contemporaneous-Original-Accurate-Complete).

The clinical sites are responsible for implementing and maintaining quality assurance and a quality control system to ensure that the study is conducted and data are generated, documented (recorded), and reported in compliance with the protocol, ICH-GCP, and the applicable regulatory requirement(s).

The CROs and the sponsor will be responsible for their respective activities.

The sponsor may transfer any or all of the sponsor's trial-related duties and functions to a CRO, but the ultimate responsibility for the quality and integrity of the trial data always resides with the sponsor.

## 13.3 Applicable SOPs

The sponsor, the clinical centres, the pharmacovigilance provider and the CRO will follow their respective SOPs in the conduct of the respective activities, unless otherwise stated in written agreements. SOPs will be made available for review, if required. AEs definition, classification and management will follow the SOP of CROSS Research S.A.

## 13.4 Data access

The investigator and the CRO will ensure that all raw data records, medical records, eCRF and all other documentation that is relevant to this study will be made accessible for monitoring activities, audits, IEC review, and regulatory inspections.

## 13.5 Audits and inspections

The sponsors, independent bodies acting on behalf of the sponsor and the CRO have the right to perform audits according to ICH-GCP responsibilities.

The study may also be inspected by regulatory authorities.

The Investigators and the CRO agree, by written consent to this protocol, to fully co-operate and support audits and inspections compliance checks by allowing authorised individuals to have access to all the study documentation.

# 14 ETHICAL CONSIDERATIONS

## 14.1 Ethics and Good Clinical Practice (GCP)

The study will be performed in accordance with the relevant guidelines of the Declaration of Helsinki.
The approval and/or the acknowledgment of the study protocol, the Investigator's brochure and all other relevant documentation by the National Competent Authorities, central Ethics Committees and local Ethics Committees competent for each study site will be obtained before the start of the study, according to the current regulations.

The present clinical study will be carried out according to the current revision of Good Clinical Practice (GCP), ICH topic E6 (R2), and the applicable local law requirements.

## 14.2 Informed consent

Before being enrolled into the clinical study, the subjects must have expressed their consent to participate, after the investigator has explained to them, clearly and in details, the scope, the procedures and the possible consequences of the clinical study. Information will be given in both oral and written form. The information sheet and informed consent form will be prepared in the local language and must be approved by the EC and regulatory authorities. It will include all the elements required by law according to the ICH-GCP recommendations.
In addition to the standard requirements that physicians are currently obliged to observe when providing information, the following points must also be covered:

- a description of the aims of the study and how it will be organised
- the type of treatment (information on the IMP(s) and treatment procedures, as applicable)
- any potential negative effects attributable to the study product or treatment
- the freedom to ask for further information at any time
- the subjects' right to withdraw from the clinical study at any time without giving reasons and without jeopardising their further course of medical treatment
- the existence of a subject insurance cover and obligations following from this cover

Adequate time and opportunity to satisfy questions will be given to the subjects and the time will be recorded.

The investigator will be supplied with an adequate number of blank informed consent forms to be used. The forms will be signed and dated by both the investigator and the subject.
A copy of the signed form will be given to the subject.

To ensure medical confidentiality and data protection, the signed informed consent forms will be stored in the investigator's study file according to the regulatory requirements (see § 15.3). The investigator will allow inspection of the forms by authorised representatives of the sponsor, EC members and regulatory authorities. He/She will confirm, by signing and dating the forms, that informed consent has been obtained.

## 14.3 Insurance policy

An insurance cover has been issued in favour of the subjects participating in this clinical study. The insurance is in compliance with the local regulations and with the requirements of the Health Authorities.

## 14.4 Withdrawal of subjects

It will be documented whether or not each subject completed the clinical study. If, for a subject, study treatment or observations are discontinued, the type of discontinuation and the primary reason for discontinuation will be recorded.

### *14.4.1 Discontinuation procedures*

For any subject discontinuing, the investigator will:

- ask the subject to undergo, as far as possible, a final medical visit (ETV) to examine the subject's health conditions
- arrange for alternative medical care of the withdrawn subject, if necessary
- report in the eCRF date and time of the investigational product administration, and date and primary reason of study discontinuation
- record in the eCRF any follow-up, if the subject is withdrawn for an AE

Discontinued subjects will not be replaced.

## 14.5 Primary reason for subject's discontinuation

- **Adverse event**: Any significant AE that in the opinion of the investigator or concerned subject is not compatible with study continuation. For the definition of AE, please refer to § 11.2.
- **death**: the absence of life or state of being dead
- **lost to follow-up**: the loss or lack of continuation of a subject to follow-up
- **physician decision**: a position, opinion or judgment reached after consideration by a physician with reference to the subject
- **pregnancy**: pregnancy is the state or condition of having a developing embryo or fetus in the body (uterus), after union of an ovum and spermatozoon, during the period from conception to birth
- **protocol deviation**: an event or decision that stands in contrast to the guidelines set out by the protocol
- **withdrawal by subject**: study discontinuation requested by a subject for whatever reason
- **other**: different than the ones previously specified

### 14.6 Study termination

The study will be considered terminated at the date of LSLV. The investigator and the sponsor have the right to discontinue the study at any time for reasonable medical and/or administrative reasons. As far as possible, this should occur after mutual consultation.

Primary reasons for study discontinuation are, but not limited to, the following:

- **site terminated by sponsor**: an indication that a clinical study was stopped at a particular site by its sponsor
- **study terminated by sponsor**: an indication that a clinical study was stopped by its sponsor
- **technical problems**: a problem with some technical aspect of a clinical study, usually related to an instrument

Reasons for discontinuation have to be documented appropriately.

### 14.7 Holding rules

Holding rules are met in the following circumstances:

1. If 2 or more volunteers in a cohort present with the same severe solicited local adverse event beginning within 2 days after drug administration and persisting as severe for >48h
2. If 2 or more volunteers in a cohort present with the same severe solicited systemic adverse event beginning within 2 days drug administration and persisting as severe for >48h
3. If 2 or more volunteers in a cohort present with the same severe unsolicited adverse event (including the same laboratory adverse event or same alteration of vital parameters or same reported symptoms) that is related drug administration and persists as severe for > 48hrs
4. A single serious adverse events that is considered related to the drug administration

If a holding rule is met the Sponsor will inform the regulatory authority within 24 hours.

Besides, the DSMB and Ethics committee will be asked if it is deemed appropriate to restart dosing after an interim analysis on safety data.
Internal safety review will consider:

1. The relationship of the AE or SAE to the active arm.
2. The relationship of the AE or SAE to the drug dose, or other possible causes of the event.
3. If appropriate, additional screening or laboratory testing for other volunteers to identify those who may develop similar symptoms and alterations

4. New, relevant safety information from ongoing research programs on the various components of the mAb if they are available

The sponsor, national ethics committee and DSMB will also be notified if a holding rule is activated or released and if the trial has been early terminated for safety reasons.

# 15 ADMINISTRATIVE PROCEDURES

## 15.1 Material supplied to the clinical centre

Beside the IMP, the following study material will be supplied to the clinical centre:

- final version of the study protocol
- access to the eCRF
- access to IWRS
- copy of the investigator's brochure (IB) relative to the investigational product
- informed consent forms
- subjects' diary cards
- Laboratory manual
- IMP manual
- Laboratory kits

Moreover, before the start of the study, the investigator(s) will be provided with the following documents: ICH guidelines, confidentiality agreement (if applicable), protocol amendments (if any), declaration of Helsinki, insurance statement, SAE forms, financial agreement (if applicable), confidential subject identification code list form, drug accountability forms, investigator and study staff list form.

## 15.2 Protocol amendments

In order to obtain interpretable results, neither the investigator nor the sponsor will alter the study conditions agreed upon and set out in this protocol. Amendments should be made by mutual agreement between the investigator and the sponsor. Any amendment must be set out in writing, giving the reasons, and being signed by all concerned parties. The amendment becomes then part of the protocol.

Non substantial amendments will be notified according to the current regulations.

All amendments will be sent to the EC and concerned Competent Authorities.

## 15.3 Study documentation and record keeping

The investigator should ensure the accuracy, completeness, legibility, and timeliness of the data reported to the sponsor in the eCRF and in all required reports.

The investigator must keep source documents for each subject in the study. All information on the eCRF must be traceable to these source documents, which are generally stored in the subject's medical file. The source documents should contain all demographic and medical information, including laboratory data, etc., and the original signed informed consent forms.

Data reported on the eCRF that are derived from source documents should be consistent with the source documents or the discrepancies should be explained.

The investigator and the sponsor should maintain the study documents as specified in the "Essential Documents for the Conduct of a Clinical Trial" chapter 8 of ICH-GCP and as required by the applicable regulatory requirement(s).

These are documents which individually and collectively permit evaluation of a study and the quality of the data produced and include groups of documents, generated before the study commences, during the clinical study, and after termination of the study and include but are not limited to, study protocol, amendments, submission and approval of EC, raw data of subjects including lab tests, insurance contracts, certificate of analysis of the IMP(s), drug accountability records, signed informed consent forms, confidential subjects identification code, eCRF, curricula vitae of the investigator and other participants in the study, study staff lists and responsibilities, monitoring reports and final study report.

The investigator and the sponsor should take measures to prevent accidental or premature destruction of these documents.

Study documents must be retained by the investigator and the sponsor as long as needed to comply with ICH-GCP, national and international regulations. By signing the protocol, the investigator and the sponsor agree to adhere to these requirements.

### 15.4 Study subjects' recruitment

Study participants will be recruited at the clinical centres.
Participants may be recruited by use of an advertisement +/- registration form formally approved by the ethics committee. In addition, at CRC (site N. 2) subjects already registered in the healthy volunteers database will be contacted.

### 15.5 Confidentiality and data protection

By signing this protocol, the investigators and the CROs agree to keep all the information provided by the sponsor in strict confidentiality and to request the same confidentiality from his/her staff. Study documents provided by the sponsor (protocols, IB, eCRF and other materials) will be stored appropriately to ensure confidentiality. The information provided by the sponsor to the investigators and to the CROs cannot be disclosed to others without direct written authorisation from the sponsor, except for the extent necessary to obtain the informed consent from the subjects wishing to participate in the study.

Data on subjects collected in the eCRF during the study will be documented in a coded way (see § 12.2). If, as an exception, for safety or regulatory reasons identification of a subject becomes necessary, the monitors, the sponsor and the investigators will be bound to keep this information confidential.

## 15.6 Publication policy

The sponsor agrees that the study results (including negative and inconclusive as well as positive results) can be made publicly available by the investigators publishing in peer reviewed journals, presenting results at scientific congresses and posting information and results on internet-based public registers and databases only after the results of the whole study have been published collectively.
Study results will be communicated in full to the competent Health Authorities by the submission of a complete clinical study report.

As the sponsor agrees that the study results can be published by the investigators, these agree to submit any manuscript (abstract, publication, paper, etc.) to the sponsor before any public disclosure.
This will be done in order to ensure that clinical study results are reported in an objective, accurate and balanced manner. The sponsor reviews the proposed manuscripts, before submission, within a reasonable period of time (30-90 days in relation with the complexity of the work).

The investigators will also be provided by the sponsor with the clinical study report and the results of any additional analysis, tables, figures, etc. undertaken for the purposes of the article, in order to take responsibility for the content of the publication(s).
On an exceptional basis, the sponsor may temporarily delay registration of certain data elements (e.g. compound, name, outcome, measures, etc.) to seek necessary intellectual property protection. This is because early disclosure of such data could, in some circumstances, prevent or negatively impact patentability.

# 16 STUDY RESPONSIBLE PERSONS

## 16.1 Sponsor

Toscana Life Sciences Sviluppo s.r.l.,
Via Fiorentina 1, 53100 Siena, Italy
Email: info@toscanalifesciences.org

**Sponsor rappresentatives**
Andrea Paolini,
Direttore Generale
Email: a.paolini@toscanalifesciences.org

Sarah Nosari,
Senior Program Officer
Email: s.nosari@achillesvaccines.com

**Medical Expert on behalf of the Sponsor**
Giovanni Della Cioppa, Clinical R&D Consultant
Email: giodellacioppa@yahoo.co.uk

## 16.2 Data Safety Monitoring Board

Franco Locatelli (presidente)
Medico Presidente del consiglio superiore di Sanità; Direttore del Dipartimento di oncologia, ematologia e medicina trasfusionale Ospedale Bambin Gesù Roma
Email: franco.locatelli@opbg.net.

Sergio Bonini (membro)
Medico Istituto di Farmacologia Traslazionale, Consiglio Nazionale delle Ricerche, Roma, Italia
Email: se.bonini@gmail.com

Paolo Antonio Grossi (membro)
Medico Professore Ordinario di Malattie Infettive Università Insubria – Varese; Esperto di infezione nei trapianti
Email: paolo.grossi@uninsubria.it

Paolo Bruzzi (membro)
Medico Esperto in metodologia e statistica medica, già direttore Dipartimento di epidemiologia clinica, IRCCS AOU San Martino – IST, Genova
Email: bruzzipaolo49@gmail.com

## 16.3 Institutes performing the study

### *16.3.1 Clinical centre N. 1 - Coordinating site*

Istituto Nazionale Malattie Infettive Lazzaro Spallanzani, via Portuense 292, Rome, Italy

Phone: +39.06.5517.0923
Email: simone.lanini@inmi.it

**Principal investigator and National study co-ordinator**
Simone Lanini, MD

### *16.3.2 Clinical centre N 2*

Centro Ricerche Cliniche s.r.l. (CRC) di Verona s.r.l., c/o Policlinico G. B. Rossi, p.le Scuro 10, Verona, Italy

Phone: +39.045.8126509
Email: Stefano.Milleri@crc.vr.it

**Principal investigator**
Stefano Milleri, MD

## 16.4 Pharmacokinetics and ADA assays

Ardena Bioanalysis B.V., W.A. Scholtenstraat 7, NL-9403AJ Assen, the Netherlands
Phone: +31.592.344211
Fax: +31.592.344425
Mobile: +31.649335933
Email: foka.venema@ardena.com

**Analytics representative**
Foka Venema, Senior Project Manager

Analytical facilities and procedures are in compliance with the general principles of GLP regulations.

## 16.5 Serum neutralising power test

VisMederi s.r.l.,
Strada del Petriccio e Belriguardo, 3553100 Siena, Italy
Phone: +31.0577.381253

or

Laboratorio di Virologia
Istituto Nazionale per le Malattie Infettive Lazzaro Spallanzani, IRCCS
Via Portuense 292, 00149 Roma

**Representative**
Concetta Castilletti
Phone: +39.06.55170694
Email: concetta.castilletti@inmi.it

## 16.6 DIESSE kit characterisation

Toscana Life Sciences
Via Fiorentina 1, 53100 Siena, Italy

**Representative**
Emanuele Ardeano
Email: e.andreano@toscanalifesciences.org

## 16.7 Saliva IgG and neutralising power test

VisMederi s.r.l.,
Strada del Petriccio e Belriguardo, 3553100 Siena, Italy
Phone: +31.0577.381253

## 16.8 Co-ordination, data analysis & reporting

CROSS Research S.A.
Via F.A. Giorgioli 14, CH-6864 Arzo, Switzerland
Phone: +41.91.6300510
Fax: +41.91.6300511

**Coordination**
Chiara Lottici, Clinical Project Leader
Email: projectmanagement@croalliance.com

**Medical Writing and Clinical Projects Unit Representative**
Chiara Leuratti, Clinical Projects Unit Head and Senior Medical Writer
Email: medicalwriting@croalliance.com

**Pharmacokinetics expert**
Andrea Di Stefano, Senior Medical Writer
Email: medicalwriting@croalliance.com

**Biometry Unit Representative**
Alessandra Gentili, Biometry Manager, Unit Head
Email: statistics@croalliance.com

**Quality Assurance Unit Representative**
Mario Corrado, Quality Assurance Manager, Unit Head
Email: qau@croalliance.com

## 16.9 Monitoring

### *16.9.1 Clinical site N. 1*

Valentina Greco, Senior Clinical Research Associate (Sr. CRA)
Via della Pineta Sacchetti, 123, 00167 Roma, Italy

Phone: +39.338.858.2160
Fax: +39.06.9933.5444
Fax2: +39.1782273465
Email: v.greco@tiscali.it
Certified Email: valentina.greco@pec.assomonitor.org

### *16.9.2 Clinical site N. 2*

Daniela Sacchi, Clinical Research Associate (CRA)
Via Aldo Villa 50, 20091 Bresso (MI), Italy

Phone: +39.347.851.8359
Email: danisacchi@hotmail.it

## 16.10 Pharmacovigilance

SeQure S.r.l., Via Roveggia, 122, 37136 Verona, Italy

Email: safety@sequrelifesciences.com

**Main Contact**
Alessandra Marchese, Drug Safety Manager & GVP Consultant
Email: alessandra.marchese@sequrelifesciences.com
Phone: +39.069.291.9456

## 16.11 eCRF and IWRS

Advice Pharma Group S.r.l.|
c/o Polihub Politecnico di Milano, Via G. Durando 38, 20158 Milan, Italy
Email: massimo.beccaria@advicepharma.com

# 17 REFERENCES


1. NIH Overview of COVID-19: Epidemiology, Clinical Presentation, and Transmission https://files.covid19treatmentguidelines.nih.gov/guidelines/section/section_9.pdf Last updated : July 17th, 2020.
2. Walsh KA et al. SARS-CoV-2 detection, viral load and infectivity over the course of an infection. J Infect 2020; 81:357-371.
3. Yan X et al. Duration of SARS-CoV-2 viral RNA in asymptomatic carriers. Critical Care 2020; 24:245-247.
4. Andreano E et al. Human monoclonal antibodies for discovery, therapy, and vaccine acceleration. Curr Opin Immunol 2019; 59:130-134.
5. Griffin MP et al. Single-Dose Nirsevimab for Prevention of RSV in Preterm Infants. N Engl J Med 2020; 383:415-425.
6. Wang CY et al. Effect of Anti-CD4 Antibody UB-421 on HIV-1 Rebound after Treatment Interruption. N Engl J Med 2019; 380:1535-1545.
7. Sok D et al. HIV Broadly Neutralizing Antibodies: Taking Good Care of The 98. Immunity 2016; 45:958-960.
8. Gaudinski MR et al. Safety, tolerability, pharmacokinetics, and immunogenicity of the therapeutic monoclonal antibody mAb114 targeting Ebola virus glycoprotein (VRC 608): an open-label phase 1 study. Lancet 2019; 393:889-898.
9. Jiang S et al. Neutralizing Antibodies against SARS-CoV-2 and Other Human Coronaviruses. Trends Immunol 2020; 41:355-359.
10. Baum A et al. REGN-COV2 antibodies prevent and treat SARS-CoV-2 infection in rhesus macaques and hamsters. Science 10.1126/science.abe2402 (2020).
11. Chen P et al. SARS-CoV-2 neutralizing antibody LY-Co555 in outpatients with COVID-19. New Eng J Med October 28, 2020 DOI: 10.1056/NEJMoa2029849.
12. Regeneron Pharmaceuticals. Regeneron's REGN-COV2 antibody cocktail reduced viral levels and improved symptoms in non-hospitalized COVID-19 patients.
13. Investigator's Brochure. Extremely potent human monoclonal antibody for therapy against SARS-CoV-2. Toscana Life Sciences e Sviluppo, Italy. Final version 1.0, 09 December 2020
14. Andreano E et al. Extremely potent human monoclonal antibodies from convalescent COVID-19 patients. bioRxiv preprint doi: https://doi.org/10.1101/2020.10.07.328302. Posted October 2020, not peered reviewed.
15. Korber B, Fischer WM, Gnanakaran S, Yoon H, Theiler J, Abfalterer W, Hengartner N, Giorgi EE, Bhattacharya T, Foley B, et al.: Tracking Changes in SARS-CoV-2 Spike: Evidence that D614G Increases Infectivity of the COVID-19 Virus. Cell 2020:S0092-8674(0020)30820-30825.
16. Bastard P, Rosen LB, Zhang Q, Michailidis E, Hoffmann HH, Zhang Y, Dorgham K, Philippot Q, Rosain J, Béziat V, et al.: Autoantibodies against type I IFNs in patients with life-threatening COVID-19. Science 2020, 370.
17. Zuo Y, Estes SK, Ali RA, Gandhi AA, Yalavarthi S, Shi H, Sule G, Gockman K, Madison JA, Zuo M, et al.: Prothrombotic autoantibodies in serum from patients hospitalized with COVID-19. Sci Transl Med 2020.
18. Lee WS, Wheatley AK, Kent SJ, DeKosky BJ: Antibody-dependent enhancement and SARS-CoV-2 vaccines and therapies. Nat Microbiol 2020, 5:1185-1191.
19. Zalevsky J, Chamberlain AK, Horton HM, Karki S, Leung IWL, Sproule TJ, Lazar GA, Roopenian DC, Desjarlais JR: Enhanced antibody half-life improves in vivo activity. Nature biotechnology 2010, 28:157-159.
20. Schlothauer T, Herter S, Koller CF, Grau-Richards S, Steinhart V, Spick C, Kubbies M, Klein C, Umaña P, Mössner E: Novel human IgG1 and IgG4 Fc-engineered antibodies with completely abolished immune effector functions. Protein Eng Des Sel 2016, 29:457-466.

21. Ackerman ME, Dugast A-S, McAndrew EG, Tsoukas S, Licht AF, Irvine DJ, Alter G: Enhanced phagocytic activity of HIV-specific antibodies correlates with natural production of immunoglobulins with skewed affinity for FcγR2a and FcγR2b. Journal of virology 2013, 87:5468-5476.

22. Mackness BC, Jaworski JA, Boudanova E, Park A, Valente D, Mauriac C, Pasquier O, Schmidt T, Kabiri M, Kandira A, et al.: Antibody Fc engineering for enhanced neonatal Fc receptor binding and prolonged circulation half-life. mAbs 2019, 11:1276-1288.

23. Booth BJ, Ramakrishnan B, Narayan K, Wollacott AM, Babcock GJ, Shriver Z, Viswanathan K: Extending human IgG half-life using structure-guided design. mAbs 2018, 10:1098-1110.

24. Butler AL, Fallon JK, Alter G: A Sample-Sparing Multiplexed ADCP Assay. 2019, 10.

25. Ackerman ME, Mikhailova A, Brown EP, Dowell KG, Walker BD, Bailey-Kellogg C, Suscovich TJ, Alter G: Polyfunctional HIV-Specific Antibody Responses Are Associated with Spontaneous HIV Control. PLoS pathogens 2016, 12:e1005315-e1005315.

26. Karsten CB, Mehta N, Shin SA, Diefenbach TJ, Slein MD, Karpinski W, Irvine EB, Broge T, Suscovich TJ, Alter G: A versatile high-throughput assay to characterize antibody-mediated neutrophil phagocytosis. Journal of immunological methods 2019, 471:46-56.

27. Baum A, Ajithdoss D, Copin R, Zhou A, Lanza K, Negron N, Ni M, Wei Y, Mohammadi K, Musser B, et al.: REGN-COV2 antibodies prevent and treat SARS-CoV-2 infection in rhesus macaques and hamsters. Science 2020.

28. Imai M, Iwatsuki-Horimoto K, Hatta M, Loeber S, Halfmann PJ, Nakajima N, Watanabe T, Ujie M, Takahashi K, Ito M, et al.: Syrian hamsters as a small animal model for SARS-CoV-2 infection and countermeasure development. 2020, 117:16587-16595.

29. Rogers TF, Zhao F, Huang D, Beutler N, Burns A, He W-t, Limbo O, Smith C, Song G, Woehl J, et al.: Isolation of potent SARS-CoV-2 neutralizing antibodies and protection from disease in a small animal model. 2020, 369:956-963.

30. Sia SF, Yan LM, Chin AWH, Fung K, Choy KT, Wong AYL, Kaewpreedee P, Perera R, Poon LLM, Nicholls JM, et al.: Pathogenesis and transmission of SARS-CoV-2 in golden hamsters. Nature 2020, 583:834-838.

31. EMEA/CHMP/SWP/28367/07 Rev. 1. Guideline on strategies to identify and mitigate risks for first-in-human and early clinical trials with investigational medicinal products. 20 July 2017

32. Monoclonal antibodies. Meyler's Side Effects of Drugs (16th edition) 2016. 1100-1102.

33. EMEA/CHMP/BMWP/14327/2006 Rev 1. Guideline on Immunogenicity assessment of therapeutic proteins. 18 May 2017

34. Phoenix 1.3 User’s Guide, Pharsight Corporation

35. SAS/STAT® User’s Guide

36. US Food and Drug Administration. Guidance for industry: toxicity grading scale for healthy adult and adolescent volunteers enrolled in preventive vaccine clinical trials. Rockville, MD: Center for Biologics Evaluation and Research; September 2007

37. Addendum Investigator's Brochure. Extremely potent human monoclonal antibody for therapy against SARS-CoV-2. Toscana Life Sciences e Sviluppo, Italy. Final version 1.0, 25JAN21

38. Wadman M: Eli Lilly reports promising first results for an antibody against COVID-19. Science, https://www.sciencemag.org/news/2020/09/eli-lilly-reports-first-promising-results-antibody-against-covid-19. Accessed on 25JAN21

39. Regeneron Pharmaceuticals I: REGENERON'S REGN-COV2 ANTIBODY COCKTAIL REDUCED VIRAL LEVELS AND IMPROVED SYMPTOMS IN NON-HOSPITALIZED COVID-19 PATIENTS. https://investor.regeneron.com/news-releases/news-release-details/regenerons-regn-cov2-antibody-cocktail-reduced-viral-levels-and. Accessed on 25JAN21
