# Supplementary material for: Safety and serum distribution of anti-SARS-CoV-2 monoclonal antibody MAD0004J08 after intramuscular injection
Source: Nat Commun. 2022 Apr 27;13:2263. doi: 10.1038/s41467-022-29909-x (PMC9046195; doi:10.1038/s41467-022-29909-x)
Supplement: Supplementary file 3 — Reporting Summary [file 41467_2022_29909_MOESM3_ESM.pdf]

nature research

Corresponding author(s): Rino Rappuoli

Last updated by author(s): Mar 13, 2022

Reporting Summary

Nature Research wishes to improve the reproducibility of the work that we publish. This form provides structure for consistency and transparency in reporting. For further information on Nature Research policies, see our Editorial Policies and the Editorial Policy Checklist.

Statistics

For all statistical analyses, confirm that the following items are present in the figure legend, table legend, main text, or Methods section.

n/a	Confirmed	
☐	☒	The exact sample size (*n*) for each experimental group/condition, given as a discrete number and unit of measurement
☐	☒	A statement on whether measurements were taken from distinct samples or whether the same sample was measured repeatedly
☐	☒	The statistical test(s) used AND whether they are one- or two-sided<br>*Only common tests should be described solely by name; describe more complex techniques in the Methods section.*
☒	☐	A description of all covariates tested
☒	☐	A description of any assumptions or corrections, such as tests of normality and adjustment for multiple comparisons
☐	☒	A full description of the statistical parameters including central tendency (e.g. means) or other basic estimates (e.g. regression coefficient) AND variation (e.g. standard deviation) or associated estimates of uncertainty (e.g. confidence intervals)
☐	☒	For null hypothesis testing, the test statistic (e.g. F, t, r) with confidence intervals, effect sizes, degrees of freedom and P value noted<br>*Give P values as exact values whenever suitable.*
☒	☐	For Bayesian analysis, information on the choice of priors and Markov chain Monte Carlo settings
☒	☐	For hierarchical and complex designs, identification of the appropriate level for tests and full reporting of outcomes
☒	☐	Estimates of effect sizes (e.g. Cohen's d, Pearson's r), indicating how they were calculated

Our web collection on statistics for biologists contains articles on many of the points above.

Software and code

Policy information about availability of computer code

Data collection	- BioTek Gen5 Data Analysis Software v3.0
Data analysis	- GraphPad Prism 8.0.2 was used to perform statistical analyses

For manuscripts utilizing custom algorithms or software that are central to the research but not yet described in published literature, software must be made available to editors and reviewers. We strongly encourage code deposition in a community repository (e.g. GitHub). See the Nature Research guidelines for submitting code & software for further information.

Data

Policy information about availability of data

All manuscripts must include a data availability statement. This statement should provide the following information, where applicable:

- Accession codes, unique identifiers, or web links for publicly available datasets
- A list of figures that have associated raw data
- A description of any restrictions on data availability

Source data are provided with this paper. All data supporting the findings in this study are available within the article, Supplementary Information or can be obtained from the corresponding author upon request. SARS-CoV-2 sequences are accessible within the global initiative on sharing all influenza data (GISAID) database.

Field-specific reporting

Please select the one below that is the best fit for your research. If you are not sure, read the appropriate sections before making your selection.

- [x] Life sciences
- [ ] Behavioural & social sciences
- [ ] Ecological, evolutionary & environmental sciences

For a reference copy of the document with all sections, see nature.com/documents/nr-reporting-summary-flat.pdf

Life sciences study design

All studies must disclose on these points even when the disclosure is negative.

Sample size	A total of 30 healthy men and women (10 in each cohort) will be enrolled in the study. Statistical analyses to determine sample size was not performed, as it was not required from regulatory agencies for a phase I clinical trial. The study sponsor, in accordance with regulatory agencies, believed that 10 subjects/cohort were sufficient to meet the endpoint of the study.
Data exclusions	Subjects considered placebo on the base of the absence of antibody detection in their sera were excluded from the data aimed to assess the serum neutalization activity. Two subjects per group were excluded.
Replication	All experiments were performed in technical duplicates as indicated in the methods section. All attempts at replication were successful.
Randomization	Within each cohort subjects were randomized with 4:1 ratio to a single i.m. dose of MAD0004J08 (48 mg in Cohort 1, 100 mg in Cohort 2, and 400 mg in Cohort 3) or placebo using an interactive web response system (IWRS).
Blinding	The study will be double-blind within each cohort, i.e. all study subjects, site personnel, monitors, sponsor, contract research organization (CRO) study teams and DSMB will be blinded to the treatment given to each subject.

Reporting for specific materials, systems and methods

We require information from authors about some types of materials, experimental systems and methods used in many studies. Here, indicate whether each material, system or method listed is relevant to your study. If you are not sure if a list item applies to your research, read the appropriate section before selecting a response.

Materials & experimental systems

n/a	Involved in the study	
[ ]	[x]	Antibodies
[ ]	[x]	Eukaryotic cell lines
[x]	[ ]	Palaeontology and archaeology
[x]	[ ]	Animals and other organisms
[ ]	[x]	Human research participants
[ ]	[x]	Clinical data
[x]	[ ]	Dual use research of concern

Methods

n/a	Involved in the study	
[x]	[ ]	ChIP-seq
[x]	[ ]	Flow cytometry
[x]	[ ]	MRI-based neuroimaging

Antibodies

Antibodies used	Sigma Alkaline phosphatase labelled anti-Human IgG (γ chain specific), Cat#A3187, polyclonal, dilution used 1:2,000
Validation	Sigma Alkaline phosphatase labelled anti-Human IgG (γ chain specific), Cat#A3187, polyclonal, reactivity human, application ELISA (https://www.sigmaaldrich.com/IT/en/product/sigma/a3187).

Eukaryotic cell lines

Policy information about cell lines

Cell line source(s)	VERO E6 cell line ATCC Cat#CRL-1586
Authentication	VERO E6 cell line was obtained from vendors that sell authenticated cell lines, they grew, performed and showed morphology as expected. No additional specific authentication was performed.
Mycoplasma contamination	Vero E6 cell lines are routinely tested on a monthly basis and tested negative for mycoplasma.
Commonly misidentified lines (See ICLAC register)	No commonly misidentified cell lines were used in this study.

Human research participants

Policy information about studies involving human research participants

Population characteristics	This is a dose escalation study, open label across doses and randomized, double blind withing each dose level. A total of 30 healthy men and nonpregnant women, 18 to 55 years of age, meeting all inclusion/exclusion criteria were enrolled in three sequential cohorts of 10 subjects each. Within each cohort subjects were randomized with 4:1 ratio to a single i.m. dose of MAD0004J08 (48 mg in Cohort 1, 100 mg in Cohort 2, and 400 mg in Cohort 3) or placebo using an interactive web response system (IWRS). Within each cohort subjects were grouped in two groups of five: the five subjects of the first group, referred to as "sentinels" were randomized one at a time at 48-hour intervals, assuming no safety concern in the investigator's judgement; the five subjects of the second group were randomized and enrolled with no time restriction.<br>Covariate-relevant population characteristics of the human research partecipants for serum specimens are:<br>- Cohort 1 (48 mg), age min/max 19-51, 50% males (n=5) and 50% female (n=5), 100% (n=10) white<br>- Cohort 2 (100 mg), age min/max 21-33, 60% males (n=6) and 40% female (n=4), 100% (n=10) white<br>- Cohort 3 (400 mg), age min/max 27-54, 60% males (n=6) and 40% female (n=4), 100% (n=10) white
Recruitment	Inclusion criteria were as follows: age 18-55, signed informed consent, willingness to use appropriate contraception, body mass index- 18.5-30 kg/m2; systolic blood pressure 90-139 mmHg, diastolic blood pressure 69-90 mmHg; heart rate 50-100 bpm; electrocardiogram (ECG) without clinically significant abnormalities; negative SARS-CoV-2 serology test (negative anti-S and anti-N) or negative SARS-CoV-2 qRT-PCR in the 72h prior with result before the treatment.<br>Exclusion criteria were as follows: prior intake of investigational or licensed vaccine for the prevention of SARS CoV2; history of infection with SARS or MERS; positive or missing pregnancy test at screening or day 1 or lactating women; history of allergic reactions likely to be exacerbated by any component of the investigational product; previous intake of a mAb within 6 months; history of malignancy in the last 5-7 years; Immunodeficiency due to illness, any course of glucocorticoid therapy exceeding 2 weeks; acute illnesses- history of renal, hepatic, gastrointestinal, cardiovascular, respiratory, dermatologic , hematological, endocrine, psychiatric or neurological diseases that may interfere with the aim of the study or increase subjects risks in investigator's opinion. A statement was included in the study protocol that if during the study, a subject is included in a vaccination list according to the national guidelines, the best option for the subject will be pursued.<br>Study participants were recruited at the clinical centres by use of an advertisement and by using a registration form formally approved by the ethics committee. The authors report no self-selection bias for this study.
Ethics oversight	The final protocol and informed consent were approved by the institutional review boards of each of the participating investigational sites. This study is designed and conducted in accordance with the Declaration of Helsinki, the current revision of Good Clinical Practice (GCP), ICH topic E6 (R2), and the applicable local law requirements. Our phase 1, first in human (FIH) trial was performed at two sites in Italy (Istituto Nazionale Malattie Infettive Lazzaro Spallanzani, Rome, and Centro Ricerche Cliniche di Verona s.r.l. (CRC), Verona). The final protocol and informed consent were approved by the institutional review boards of each of the participating investigational sites. The study was approved by the "Comitato Etico Unico dell'Istituto nazionale per le malattie infettive (INMI) Lazzaro Spallanzani", Rome (IT), ethic committee.

Note that full information on the approval of the study protocol must also be provided in the manuscript.

Clinical data

Policy information about clinical studies

All manuscripts should comply with the ICMJEguidelines for publication of clinical research and a completedCONSORT checklist must be included with all submissions.

Clinical trial registration	The trial is registered with EudraCT N.: 2020-005469-15 and ClinicalTrials.gov Identifier: NCT04932850.
Study protocol	Protocol can be accessed at https://clinicaltrials.gov/ct2/show/study/NCT04932850
Data collection	Clinical data required by the study protocol are carefully reported in the electronic case report form (eCRFs), including data inserted by the subjects in the study diary. The investigator must also check that the data reported in the eCRFs correspond to those in the subject's source documents, including subject's diary data. Data collected up to 48h (for Cohorts 1, 2 and 3) (all subjects) must be reported in the eCRF within 24h from the collection. Recruitment for this study occurred between the 24th of February 2021 and 21st of April 2021. Data were collected from the 1st of March 2021 to the 20th of January 2022.
Outcomes	Primary end point: Proportion of subjects with severe and/or serious treatment-emergent adverse events (TEAEs), including clinically relevant laboratory abnormalities, vital signs, and adverse reactions at the injection site) in the 7 days post-treatment. A TEAE is defined as any AE with onset after administration of study drug (N=30).<br>Secondary end points: Proportion of subjects with solicited local AEs (pain, redness and swelling at injection site) and systemic AEs (headache, fatigue, muscle pain, joint pain, vomiting, diarrhea, chills, fever) from day 1 to day 7 (N=30); MAD0004J08 sera concentrations on day 1 (0, 1, 2, 3, 4, 6, 8, 12, and 24h), days 2, 8, 15, 22, 30 (N=24; subjects considered placebo were excluded from the analyses); MAD0004J08 sera neutralizing ability at baseline, and on days 2, 8, and 30 (N=24; subjects considered placebo were excluded from the analyses).
